# Assessing the risk of ketoacidosis due to sodium-glucose cotransporter (SGLT)-2 inhibitors in patients with type 1 diabetes: A meta-analysis and meta-regression

**DOI:** 10.1371/journal.pmed.1003461

**Published:** 2020-12-29

**Authors:** Giovanni Musso, Antonio Sircana, Francesca Saba, Maurizio Cassader, Roberto Gambino

**Affiliations:** 1 HUMANITAS Gradenigo, Turin, Italy; 2 Department of Cardiology, Azienda Ospedaliero Universitaria, Sassari, Italy; 3 Laboratory of Diabetes and Metabolic disorders, Department of Medical Sciences, University of Turin, Turin, Italy; Shanghai Jiao Tong University Affiliated Sixth People’s Hospital, CHINA

## Abstract

**Background:**

Sodium-glucose cotransporter-2 (SGLT2) inhibitors (SGLT2i) showed benefits in type 1 diabetes mellitus (T1DM), but the risk of diabetic ketoacidosis (DKA) limits their use. Ability to predict DKA risk and therapeutic responses would enable appropriate patient selection for SGLT2i. We conducted a meta-analysis and meta-regression of randomized controlled trials (RCTs) evaluating SGLT2i in T1DM to assess moderators of the relative risk (RR) of DKA, of glycemic (HbA1c, fasting plasma glucose, continuous glucose monitoring parameters, insulin dose, and insulin sensitivity indices) and non-glycemic (body mass index (BMI), systolic BP, renal function, albuminuria, and diabetic eye disorders) efficacy, and of other safety outcomes (including hypoglycemia, infections, major adverse cardiovascular events, and death).

**Methods and findings:**

We searched MEDLINE, Cochrane Library, EMBASE, ClinicalTrials.gov, Cochrane CENTRAL Register of Controlled Trials, and other electronic sources through August 30, 2020, for RCTs comparing SGLT2i with active comparators or placebo in adult patients with T1DM. Reviewers extracted data for relevant outcomes, performed random effects meta-analyses, subgroup analyses, and multivariable meta-regression. The strength of evidence was summarized with the GRADE approach. Among 9,914 records identified, 18 placebo-controlled RCTs (7,396 participants, 50% males, mean age 42 y (range 23 to 55 y), 5 different SGLT2i evaluated), were included. Main outcome measures were effect sizes and moderators of glycemic and non-glycemic efficacy and of safety outcomes. In a multivariable meta-regression model, baseline BMI (β = 0.439 [95% CI: 0.211, 0.666], *p* < 0.001) and estimated glucose disposal rate (eGDR) (β = −0.766 [−1.276, −0.256], *p* = 0.001) were associated with the RR of DKA (RR: 2.81; 95% CI:1.97, 4.01; *p* < 0.001, R^2^ = 61%). A model including also treatment-related parameters (insulin dose change-to-baseline insulin sensitivity ratio and volume depletion) explained 86% of variance across studies in the risk of DKA (R^2^ = 86%). The association of DKA with a BMI >27 kg/m^2^ and with an eGDR <8.3 mg/kg/min was confirmed also in subgroup analyses. Among efficacy outcomes, the novel findings were a reduction in albuminuria (WMD: −9.91, 95% CI: −16.26, −3.55 mg/g, *p* = 0.002), and in RR of diabetic eye disorders (RR: 0.27[0.11, 0.67], *p* = 0.005) associated with SGLT2i. A SGLT2i dose-response gradient was consistently observed for main efficacy outcomes, but not for adverse events (AEs). Overall, predictors of DKA and of other AEs differed substantially from those of glycemic and non-glycemic efficacy. A limitation of our analysis was the relatively short (≤52 weeks) duration of included RCTs. The potential relevance for clinical practice needs also to be confirmed by real-world prospective studies.

**Conclusions:**

In T1DM, the risk of DKA and main therapeutic responses to SGLT2i are modified by baseline BMI and insulin resistance, by total insulin dose reduction-to-baseline insulin sensitivity ratio, and by volume depletion, which may enable the targeted use of these drugs in patients with the greatest benefit and the lowest risk of DKA.

## Abstract 2

Giovanni Musso and colleagues conduct a meta-analysis to identify risk factors of diabetic ketoacidosis in patients with Type 1 diabetes taking SGLT2 inhibitors.

## Author summary

**Why was this study done?:**

- Sodium-glucose cotransporter-2 inhibitors (SGLT2i) are recommended for type 2 diabetes for their substantial glycemic and non-glycemic benefits.
- In type 1 diabetes mellitus (T1DM), their use is significantly limited by the increased risk of diabetic ketoacidosis (DKA), a serious adverse event (AE).
- Currently, there are no data on factors predicting the risk of DKA and therapeutic response to SGLT2i in T1DM.
- Knowing predictors of the risk of DKA and of therapeutic response would enable T1DM patient selection with the highest benefit-to-risk ratio from SGLT2i treatment.

**What did the researchers do and find?:**

- We explored moderators of the risk of DKA and of main efficacy and safety outcomes associated with SGLT2i treatment in a meta-analysis and multiple meta-regression of 18 randomized trials enrolling 7,396 T1DM patients treated with SGLT2i or placebo.
- In a multivariate meta-regression model, the risk of SGLT2i-associated DKA was largely explained by 4 independent parameters: BMI >27 kg/m^2^, an estimated glucose disposal rate (eGDR) <8.3 mg/kg/min, indicative of insulin resistance, a higher total insulin dose reduction-to-baseline insulin sensitivity ratio, and dehydration.
- The factors associated with therapeutic effectiveness differed substantially from risk factors of DKA and of other AEs.
- A dose-response relationship with increasing SGLT2i doses was observed for main efficacy outcomes, but not for the risk of DKA and for other AEs.

**What do these findings mean?:**

- in T1DM, the risk of DKA risk and therapeutic responses SGLT2i treatment are predictable using clinically available parameters.
- Predictors of the risk of DKA and other AEs radically differ from those of therapeutic effectiveness.
- If confirmed by real-world prospective studies, the results of our analysis may enable the targeted use of SGLT2i in patients with T1DM who have the greatest benefit and the lowest risk of DKA from the use of these drugs.

## Introduction

The prevalence of type 1 diabetes mellitus (T1DM) is rising at a yearly rate of approximately 3% [1,2]. Patients with T1DM achieve glycemic targets in only 30% of cases and suffer from the side effects of insulin therapy [3]. Hence, several adjunctive therapies to insulin have been proposed to satisfy the largely unmet medical need of this patient population. Sodium-glucose cotransporter-2 (SGLT2) inhibitors (SGLT2i) block the SGLT-2 transporter in the proximal renal tubule, resulting in glycosuria and natriuresis [4]. SGLT2i are now recommended for patients with type 2 diabetes mellitus (T2DM) and showed glycemic and non-glycemic benefits in T1DM as well, including improved glycemic control and glycemic variability, decreased insulin dose requirement, and blood pressure and body weight reduction [5,6].

In T1DM, however, SGTL2i treatment has to be weighed against an increased risk of diabetic ketoacidosis (DKA) [7,8], a serious, life-threatening adverse event (AE), which dominates the safety profile of these drugs. The risk of DKA varies widely across different randomized controlled trials (RCTs), and factors underlying such variation in DKA risk are unknown.

This uncertainty is reflected by the diverse positions of drug regulatory agencies, with the same SGLT2i being either approved an adjunct to insulin in patients with T1DM inadequate glycemic control [9,10], or approved with restriction to patients with a body mass index (BMI) ≥27 kg/m^2^ [11–13] despite the lack of data regarding the risk of DKA in overweight individuals, or rejected because of the risk of DKA deemed excessively high [14,15]. An evidence-based precision medicine tool to stratify T1DM patients for individual benefit-risk ratio of SGLT2i use is unavailable, as is a systematic review of the evidence to assess predictors of the risk of DKA, which could enable a safer use of these drugs in T1DM [16–18].

We conducted a meta-analysis and meta-regression of RCTs in T1DM to explore factors associated with the risk of DKA and with other efficacy and safety outcomes in adults treated with SGLT2i.

## Methods

### Data sources and searches

We searched English and non-English language publications through August 30, 2020. A full list of electronic databases and clinical trial registries is reported in S1 Text.

No language restrictions were applied. We also searched drug regulatory agencies’ and drug manufacturers’ websites for relevant documents, and the American Diabetes Association (ADA) and European Association for the Study of Diabetes (EASD) meeting abstracts, which were subjected to the same assessment as regular articles [19–21].

We contacted authors by e-mail to verify results and methodological quality of retrieved articles and drug manufacturers to inquire about further published and unpublished trials. Additionally, we manually scanned reference lists from trials, review articles, and reports to identify any other relevant data.

### Search terms and search strategies

The search terms and examples of search strategies are provided in S1 Text.

### Study selection

Inclusion criteria: English and non-English articles reporting RCTs with participants aged >18 y, of any sex or ethnic origin, comparing SGLT2i with placebo or active comparators as adjunct therapy to insulin in T1DM.

Exclusion criteria were: nonhuman studies, noncontrolled or nonrandomized trials, letters/case reports, and articles not reporting outcomes of interest or primary data (editorials and reviews).

### Outcomes

#### Primary outcome

We were primarily interested in exploring the association between the RR of definite DKA (see S1 Text) [18,22] and different study-level moderators. To this aim, we performed a meta-analysis followed by univariable and multivariable meta-regression.

We conducted the same analyses for secondary outcomes, which were grouped into 3 broad sets (glycemic efficacy, non-glycemic efficacy, and safety outcomes other than DKA) and are tabulated in Table 1 and detailed in S1 Text [23–30].

**Table 1:** Glycemic and non-glycemic efficacy outcomes and safety outcomes evaluated in the meta-analysis.

**Glycemic efficacy outcomes**	
**Outcome**	**Comments/description**
**HbA1c (%)**	changes in HbA1c (%) from baseline
**Fasting plasma glucose (FPG)**	changes in FPG (mg/dL) from baseline
**Time-in-range 70–180 mg/dL (%)**	% of daily glucose readings between 70 and 180 mg/dL over each 24-h period during continuous glucose monitoring (CGM)
**Mean amplitude of glucose excursion (MAGE) (mg/dL)**	average of all glucose excursion that exceeded 1 SD over each 24-h period during CGM. MAGE is an index of glycemic variability.
**Urinary glucose excretion (UGE, g/24 h)**	daily urinary glucose excretion
**Daily total insulin dose (TID) changes (%)**	[(end-of treatment TID-initial TID)/initial TID] × 100%
**Daily basal insulin dose (ID) changes (%)**	[(end-of treatment basal ID-initial basal ID)/initial basal ID] × 100%
**Daily bolus ID changes (%)**	[(end-of treatment bolus ID-initial bolus ID)/initial bolus ID] × 100%
**Estimated glucose disposal rate (eGDR) changes (%)**	[(end-of treatment eGDR-initial eGDR)/initial eGDR] × 100%
**Relative insulin sensitivity (RIS) changes (%)**	[(end-of treatment RIS-initial RIS)/initial RIS] × 100%
**Non-glycemic efficacy outcomes**	
**Outcome**	**Comments/description**
**BMI changes (%)**	[(end-of treatment BMI-initial BMI)/initial BMI] × 100%
**SysBP changes (mmHg)**	[(end-of treatment sysBP-initial sysBP)/initial sysBP]
**eGFR changes (ml/min/1.73 m**^**2**^**)**	[(end-of treatment eGFR-initial eGFR)/initial eGFR]
**ACR changes (mg/g)**	[(end-of treatment ACR-initial ACR)/initial ACR]
**Diabetic eye disorders**	including development of hemorrhagic retinopathy/vitreous hemorrhage, retinal detachment, macular edema), glaucoma, or vision loss (as defined by the International Clinical Disease Severity Scale [29])
**Safety outcomes**	
**Definite diabetic ketoacidosis (DKA)**	anion-gap metabolic acidosis with ketone increase without a satisfactory alternative cause for anion-gap acidosis [18,22,26]
**Hypoglycemia**	blood glucose <70 mg/dL [26]
**Severe hypoglycemia (SH)**	SH was defined as an event consistent with hypoglycemia when any of the following 3 conditions occurred [26]: suspected hypoglycemia treated with carbohydrate or glucagon that required the assistance of others, due to neurologic impairment. the patient lost consciousness during the episode; the patient had a seizure during the episode.
**Urinary tract infections (UTIs)**	-
**Genital tract infections (GTIs)**	-
**Upper respiratory infections**	-
**MACE**	cardiovascular death, myocardial infarction, stroke, hospitalization due to heart failure or unstable angina, or coronary revascularization
**Limb amputation**	-
**Bone fracture**	-
**Gastrointestinal events: nausea, vomiting, diarrhea**	-
**Renal events**	defined according to the Medical Dictionary for Regulatory Activities (MedDRA) preferred items version 15.1 (S1 Text)
**Volume depletion events**	defined according to the Medical Dictionary for Regulatory Activities (MedDRA) preferred items version 15.1 (S1 Text)
**Drug-induced liver injury**	-
**Venous thromboembolism**	-
**Cancer**	-
**Serious adverse event (AE)**	any untoward medical occurrence that results in death, life threat, patient hospitalization, a persistent or significant incapacity or substantial disruption of the ability to conduct normal life functions, or if that requires medical intervention to prevent one of the outcomes listed above
**All-cause death**	-

ACR, albumin-to-creatinine ratio; eGFR, estimated glomerular filtration rate; ID, insulin dose; MACE, major adverse cardiovascular events; MAGE, mean amplitude of glucose excursions; TID, total daily insulin dose.

#### Data extraction and risk-of-bias assessment

Two reviewers (GM and RG) extracted data independently and in duplicate by using a predefined electronic data collection form, based on the Cochrane Handbook for Systematic Reviews of Intervention; discrepancies were arbitrated by a third reviewer and resolved by consensus. The agreement between the 2 reviewers for selection and validity assessment of trials was scored by Kappa coefficient.

The quality of RCTs was assessed by the Cochrane Collaboration Risk-of-Bias (RoB) Tool [31]. Sponsorship bias was also included in the RoB tool.

Rather than equating industry sponsorship with high risk of bias and automatically downgrading the evidence for industry sponsorship [32], for included trials we systematically assessed a prespecified list of 8 items in trial designing, conducting and reporting, which have been empirically linked to the risk of biased outcomes in industry-funded trials and are not included in the 6 domains of the RoB tool [33–39] (**Table A in** S1 Tables).

Different doses of SGLT2i were classified into high-, moderate-, or low-dose subgroups based on the potency of the drugs and the dose categorization adopted in clinical trials [4] (S1 Text).

When trials evaluated different SGLT2i doses, we presented data separately for each dose arm and split sample size of the placebo group evenly among different dose comparisons (Cochrane Handbook for Systematic Reviews of Intervention, chapter 7.6 to 7.8 and 16.1.3). For the same RCT reported by several publications on different follow-up periods, the longest follow-up period was assessed.

Publication bias was examined using funnel plots and the Egger test.

### Data synthesis, analysis

The analysis was carried out in concordance with the Cochrane Handbook of Systematic Reviews of Interventions [31] using RevMan Version 5.3.5 (Nordic Cochrane Center, Copenhagen, Denmark) [40] and Stata, release 11.2 (StataCorp, College Station, Texas) and was reported according to PRISMA guidelines (S1 Text) [41]. Treatments were evaluated on an intention-to-treat principle.

We calculated weighted mean differences (WMDs) and 95% CIs for continuous outcomes using an inverse variance random-effects model. For dichotomous outcomes, we calculated risk ratios (RRs) and 95% CIs by using the random-effects Mantel–Haenszel approach with significance set at *P* = 0.05. We conservatively used a random-effects model assuming a substantial variability in treatment effect size across studies.

Statistical heterogeneity was quantified using the I^2^ statistic with values of 25%, 50%, and 75% consistent with low, moderate, and high degrees of heterogeneity, respectively [42]: with I^2^ values ≥50%, we planned to explore individual study characteristics and those of subgroups of the main body of evidence.

### Subgroup analyses

We also planned a priori subgroup analysis to explore potential effects on outcome measures of the following conditions:

- duration of diabetes (<20 versus ≥20 y);
- baseline HbA1c levels (>8% versus ≤8%);
- baseline BMI (>27 versus ≤27 kg/m^2^);
- baseline insulin resistance, defined by an estimated glucose disposal rate (eGDR) <8.3 versus ≥8.3 mg/kg/min) [25];
- renal function stage: inclusion or exclusion of patients with impaired renal function (eGFR <60 ml/min/1.73 m^2^);
- study duration: <24 versus ≥24 weeks;
- background therapy (pretreatment insulin optimization versus stable insulin therapy);
- additionally, we planned to explore potential differences among individual SGLT2i by conducting separate analyses for each drug when sufficient data were available.

### Dose-response analysis

We explored interactions between different SGLT2i doses and all outcomes primarily by comparing different dose groups within head-to-head trials, as the within-trial approach has a lower risk of ecological bias than the across-trial approach [43]; we verified robustness of this approach in ruling out dose-response relationship by using also an across-trial comparison and meta-regression. The “across-trial” approach has a higher risk of ecological bias but a higher power than the within-trial approach, thus allowing ruling out dose-response interactions with higher confidence.

### Grading of evidence

The Grading of Recommendations Assessment, Development, and Evaluation (GRADE) approach was employed to assess the strength of evidence at outcome level and relative confidence in summary estimates [44]. Inconsistency, risk-of-bias, indirectness, imprecision, and publication bias for evidence related to 7 efficacy outcomes (HbA1c, time-in-range, eGDR, BMI, sysBP, eGFR, and albuminuria) and 7 safety outcomes [DKA, severe hypoglycemia, urinary tract infections (UTIs), genital tract infections (GTIs), eye disorders, volume depletion, and major adverse cardiovascular events (MACE)] were quantified.

### Meta-regression analyses

When ≥8 comparisons were available (Cochrane Handbook of Systematic Reviews of Intervention (chapter 9.6.4)), the effect of different study level moderators on each outcome were assessed by meta-regression analysis (random effects model, within-study variance estimated with the restricted maximum-likelihood method, and Knapp–Hartung adjustment applied to compute SEs of the estimated coefficients to calculate summary effect estimates [45,46]). We specified a priori study level moderators based on existing literature and explored novel factors based on known pathophysiological data [16–18].

In meta-regression of the primary outcome (DKA), we classified moderators into baseline risk factors (to identify baseline risk factors for subsequent DKA) and treatment-related risk factors (to assess the moderating effects of treatment-related changes in different parameters on the risk of DKA), although overlaps were expected.

### Baseline risk factors of incident DKA

Baseline risk factors included patient-related factors [age, gender, ethnicity (% white/Asian/Hispanic/black versus other ethnicities), continuous subcutaneous infusion (CSI) users (%), total insulin dose (TID) (IU/d), diabetes duration, BMI, HbA1c, fasting plasma glucose (FPG), eGDR, renal function stage, fasting blood β-hydroxybutyrate (BHB) level], and study design-related factors [study duration, study sample size, SGLT2i dose, SGLT2i drug, pre-randomization insulin optimization versus no optimization, risk-of-bias (high/unclear versus low)].

Treatment-related risk factors of DKA were: first, parameters regarding insulin dose adjustment, as excessive insulin dose reduction is a key contributor to DKA, but the exact extent of insulin dose down-titration increasing the risk of DKA is unclear. We evaluated the association of the risk of DKA with the following parameters: total/basal/bolus ID changes (% initial ID), ratio of changes in TID to baseline BMI (d-TID/BMI) or to baseline relative insulin sensitivity (RIS) (d-TID/baseline RIS). We hypothesized that, rather than the absolute insulin dose reduction, the risk of DKA could depend on the extent of TID reduction relative to initial insulin resistance: The impact of a given insulin dose down-titration on ketogenesis would be expected to be larger in the presence of higher baseline insulin resistance, which would enhance unrestricted lipolysis of free fatty acids (FFAs) from adipose tissue to the liver to fuel ketogenesis. This effect could be captured by the ratio of TID reduction to baseline RIS or BMI better than by % reduction in initial insulin dose. Second, residual insulin-SGLT2 inhibitor (INS-SGLT2i) effect, defined as the difference between the observed TID reduction and the predicted TID reduction (expressed as % of baseline TID) calculated from the 24-hour urinary glucose excretion (see S1 Text) [47]. Third, changes in the following parameters: BMI (%), HbA1c (%), FPG (mg/dL), insulin sensitivity (eGDR and RIS change), fasting blood BHB level, eGFR changes, volume depletion events, UTIs, GTIs, and respiratory infections.

Categorical variables were included in the model by means of dummy variables. SGLT2i dose variable in the regression equation was treated categorically, with the lowest dose coded as the baseline amount and moderate and high doses with a single increment increase.

Due to the considerable number of covariates, permutation test (using 1,000 reallocations) was used for assessing the true statistical significance of an observed meta-regression finding [48]. Moderators that were significant at univariable analyses were included in a multivariable meta-regression model. To measure the strength of a moderator, we compared the meta-regression models with the meta-analysis without covariates and estimated the percentage of heterogeneity explained by a moderator. The index R^2^ value (defined as the ratio of explained to total variance) was used to determine the proportion of variance accounted for by different moderators.

We tested 3 different meta-regression models: model 1 (including baseline predictors that were significantly associated with the risk of DKA at univariable analysis), which identifies moderators of the risk of subsequent development of SGLT2i-associated DKA; model 2 (including treatment-related factors associated with DKA at univariable analysis), which identifies moderators of the risk of DKA during SGLT2i treatment; model 3 (including both baseline and treatment-related moderators of the risk of DKA), which accounts for interactions between baseline and treatment-related moderators and provide an overall ability to predict the across study variance in the RR of DKA.

We planned to conduct the same meta-regression analyses for other outcomes (HbA1c, BMI, systolic BP, eGFR, ACR, diabetic eye disorders, severe hypoglycemia, UTI, GTI, and volume depletion) to obtain a thorough profile of moderators of SGLT2i efficacy and safety in T1DM.

### Sensitivity analyses

We planned to verify consistency and robustness of our findings by repeating the meta-analysis and meta-regression with alternative effect measures (odds ratio (OR) versus RR), pooling methods (Peto’s method versus Mantel–Hanszel, as Peto’s OR is less biased and most powerful at event rates below 1%) [49], statistical models (fixed versus random effects), by excluding RCTs where we imputed values and RCTs at high risk of bias in any domains of the RoB tool.

We also reran Model 1 and Model 2 as fully adjusted multivariable models, including all candidate moderators, with the upper limit number of moderators for each model set at n/2 (where n is the number of observations), with statistical significance set at 0.15 to select variables from Model 1 and 2 to be included in Model 3 [50,51].

Finally, a leave-one-out sensitivity analysis was also performed by repeating the meta-analysis and meta-regression, each time with one of the included studies omitted, to see whether any one study had a disproportionately large influence on the effect estimates.

### Management of missing data

We planned to manage missing data by contacting via e-mail the corresponding authors. Where this was unsuccessful, we planned to follow the approach described in Cochrane Handbook of Systematic Reviews of Intervention (chapter 7.6 to 7.8 and 16.1.3) [31] (S1 Text).

The protocol of the meta-analysis was submitted as a module assignment for the Systematic Review module and internally peer-reviewed at HUMANITAS University Gradenigo Hospital Institutional Review Board and is available at our Institution at request (e-mail: direzione.sanitaria@gradenigo.it).

The protocol of the meta-analysis was registered at the Joanna Briggs Institute (JBI) SYSTEMATIC REVIEW PUBLIC REGISTER (registration number: 2020-04-17).

### Ethics statement

The protocol was approved by Humanitas Gradenigo’s review board and approved on January 20, 2020. The entire protocol is available upon request by e-mail to the corresponding author.

## Results

**Fig 1:**
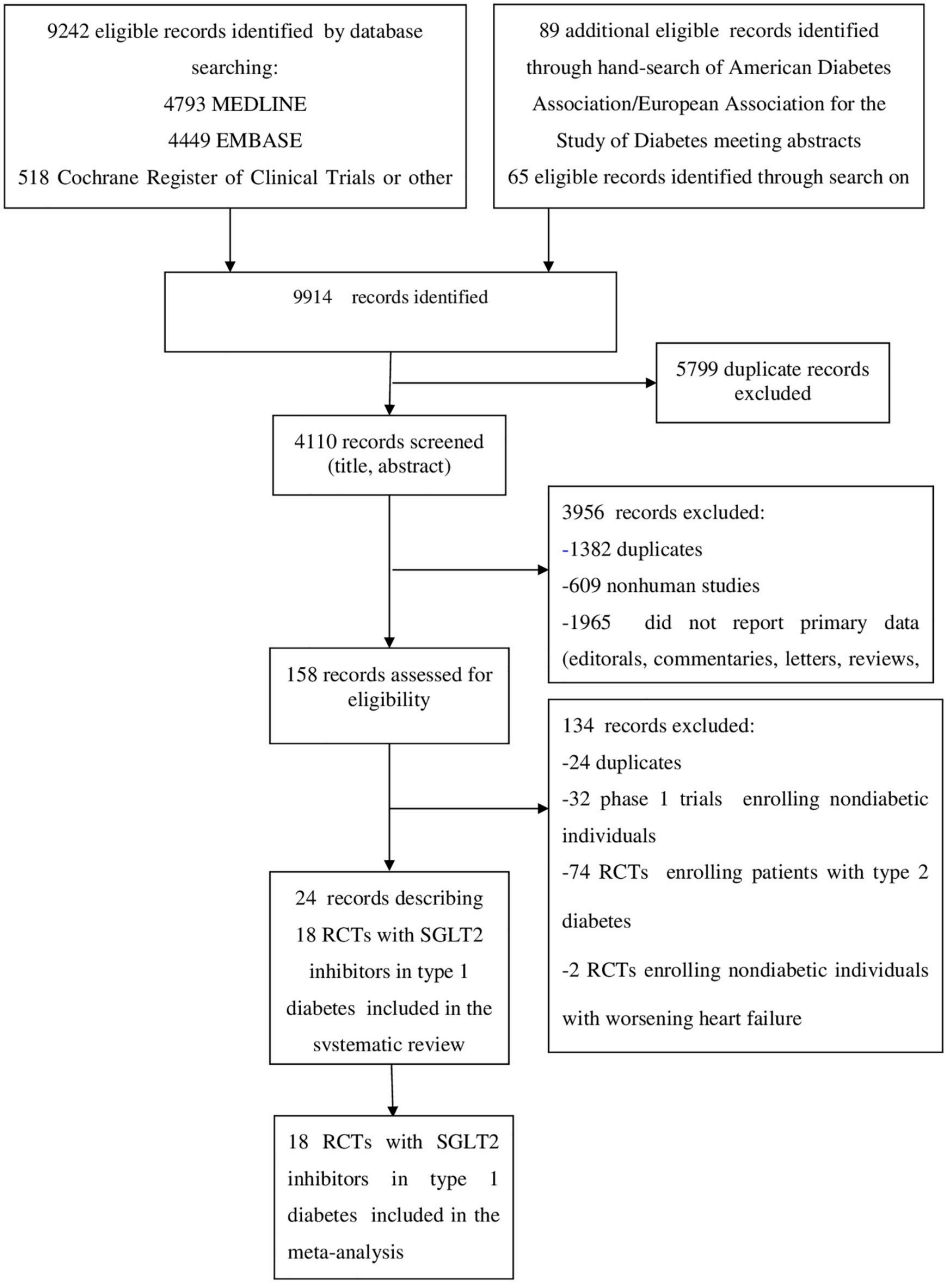
Evidence acquisition flow diagram.

The flow of study selection is reported in Fig 1. At the end of selection, 24 records describing 18 placebo-controlled RCTs [7,396 participants, 50% males, mean age 42 y (range 23 to 55 y) mean duration 19 weeks (range 1 to 52 weeks)] were included in the meta-analysis. Five RCTs evaluated dapagliflozin [52–57], 2 RCTs evaluated ipragliflozin [58,59], 1 RCT evaluated canagliflozin [60–62], 4 RCTs evaluated empagliflozin [63–66], and 6 RCTs evaluated sotagliflozin [67–74] (main trial characteristics reported in **Table A** in S1 Tables).

No ongoing or planned RCTs with SGLT2i in T1DM were detected.

All included RCTs compared SGLT2i with placebo on background insulin treatment.

Thirteen RCTs (5,673 participants) compared different SGLT2i doses with placebo. Overall, 38 comparisons were available for the meta-analysis.

Eight RCTs adopted insulin dose optimization prior to randomization [56,57,60,65,66,72,73].

Eleven RCTs excluded patients with impaired renal function (eGFR <60 ml/min/1.73 m2) [52,53,56–60,63,66,67,71], 7 RCTs excluded patients with severe (stage 4: eGFR <30 ml/min/1.73 m2) renal impairment [54,65,69,72–74].

Overall, the quality of included RCTs was good. One RCT [53] had high risk-of-bias in 4 domains and another RCT had a high risk of sponsorship bias [66] (**Table A** in S1 Tables).

The median (range) diabetes duration of participants was 19.4 y (11 to 25 y).

Participants’ baseline characteristics were equally balanced between the study arms, and in all RCTs, dropout rates were generally low and balanced across arms. No trial used the last-observation-carried-forward (LOCF) approach to impute missing observations, which were imputed as nonresponse for dichotomous outcomes; for continuous outcomes, mixed-effects model for repeated measures (MMRM) statistics based on the restricted maximum likelihood method for estimation was used.

The risk-of-bias summary for individual RCTs and the risk of bias graph for each item across included RCTs are detailed in **Table A** in S1 Tables and summarized in **Fig A and B in** S1 Figs.

The analysis of Funnel plots and the Egger test (*p* > 0.59 for all outcomes) did not find any evidence of small-study effects (**Fig C** in S1 Figs).

We had to impute no values for the meta-analysis.

The agreement between the 2 reviewers for study selection was 0.91 and for quality assessment of trials was 0.90.

### Diabetic ketoacidosis (DKA)

**Fig 2:**
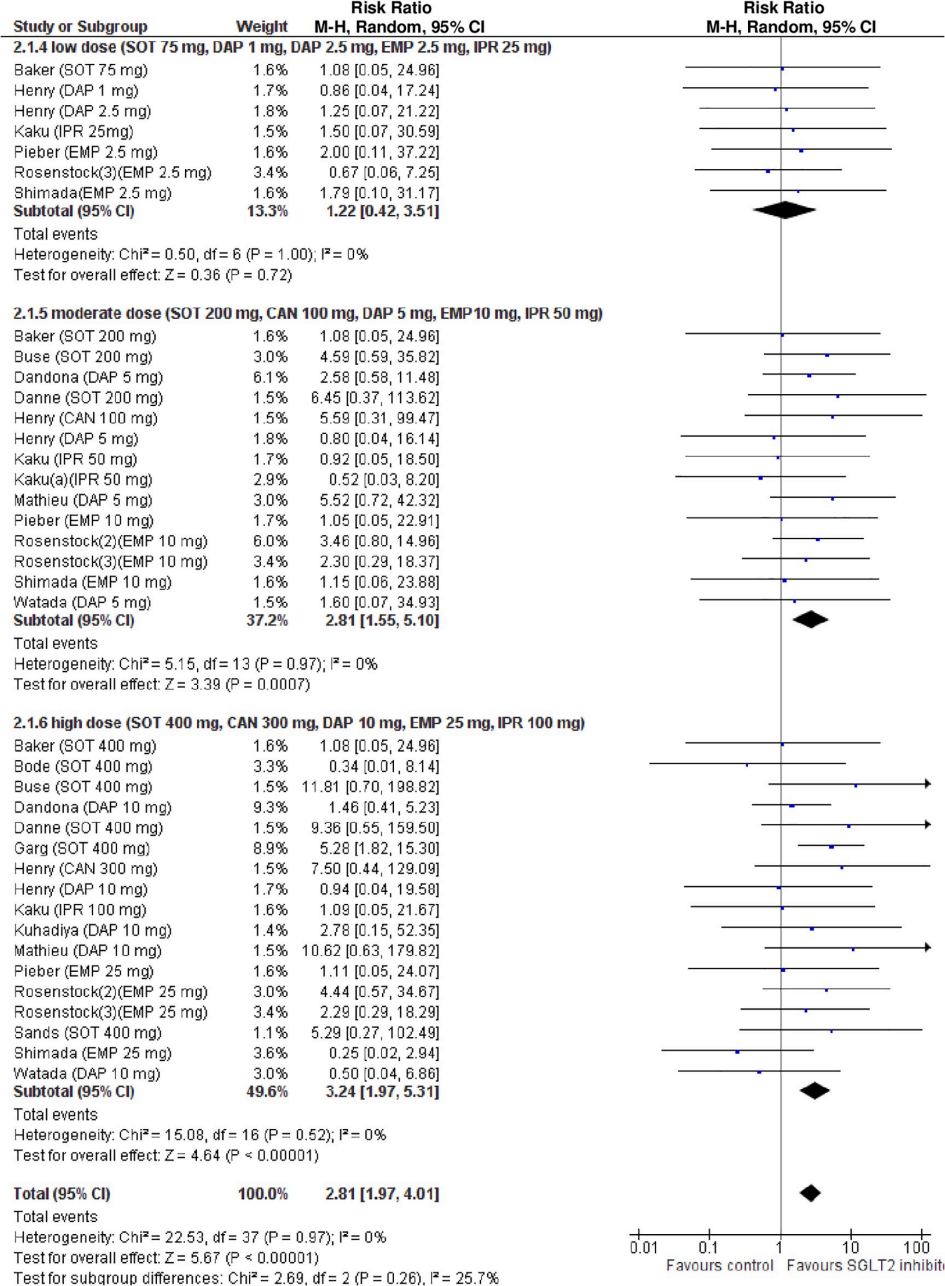
Forest plot of comparison: SGLT2 inhibitors, outcome: incident diabetic ketoacidosis (DKA).

The definition of DKA was consistent across all RCTs. Compared with placebo, SGLT2i were associated with an increased risk of DKA (RR 2.81, 95% CI: 1.97 to 4.01, *p* < 0.001; N comparisons = 38, I^2^ = 0%, 7,396 participants, trial duration ranging 1 to 52 weeks) (Fig 2). Individual effect estimates varied widely (range 0.34 to 11.81), confirming that I^2^ statistics has low power to detect heterogeneity in the presence of rare events and wide 95% CI and the appropriateness of meta-regression analysis [45].

Subgroup analysis revealed that the risk of DKA was increased in RCTs with a mean baseline BMI >27 kg/m^2^, and with a mean baseline eGDR <8.3 mg/kg/min, indicative of insulin resistance, but not in RCTs with a mean baseline BMI ≤27 kg/m2 or a baseline eGDR ≥8.3 mg/kg/min (**Table B** in S1 Tables).

**Fig 3:**
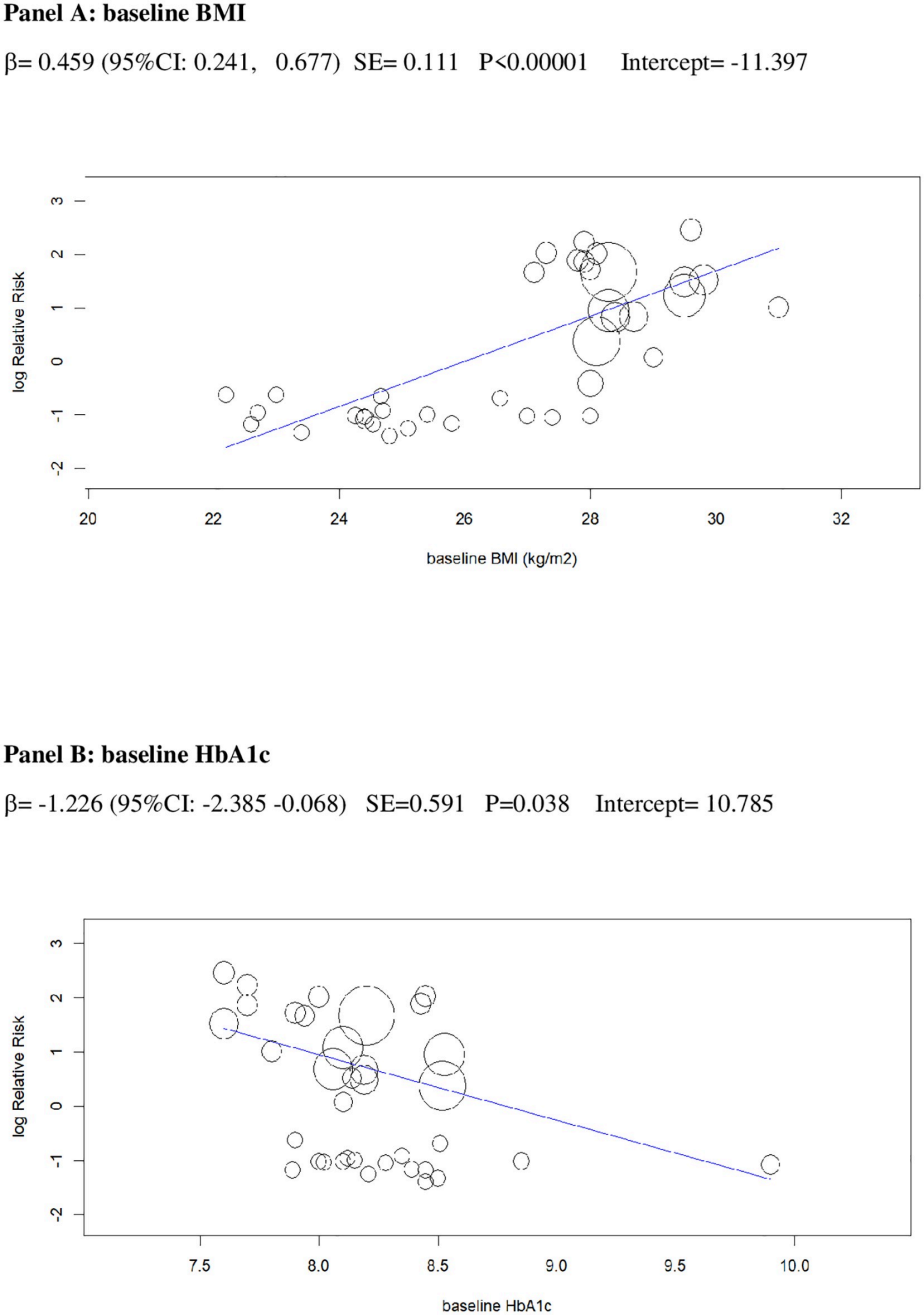
Univariable meta-regression plots for statistically significant moderators of the RR of DKA: Baseline BMI and baseline HbA1c. The radius of the points is proportional to the inverse of the SEs (i.e., larger/more precise studies are shown as larger points). BMI, body mass index; DKA, diabetic ketoacidosis; RR, risk ratio.

**Fig 4:**
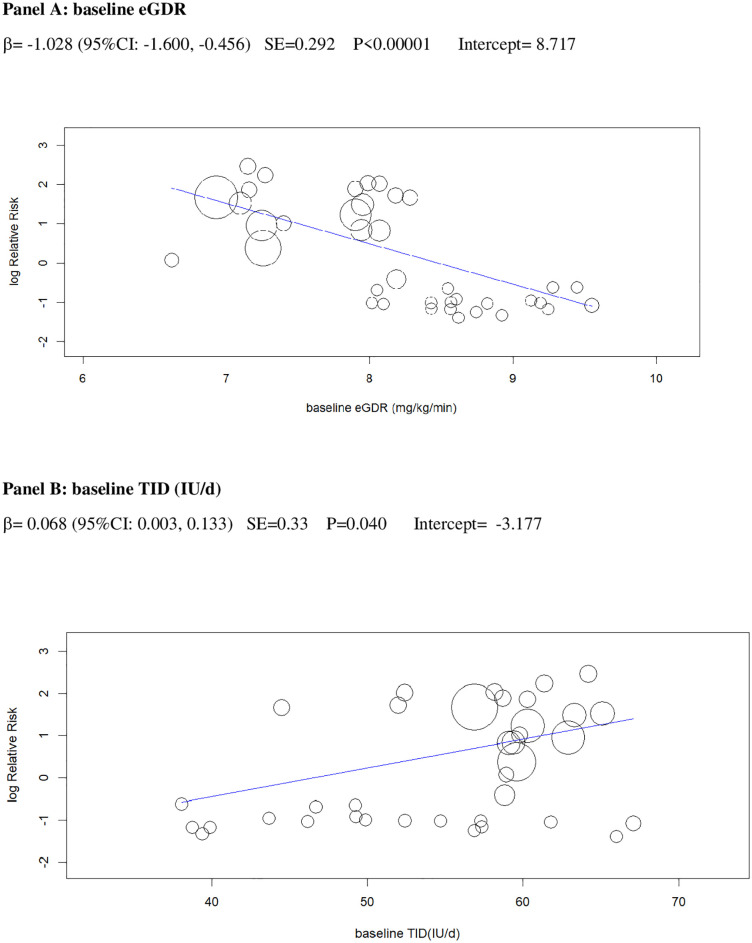
Univariable meta-regression plots for statistically significant moderators of the RR of DKA: Baseline eGDR and TID. DKA, diabetic ketoacidosis; eGDR, estimated glucose disposal rate; RR, risk ratio; TID, total insulin dose.

**Fig 5:**
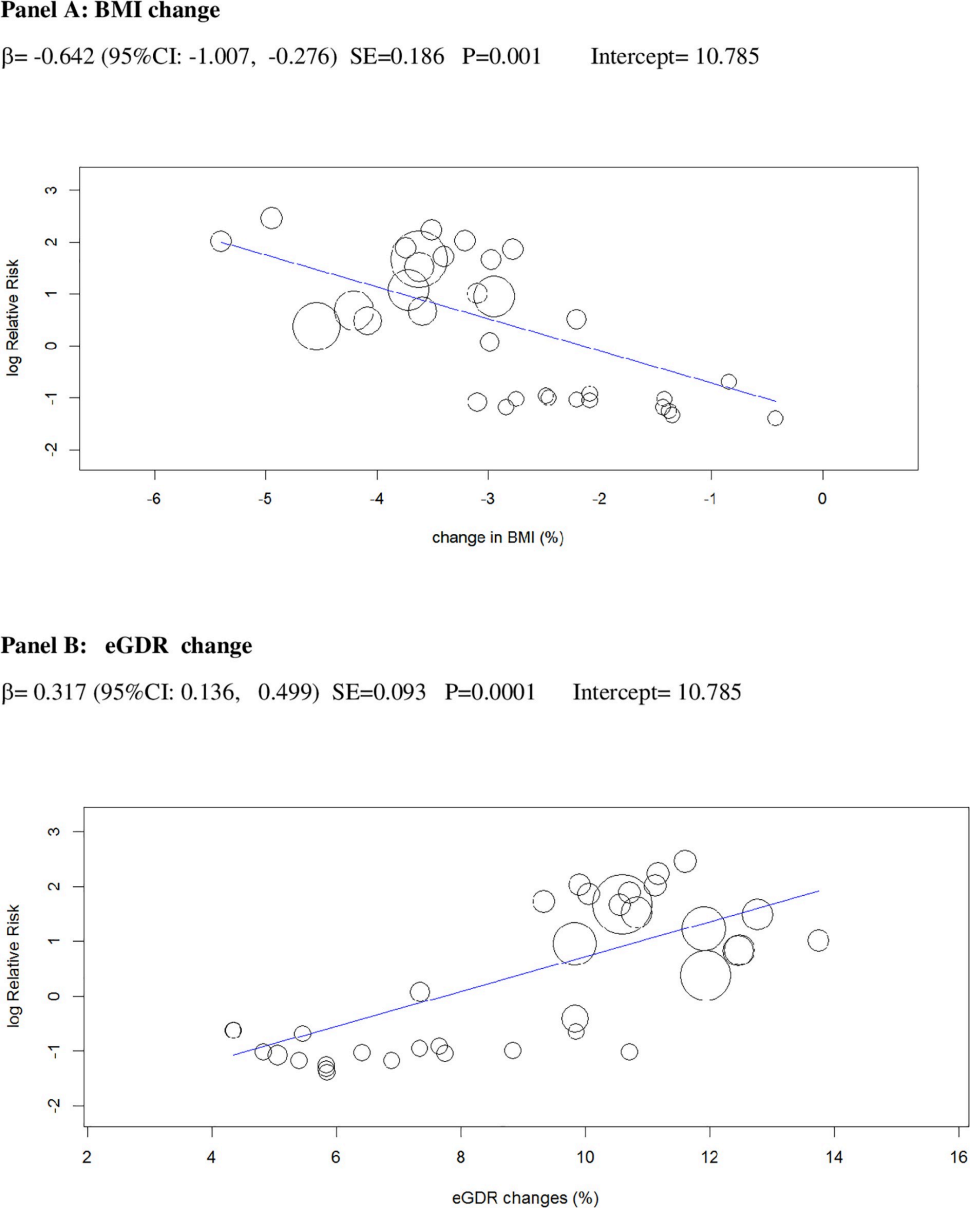
Univariable meta-regression plots for statistically significant moderators of the RR of DKA: Change in BMI and in eGDR. BMI, body mass index; DKA, diabetic ketoacidosis; eGDR, estimated glucose disposal rate; RR, risk ratio.

**Fig 6:**
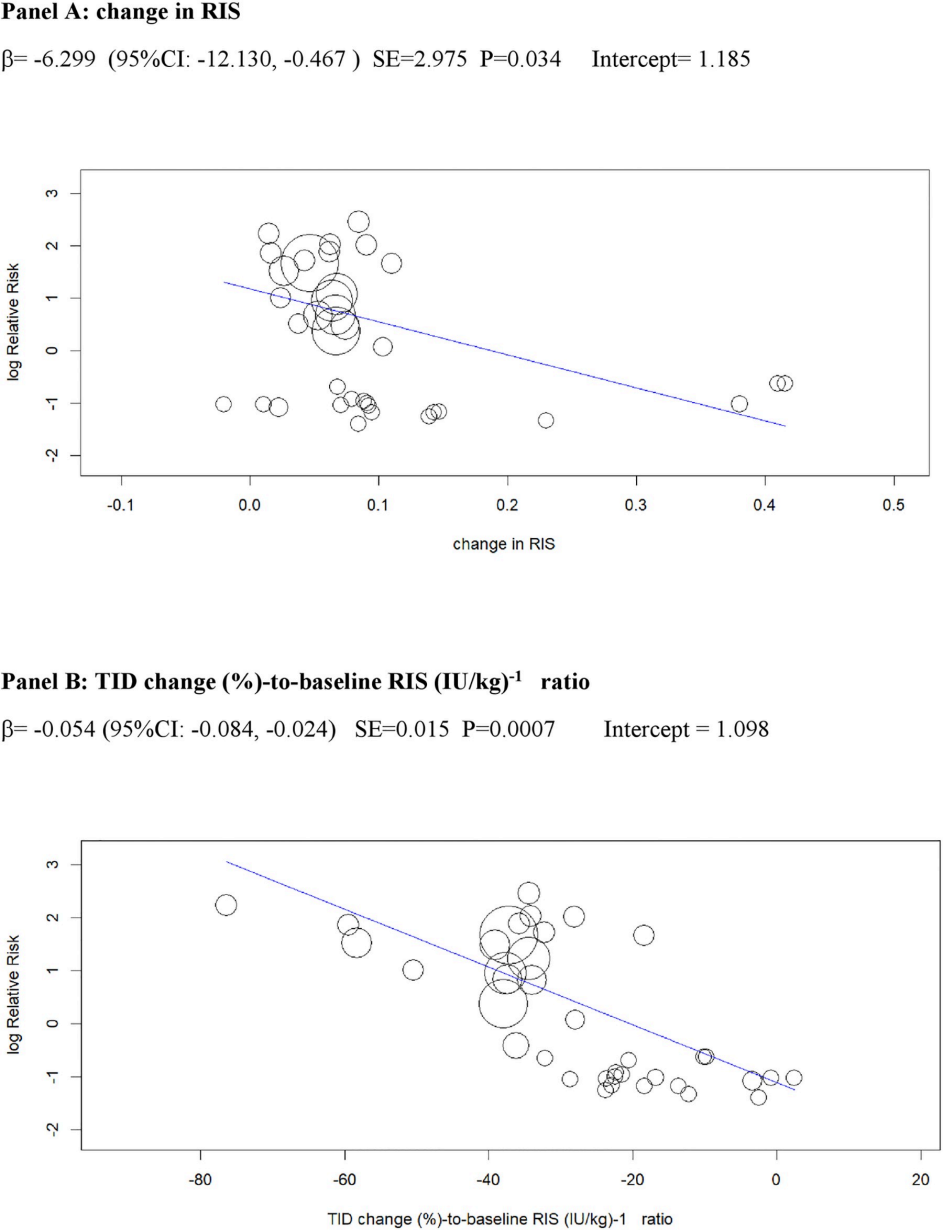
Univariable meta-regression plots for statistically significant moderators of the RR of DKA: Change in RIS and in TID (% change)-to-baseline RIS (IU/kg)^−1^ ratio. DKA, diabetic ketoacidosis; RIS, relative insulin; RR, risk ratio; TID, total insulin dose.

**Fig 7:**
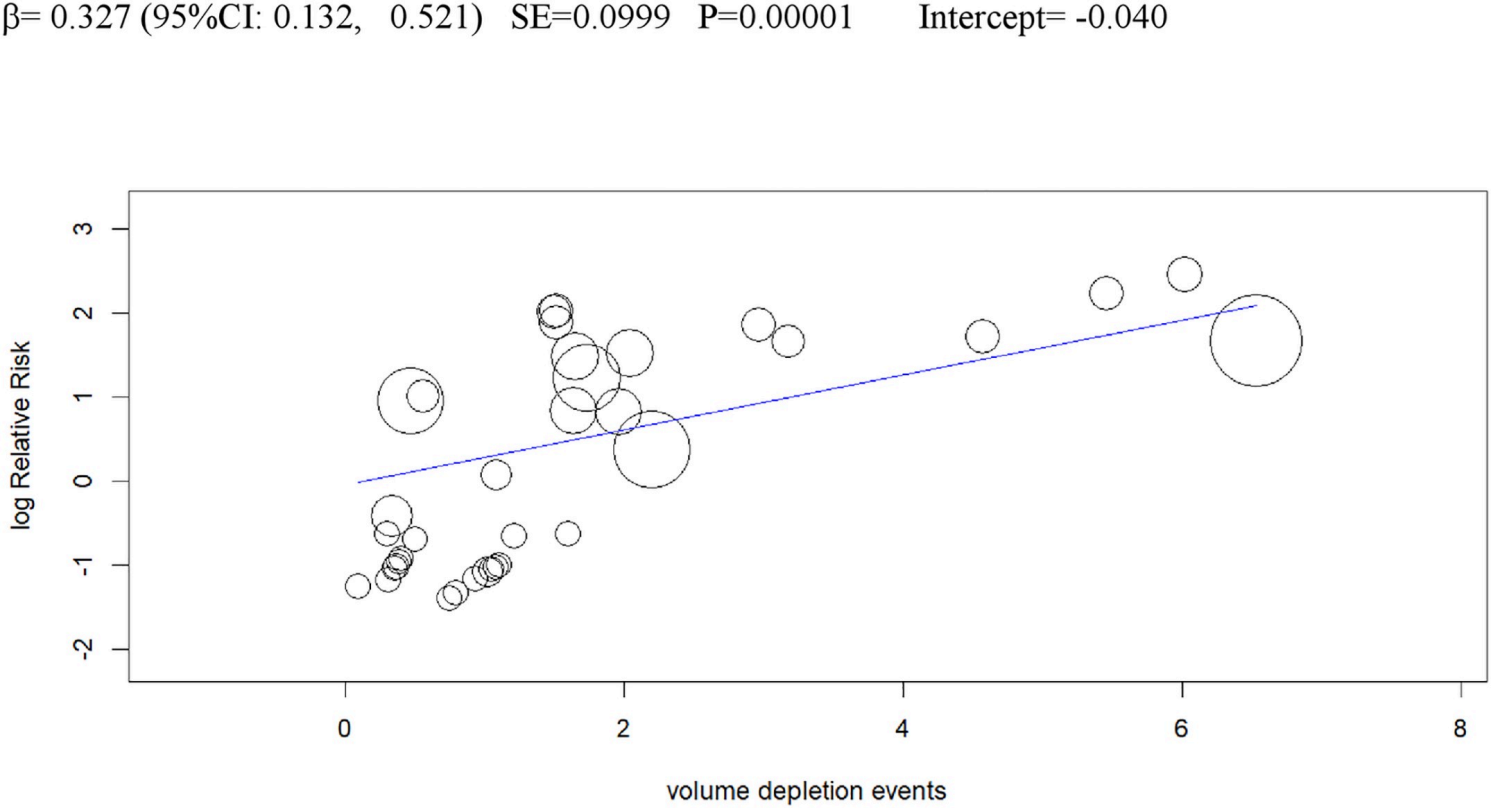
Univariable meta-regression plots for statistically significant moderators of the RR of DKA: Volume depletion events. DKA, diabetic ketoacidosis; RR, risk ratio.

In univariable analysis, 4 baseline parameters [BMI, HbA1c, eGDR, TID] and 5 treatment-related parameters [change in BMI, eGDR, and RIS, change in ratio of TID-to-baseline RIS and volume depletion events] were associated with the risk of DKA (Figs 3–7 **and Table C** in S1 Tables).

In a multivariable meta-regression model including only baseline parameters (Model 1), BMI and eGDR independently predicted incident DKA, explaining 61% of between-study variance in the RR of DKA(R^2^ = 61%, Table 2).

**Table 2:** Multivariable meta-regression models for moderatos of the relative risk of DKA. Only statistically significant moderators at univariable meta-regression were included in the models.

Diabetic ketoacidosis (DKA)														
Moderator	N comparisons	N participants	Multivariable model 1 (baseline predictors)				Multivariable model 2 (treatment-related predictors)				Multivariable model 3 (all predictors)			
			ß (95%CI)	SE	P	R^2^*	ß (95%CI)	SE	P	R^2^*	ß (95%CI)	SE	P	R^2^*
**Baseline BMI (kg/m2)**	**38**	**7,396**	0.439 (0.211, 0.666)	0.118	**0.0001**	**61%**	-	-	-	-	0.399 (0.121, 0.667)	0.136	**0.006**	**86%**
**Baseline HbA1c (%)**	**38**	**7,396**	−0.475 (−1.388, 0.437)	0.576	0.415		-	-	-	-	−0.309 (0.118, −0.736)	0.218	0.431	
**Baseline eGDR (mg/kg/min)**	**38**	**7,396**	−0.766 (−1.276, −0.256)	0.26	**0.001**		-	-	-	-	−0.631 (−1.243, −0.021)	0.312	**0.028**	
**Baseline TID (IU/d)**	**38**	**7,396**	0.049 (−0.021, 0.119	0.036	0.437		-	-	-	-	0.031 (−0.085, 0.147)	0.059	0.348	
**BMI change (%)**	**38**	**7,396**	-	-	-	-	−0.312 (−0.688, 0.064)	0.192	0.104	**37%**	−0.394 (−0.811, 0.023)	0.213	0.128	
**eGDR change (%)**	**38**	**7,396**	-	-	-	-	0.215 (−0.214, 0.530)	0.194	0.193		0.197 (−0.013, 0.407)	0.105	0.109	
**RIS change (I.U./kg)**^**−1**^	**38**	**7,396**	-	-	-	-	−4.385 (−1.744, −10.513	3.127	0.541		−2.180 (−9.991, 5.631)	3.985	0.713	
**TID change (%) (%)/baseline RIS ratio (IU**^**2**^**/kg/d)**	**38**	**7,396**	-	-	-	-	−0.048 (−0.057, −0.039)	0.004	**0.007**		−0.037 (−0.047, −0.027)	0.005	**0.01**	
**Volume depletion events**	**38**	**7,396**	-	-	-	-	0.352 (0.193, 0.475)	0.06	**0.009**		0.296 (0.098, 0.494)	0.101	**0.011**	

*R^2^ is the ratio of explained to total variance and indicates the proportion of variance accounted for by different moderators.

BMI, body mass index; DKA, diabetic ketoacidosis; eGDR, estimated glucose disposal rate; RIS, relative insulin sensitivity; TID, daily total insulin dose.

In a multivariable model including only treatment-related parameters (Model 2), the change in TID (%)-to-baseline RIS ratio and volume depletion events were independently associated with between-study variance in the RR of DKA (R^2^ = 39%, Table 2).

In a multivariable model (Model 3) including all (baseline and treatment-related) parameters associated with DKA at univariable analysis, 4 parameters [baseline BMI and eGDR, TID change (%)-to-baseline RIS ratio, and volume depletion events] were independently associated with between-study variance in the RR of DKA (R^2^ = 86%) (Table 2).

### Glycemic efficacy outcomes HbA1c

**Fig 8:**
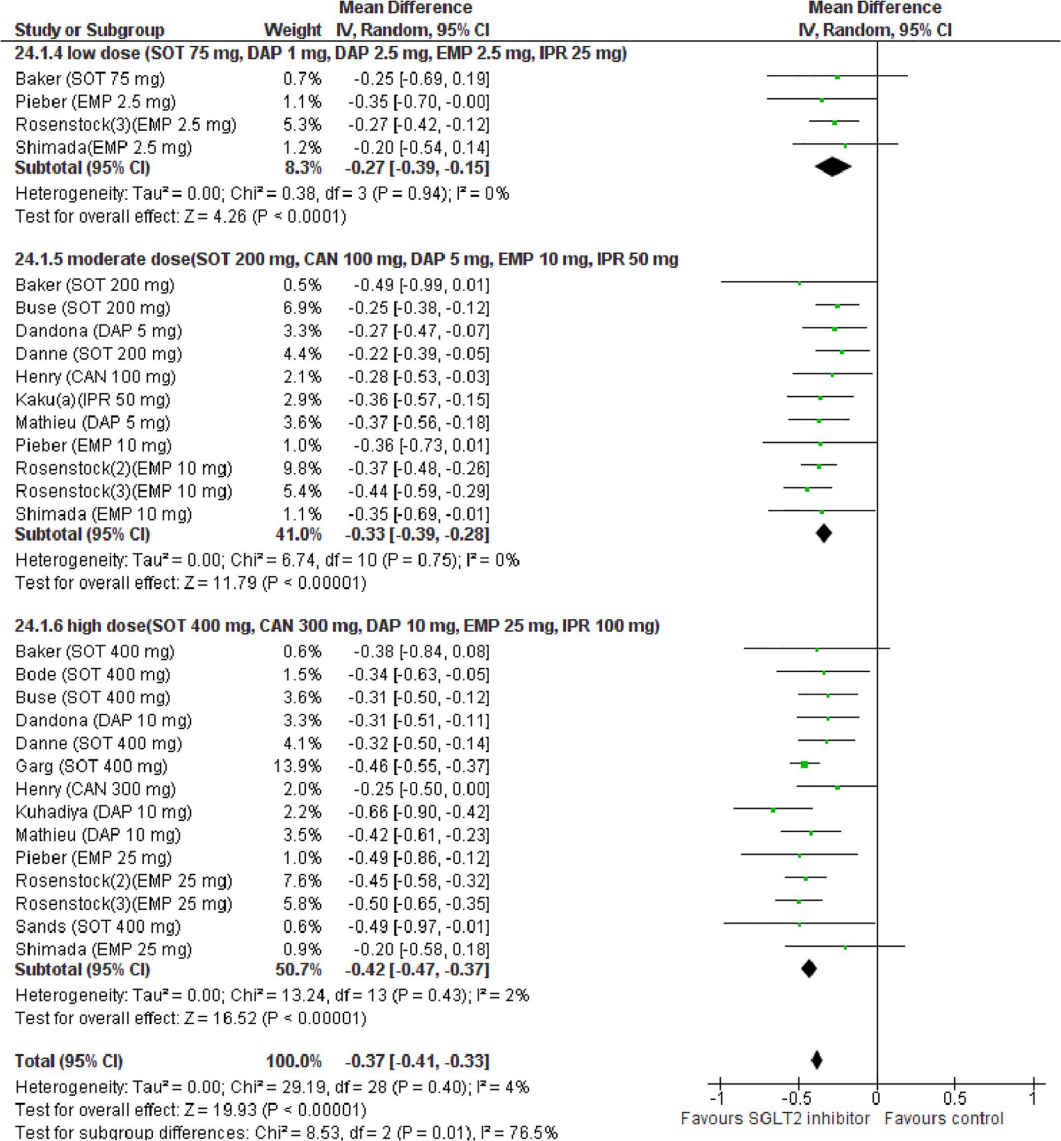
Forest plot of comparison: SGLT2 inhibitors, outcome: HbA1c change (%).

Compared with placebo, SGLT2i treatment reduced HbA1c (WMD −0.37%, 95% CI: −0.41% to −0.33%, *p* < 0.001, I^2^ = 4%, N-comparisons = 29, 7,243 participants) (Fig 8). Subgroup and univariable meta-regression analyses revealed that HbA1c change was associated with SGLT2i dose, pre-randomization insulin optimization and eGDR change, but not with baseline BMI or HbA1c or with treatment duration (**Table B and D** in S1 Tables). In a multivariable meta-regression model including baseline and treatment-related factors, only SGLT2i dose predicted HbA1c changes (R^2^ = 68%) (Table 3).

**Table 3:** Multivariable meta-regression models for moderatos of HbA1c and BMI changes. Only statistically significant moderators at univariable meta-regression were included in the models.

Moderator	N comparisons	N participants	Multivariable model 1 (baseline predictors)				Multivariable model 2 (treatment-related predictors)				Multivariable model 3 (all predictors)			
			ß (95%CI)	SE	P	R^2^*	ß (95%CI)	SE	P	R^2^*	ß (95%CI)	SE	P	R^2^*
**HbA1c (%)**														
**SGLT2 inhibitor dose**	**38**	**7,396**	−0.065 (−0.122, −0.009)	0.028	**0.005**	**56%**	**-**	**-**	**-**	**-**	−0.068 (−0.126, −0.010)	0.029	**0.009**	**68%**
**Pre-randomization insulin optimization**	**38**	**7,396**	0.050 (−0.039, 0.138)	0.045	0.269		**-**	**-**	**-**	**-**	0.083 (−0.011, 0.177)	0.048	0.085	
**Change in eGDR(%)**	**38**	**7,396**	**-**	**-**	**-**		−0.024 (−0.045, −0.002)	0.01	**0.031**	**28%**	−0.026 (−0.054, 0.001)	0.015	0.061	
**BMI (%)**														
**Total ID (IU/d)**	**38**	**7,396**	−0.004 (−0.075, −0.066)	0.036	0.904	**58%**	**-**	**-**	**-**	**-**	−0.005 (−0.069, −0.060)	0.033	0.889	**63%**
**BMI (kg/m**^**2**^**)**	**38**	**7,396**	−0.148 (−0.418, 0.123)	0.138	0.284		**-**	**-**	**-**	**-**	0.092 (−0.179, 0.362)	0.138	0.506	
**eGDR(mg/kg/min)**	**38**	**7,396**	−0.032 (−0.567, 0.503)	0.045	0.907		**-**	**-**	**-**	**-**	−0.216 (−0.693, −0.262)	0.244	0.376	
**SGLT2 inhibitor dose**	**38**	**7,396**	−0.773 (−1.177, −0.365)	0.205	**<0.0001**		**-**	**-**	**-**	**-**	−0.433 (−0.827, −0.039)	0.199	**0.002**	
**eGDR change (%)**	**38**	**7,396**	**-**	**-**	**-**	**-**	−0.331 (−0.453, −0.209)	0.062	**<0.0001**	**34%**	−0.326 (−0.488, −0.164)	0.083	**0.0008**	
**DKA**	**38**	**7,396**	**-**	**-**	**-**	**-**	−0.080 (−0.163, −0.004)	0.043	0.069		−0.071 (−0.167, −0.025)	0.049	0.147	

*R^2^ is the ratio of explained to total variance and indicates the proportion of variance accounted for by different moderators.

BMI, body mass index; DKA, diabetic ketoacidosis; eGDR, estimated glucose disposal rate; ID, daily insulin dose; RIS, relative insulin sensitivity; SGLT2, sodium-glucose cotransporter-2.

### Other glycemic efficacy outcomes

The results of meta-analysis for other glycemic efficacy outcomes are reported in Table 3 and in **Fig D–F in** S1 Figs: Compared with placebo, SGLT2i increased urinary glucose excretion (UGE) (g/d) and improved FPG (mg/dL), continuous glucose monitoring (CGM) parameters time-in-range (%) and MAGE (mg/dL), daily total/basal/bolus insulin dose, and insulin sensitivity indices.

### Non-glycemic outcomes

#### BMI

Compared with placebo, SGLT2i reduced BMI (WMD −3.20%, 95% CI: −3.54 to −2.86%, *p* < 0.001, I^2^ = 47%, N-comparisons = 39, 7,396 participants) (**Fig G** in S1 Figs).

Subgroup and univariable meta-regression analysis revealed that BMI change was associated with 4 baseline moderators (TID, BMI, eGDR, and SGLT2i dose) and with 2 treatment-related moderators (eGDR change and DKA) (**Table B and E** in S1 Tables).

In a multivariable meta-regression model including baseline and treatment-related factors (Model 3), SGLT2i dose and eGDR change were independently associated with BMI changes (R^2^ = 63%) (Table 3).

### Systolic blood pressure (sysBP)

Compared with placebo, SGLT2i reduced sysBP (WMD −3.81 mmHg, 95% CI: −4.49 to −3.12, *p* < 0.001, I^2^ = 0%, N-comparisons = 39, 7,396 participants) (Table 4 **and Fig G** in S1 Figs).

**Table 4:** Multivariable meta-regression models for moderatos of systolic BP, eGFR, and ACR. Only statistically significant moderators at univariable meta-regression were included in the models.

Systolic BP (mmHg)														
Moderator	N comparisons	N participants	Multivariable model 1 (baseline predictors)				Multivariable model 2 (treatment-related predictors)				Multivariable model 3 (all predictors)			
			ß (95%CI)	SE	P	R^2^*	ß (95%CI)	SE	P	R^2^*	ß (95%CI)	SE	P	R^2^*
**Baseline sys BP (mmHg)**	**38**	**7,396**	0.147 (−0.043, 0.336)	0.097	0.129	**62%**	**-**	**-**	**-**	**-**	0.147 (−0.043, 0.336)	0.097	0.129	**62%**
**SGLT2 inhibitor dose**	**38**	**7,396**	−1.349 (−2.490, −0.208)	0.581	**0.012**		**-**	**-**	**-**	**-**	−1.349 (−2.490, −0.208)	0.581	**0.012**	
**eGFR (ml/min/1.73 m**^**2**^**)**														
**Renal function stage**	**38**	**7,396**	−0.068 (−0.901, 0.766)	0.425	0.873	**51%**	-	-	-	-	−0.068 (−0.901, 0.766)	0.425	0.873	**51%**
**Study duration (wk)**	**38**	**7,396**	0.030 (0.002, 0.058)	0.011	**0.038**		-	-	-	-	0.030 (0.002, 0.058)	0.011	**0.038**	
**Albumin-to-creatinine ratio (ACR, mg/g)**														
**SGLT2 inhibitor dose**	**38**	**7,396**	−9.977 (−16.076, −3.878)	3.812	**0.004**	**56%**	-	-	-	-	−7.926 (−13.929, −1.923)	3.369	**0.01**	**69%**
**Time-in-range (%) change**	**31**	**3,050**	-	-	-		0.364 (−1.707, 2.434)	1.056	0.731	**41%**	0.260 (−1.911, 0.430)	1.107	0.815	
**MAGE (mg/dL) change**	**31**	**3,050**	-	-	-		1.151 (0.445, 1.857)	0.36	**0.009**		0.973 (0.344, 1.602)	0.321	**0.011**	

*R^2^ is the ratio of explained to total variance and indicates the proportion of variance accounted for by different moderators.

ACR, albumin-to-creatinine ratio; BP, blood pressure; eGFR, estimated glomerular filtration rate; MAGE, mean amplitude of glucose excursions; SGLT2, sodium-glucose cotransporter-2.

At univariable meta-regression, baseline sysBP and SGLT2i dose were associated with sysBP changes (**Table F** in S1 Tables). In a multivariable meta-regression model (Model 3), only SGLT2i dose was associated with sysBP changes (R^2^ = 62%) (Table 4).

### Renal effects: eGFR and urinary ACR

Compared with placebo, SGLT2i were associated with a slight reduction in eGFR (WMD: −0.78, 95% CI: −1.29 to −0.26 ml/min/1.73 m^2^, *p* = 0.003, I^2^ = 0%, N comparisons = 39, 7,396 participants) (Table 4 **and Fig H** in S1 Figs). Subgroup analysis revealed that the eGFR reduction was observed only in RCTs lasting <24 weeks, but not in RCTs of longer duration (**Table B** in S1 Tables). Univariable and multivariable meta-regression analysis confirmed study duration was the only moderator of eGFR changes (**Table G** in S1 Tables **and** Table 4).

Urinary ACR was evaluated in 4 RCTs (trial duration ranging 4 to 52 weeks, mean baseline ACR in the microalbuminuric range). Pooled analysis of these RCTs showed SGLT2i treatment decreased ACR (WMD: −9.91, 95% CI: −16.26 to −3.55 mg/g, *p* = 0.002, I^2^ = 0%, N comparisons = 8, 3,052 participants (Table 4 **and Fig H** in S1 Figs).

On meta-regression analysis, SGLT2i dose and MAGE were independently associated with ACR change (Table 4 **and Table H** in S1 Tables).

### Diabetic eye disorders

In the pooled dataset, 14 cases of diabetic eye disorders occurred: 11 incident cases of hemorrhagic retinopathy, 1 case of macular edema, 1 case of glaucoma, and 1 case of vision loss).

Compared with placebo, SGLT2i were associated with a 73% lower risk of eye-related disorders (RR 0.27, 95% CI: 0.11 to 0.67, *p* = 0.005; I^2^ = 0%, N comparisons = 39, 7,396 participants) (**Fig I** in S1 Figs).

The effect was accounted for by a lower incidence of hemorrhagic retinopathy (RR 0.27, 95% CI: 0.10 to 0.72, *p* = 0.009; N comparisons = 38, I^2^ = 0%, 7,396 participants). Subgroup analysis revealed that the effect was statistically significant only in RCTs of duration ≥24 weeks (**Table B** in S1 Tables).

On meta-regression analysis, changes in time-in-range (%) were independent RRs for diabetic eye disorders (Table 5 **and Table I** in S1 Tables).

**Table 5:** Multivariable meta-regression models for moderatos of diabetic eye disorders, genital tract infections, and volume depletion events. Only statistically significant moderators at univariable meta-regression were included in the models.

Diabetic eye disorders														
Moderator	N comparisons	N participants	Multivariable model 1 (baseline predictors)				Multivariable model 2 (treatment-related predictors)				Multivariable model 3 (all predictors)			
			ß (95%CI)	SE	P	R^2^*	ß (95%CI)	SE	P	R^2^*	ß (95%CI)	SE	P	R^2^*
**SGLT2 inhibitor dose**	**38**	**7,396**	−0.742 (−1.443, −0.041)	0.317	**0.031**	**49%**	-	-	-	66%	−0.555 (−1.903, 0.793)	0.688	0.587	**64%**
**Time-in-range(%) change**	**31**	**3,050**	-	-	-		−0.117 (−0.211, −0.004)	0.049	**0.009**		−0.111 (−0.201, −0.014)	0.037	**0.01**	
**Genital tract infections (GTIs)**														
**Total ID (IU/d)**	**38**	**7,396**	0.047 (0.040, 0.901)	0.022	**0.011**	**32%**	-	-	-	-	0.042 (0.002, 0.080)	0.02	**0.043**	**61%**
**BMI (kg/m**^**2**^**)**	**38**	**7,396**	0.089 (−0.172, 0.349)	0.133	0.504		-	-	-	-	−0.134 (−0.553, 0.285)	0.214	0.531	
**eGDR (mg/kg/min)**	**38**	**7,396**	−0.237 (−0.710, 0.235)	0.241	0.325		-	-	-	-	−0.237 (−0.810, 0.336)	0.292	0.417	
**FPG change (mg/dL)**	**38**	**7,396**	-	-	-	-	0.032 (0.005, 0.060)	0.014	**0.012**	39%	0.030 (0.002, 0.058)	0.014	**0.039**	
**BMI change (%)**	**38**	**7,396**	-	-	-	-	−0.274 (−0.676, 0.129)	0.205	0.183		−0.292 (−0.720, 0.136)	0.218	0.181	
**eGDR change (%)**	**38**	**7,396**	-	-	-	-	0.044 (−0.169, 0.258)	0.109	0.683		0.041 (−0.221, 0.304)	0.134	0.758	
**Volume depletion events**														
**Baseline eGDR (mg/kg/min)**	**38**	**7,396**	−0.698 (−1.250, −0.145)	0.279	0.009	42%	-	-	-		−0.645 (−1.272, −0.019)	0.32	**0.014**	**59%**
**eGDR change (%)**	**38**	**7,396**	-	-	-	-	0.097 (0.055, 0.181)	0.043	0.021	31%	0.054 (−0.126 0.233)	0.092	0.56	
**DKA**	**38**	**7,396**	-	-	-	-	−0.068 (−0.091, −0.228)	0.081	0.399		−0.005 (−0.182, 0.173)	0.09	0.958	

*R^2^ is the ratio of explained to total variance and indicates the proportion of variance accounted for by different moderators.

BMI, body mass index; DKA, diabetic ketoacidosis; eGDR, estimated glucose disposal rate; FPG, fasting plasma glucose; ID, insulin doses.

### Safety outcomes other than DKA

The effect of SGLT2i on all AEs is summarized in **Table J** in S1 Tables.

The definition of hypoglycemia and severe hypoglycemia was consistent across all RCTs. Compared with placebo, SGLT2i did not affect the risk of hypoglycemia, severe hypoglycemia, UTIs, MACE, cancer, and all-cause death (**Fig I** in S1 Figs).

Compared with placebo, SGLT2i increased the risk of GTIs (RR 3.18, 95% CI: 2.49 to 4.06, *p* < 0.001; I^2^ = 0%, N-comparisons = 39, 7,396 participants) (**Fig I** in S1 Figs).

In a multivariable meta-regression model, the risk of GTI was independently associated with baseline TID and by changes in FPG (Table 5 **and Table L** in S1 Tables).

SGLT2i treatment was also associated with an increased risk of volume depletion events (RR: 1.53, 95% CI: 1.03 to 2.28, *p* = 0.03; I^2^ = 0%, N comparisons = 39, 7,396 participants) (**Fig I** in S1 Figs).

Subgroup analysis revealed that the risk of volume depletion increased in RCTs with a mean baseline eGDR <8.3 mg/kg/min, indicative of insulin resistance, but not in RCTs with a mean baseline eGDR ≥8.3 mg/kg/min (**Table B** in S1 Tables).

On meta-regression analysis, baseline eGDR and changes in eGDR were independently associated with RR of volume depletion events (Table 5 **and Table M** in S1 Tables).

The results of meta-analysis for glycemic and non-glycemic efficacy outcomes are summarized in Table 6.

**Table 6:** Summary of main findings of meta-analysis for glycemic and non-glycemic efficacy outcomes.

**Glycemic efficacy outcomes**					
**Outcome**	**Comparisons (n)**	**Participants (n)**	**Effect estimate [95% CI]**	***P***	**I^2^ (%)**
**HbA1c (%)**	**29**	**7,243**	**WMD: −0.37 (−0.41, −0.33)**	**<0.001**	**4**
**Fasting plasma glucose (FPG)**	**39**	**7,396**	**WMD: −19.20 (−22.28, −16.12)**	**<0.001**	**0**
**Time-in-range (%)**	**31**	**3,050**	**WMD: +9.87 (+8.75, +10.99)**	**<0.001**	**10**
**MAGE (mg/dL)**	**38**	**3,050**	**WMD: −15.91 (−17.95, −13.86)**	**<0.001**	**0**
**Daily TID (%)**	**39**	**7,396**	**WMD: −10.60 (−11.72, −9.47)**	**<0.001**	**17**
**Daily basal ID (%)**	**39**	**7,396**	**WMD: −12.37% (−14.15, −10.59)**	**<0.001**	**38**
**Daily bolus ID (%)**	**39**	**7,396**	**WMD: −9.81% (−11.45, −8.18)**	**<0.001**	**18**
**Estimated glucose disposal rate (eGDR) (%)**	**39**	**7,396**	**WMD: +11.06% (+10.16, +11.96)**	**<0.001**	**33**
**Relative insulin sensitivity (RIS) (%)**	**39**	**7,396**	**WMD: +10.44% (+9.49, +11.39)**	**<0.001**	**48**
**Non-glycemic efficacy outcomes**					
**Outcome**	**Comparisons (n)**	**Participants (n)**	**Effect estimate** [**95% CI**]	***P***	**I**^**2**^ (**%)**
**BMI (%)**	**39**	**7,396**	**WMD: −3.20% (−3.54, −2.86)**	**<0.001**	**47**
**SysBP (mmHg)**	**39**	**7,396**	**WMD: −3.81 mmHg (−4.49, −3.12)**	**<0.001**	**0**
**eGFR (ml/min/1.73 m**^**2**^**)**	**39**	**7,396**	**WMD: −0.78 (−1.29, −0.26)**	**0.003**	**0**
**ACR (mg/g)**	**8**	**3,052**	**WMD: −9.91 (−16.26, −3.55)**	**0.002**	**0**
**Diabetic eye disorders**	**39**	**7,396**	**RR: 0.38 (0.10, 1.40)**	**0.005**	**0**

ACR, albumin-to-creatinine ratio; eGFR, estimated glomerular filtration rate; ID, insulin dose; MAGE, mean amplitude of glucose excursions; sysBP, systolic blood pressure; TID, total daily insulin dose; WMD, weighted mean difference.

### Dose-response analysis

We analyzed dose-response interactions within the 13 RCTs (5,673 participants) that evaluated different SGLT2i doses: A significant dose-response gradient for low doses versus moderate doses versus high doses was noted for HbA1c (%), FPG (mg/dL), time-in-range (%), total/basal/bolus insulin dose (%), eGDR (%), RIS, BMI (%), sysBP (mmHg), and urinary ACR (mg/g) (Table 7).

**Table 7:** Dose-response interaction: Within-trial analysis of the pooled data from RCTs evaluating multiple SGLT2 inhibitor doses.

Outcome	SGLT2 inhibitor high dose vs. moderate dose	SGLT2 inhibitor high dose vs. low dose	SGLT2 inhibitor moderate dose vs. low dose
**DKA**	1.00 (0.70, 1.45) I^2^ = 0%, *p* = 0.98, *N* = 13, 3,577 participants	2.55 (0.60, 10.88) I^2^ = 0%, *p* = 0.30, *N* = 7, 675 participants	2.91 (0.59, 14.29) I^2^ = 0%, *p* = 0.29, *N* = 7, 675 participants
**HbA1c (%)**	−0.08 (−0.15, −0.01) I^2^ = 0%, *p* = 0.008, *N* = 10, 3,498 participants	−0.17 (−0.27, −0.07), I^2^ = 1 2%, *p* = 0.0006, *N* = 4, 634 participants	−0.16 (−0.29, −0.06), I^2^ = 0%, *p* = 0.01, *N* = 4, 634 participants
**FPG (mg/dL)**	−7.59 (−12.38, −2.80), I^2^ = 25, *p* = 0.01, *N* = 13, 3,577 participants	−24.60 (−38.91, −10.28), I^2^ = 23%, *p* = 0.00008, *N* = 7, 675 participants	−7.51 (−15.16, −1.15), I^2^ = 0%, *p* = 0.02, *N* = 7, 675 participants
**Time-in-range (%)**	2.05 (0.33, 3.78), I^2^ = 0%, *p* = 0.01, *N* = 11, 1,821 participants	6.07 (3.28, 8.85), I^2^ = 0%, *p* < 0.0001, *N* = 6, 498 participants	4.80 (1.33, 8.27), I^2^ = 0%, *p* = 0.007, *N* = 6, 214 participants
**MAGE (mg/dL)**	−2.14 (−5.81, 1.54), I^2^ = 28%, *p* = 0.25, *N* = 11, 1,821 participants	−2.95 (−9.99, 4.09), I^2^ = 26%, *p* = 0.41, *N* = 6, 498 participants	−4.76 (−18.45, 8.94), I^2^ = 32%, *p* = 0.50, *N* = 6, 214 participants
**Total insulin dose (%)**	−3.14 (−6.78, 11.98), I^2^ = 0%, *p* = 0.0003, *N* = 13, 3,577 participants	−7.01 (−9.76, −4.53), I^2^ = 0%, *p* < 0.00001, *N* = 7, 677 participants	−2.52 (−4.99, −0.16), I^2^ = 0%, *p* = 0.04, *N* = 7, 677 participants
**Basal insulin dose (%)**	−3.33 (−4.85, −1.83), I^2^ = 1%, *p* = 0.0001, *N* = 13, 3,577 participants	−4.61 (−8.04, −1.34), I^2^ = 0%, *p* = 0.01, *N* = 7, 677 participants	−1.23 (−4.39, 1.93), I^2^ = 0%, *p* = 0.45, *N* = 7, 677 participants
**Bolus insulin dose (%)**	−4.58 (−8.06, −1.10), I^2^ = 30%, *p* = 0.01, *N* = 13, 3,577 participants	−7.35(−11.56, −3.03), I^2^ = 0%, *p* = 0.001, *N* = 7, 677 participants	−3.55(−6.93, −0.35), I^2^ = 0%, *p* = 0.04, *N* = 7, 677 participants
**eGDR (mg/kg/min)**	2.34 (1.36, 3.03), I^2^ = 0, *p* < 0.00001, *N* = 13, 3,577 participants	5.85 (1.63, 9.67), I^2^ = 30%, *p* = 0.001, *N* = 7, 677 participants	3.75 (0.95, 6.54), I^2^ = 35%, *p* = 0.009, *N* = 7, 677 participants
**RIS**	1.14 (0.34, 1.71), I^2^ = 0, *p* = 0.004, *N* = 13, 3,577 participants	3.81 (1.48, 5.62), I^2^ = 38%, *p* = 0.003, *N* = 7, 677 participants	3.35 (1.47, 5.24), I^2^ = 23%, *p* = 0.0005, *N* = 7, 677 participants
**BMI (%)**	−0.89 (−1.20, −0.53), *p* < 0.0001, *N* = 13, 3,577participants	−1.00 (−1.87, −0.23), I^2^ = 0%, *p* = 0.01, *N* = 7, 677 participants	−0.84 (−1.38, −0.30), I^2^ = 10%, *p* = 0.002, *N* = 7, 677 participants
**Systolic BP (mmHg)**	−1.29 (−2.19, −0.19), *p* = 0.03, *N* = 13, 3,577 participants	−2.82 (−4.85, −1.21), I^2^ = 0%, *p* = 0.02, *N* = 7, 677 participants	−1.76 (−4.37, −0.86), I^2^ = 21%, *p* = 0.04, *N* = 7, 677 participants
**eGFR (ml/min/1.73 m**^**2**^**)**	0.18 (−0.46, 0.82), *p* = 0.40, *N* = 13, 3,577 participants	−0.13 (−1.50, 1.85), *p* = 0.85, *N* = 7, 677 participants	−0.35 (−1.80, 1.10, *p* = 0.64, *N* = 7, I^2^ = 0%, 677 participants
**Urinary ACR (mg/g)**	−6.20 (−10.59, −0.08), I^2^ = 0%, *p* = 0.04, *N* = 4, 1,086 participants	−7.20 (−14.59, −0.08), I^2^ = NA, *p* = 0.04, *N* = 1, 38 participants	−7.40 (−15.32, −0.52), I^2^ = NA, *p* = 0.04, *N* = 1, 38 participants
**Eye disorders**	0.25 (0.03, 2.21), *p* = 0.21, *N* = 13, 3,577 participants	0.36 (0.04, 3.24), I^2^ = 0%, *p* = 0.36, *N* = 6, 677 participants	0.36 (0.02, 8.05), I^2^ = 0%, *p* = 0.52, *N* = 6, 677 participants
**Hypoglycemia**	0.98 (0.85, 1.15), *p* = 0.71, *N* = 13, 3,577 participants	1.00 (0.94, 1.06), I^2^ = 0%, *p* = 0.98, *N* = 6, 677 participants	0.99 (0.93, 1.05), I^2^ = 0%, *p* = 0.92, *N* = 6, 677 participants
**Severe hypoglycemia**	0.47 (0.13, 1.79), *p* = 0.31, *N* = 13, 3,577 participants	0.76 (0.45, 1.24), I^2^ = 0%, *p* = 0.29, *N* = 6, 677 participants	0.80 (0.55, 1.05), I^2^ = 0%, *p* = 0.10, *N* = 6, 677 participants
**UTI**	0.68 (0.30, 1.78), *p* = 0.41, *N* = 13, 3,577 participants	1.22 (0.92, 1.64), I^2^ = 0%, *p* = 0.89, *N* = 6, 677 participants	0.89 (0.84, 1.25), I^2^ = 0%, *p* = 0.39, *N* = 6, 677 participants
**GTI**	1.11 (0.89, 1.33), *p* = 0.34, *N* = 13, 3,577 participants	1.64 (0.90, 3.01), I^2^ = 0%, *p* = 0.19, *N* = 6, 677 participants	1.46 (0.79, 1.72), I^2^ = 0%, *p* = 0.23, *N* = 6, 677 participants
**Volume depletion events**	0.94 (0.58, 1.54), *p* = 0.82, *N* = 13, 3,577 participants	2.17 (0.62, 7.53), I^2^ = 0%, *p* = 0.23, *N* = 6, 677 participants	2.54 (0.69, 9.33), I^2^ = 0%, *p* = 0.16, *N* = 6, 677 participants
**MACE**	1.08 (0.18, 1.98), *p* = 0.82, *N* = 13, 3,577 participants	0.92 (0.37, 2.32), I^2^ = 0%, *p* = 0.39, *N* = 6, 677 participants	1.18 (0.40, 3.39), I^2^ = 0%, *p* = 0.86, *N* = 6, 677 participants

ACR, albumin-to-creatinine ratio; DKA, diabetic ketoacidosis; eGDR, estimated glucose disposal rate; eGFR, estimated glomerular filtration rate; FPG, fasting plasma glucose; GTI, genital tract infections; MACE, major adverse cardiovascular events; MAGE, major amplitude of glucose excursions; RIS, relative insulin sensitivity; UTI, urinary tract infections.

We did not find any relationship between different SGLT2i doses and AEs. The results of the within-trial comparison were all confirmed by the across-trial approach.

### Analysis of individual SGLT2i

We noted no clear evidence that individual drugs had different effects on DKA and on other efficacy and safety outcomes (all I^2^ ≤20%). However, sotagliflozin slightly reduced the risk of severe hypoglycemia, as previously reported [13] (**Table N** in S1 Tables).

### Sensitivity analyses

Sensitivity analysis conducted by excluding RCTs at high risk of bias in any domain, by repeating meta-analysis and meta-regression using alternative effect measures, pooling methods, statistical models, by using 1-step fully adjusted multivariable models and leave-one-out meta-analysis, confirmed robustness of the main analysis (**Table O–Q** in S1 Tables, **Fig L** in S1 Figs).

### Grading of evidence

Quality of evidence was downgraded to moderate for MACE for imprecision (**Table R** in S1 Tables).

## Discussion

The main findings of our meta-analysis and meta-regression of RCTs evaluating SGLT2i in T1DM are the following: First, in multivariable meta-regression, baseline BMI and insulin resistance were independently associated with the risk of DKA, explaining 61% of variance across studies in the RR of DKA. A model including 2 additional parameters (ratio of TID change-to-insulin sensitivity and volume depletion) explained 86% of variance in DKA risk. These findings were confirmed by results of subgroups analysis.

Second, moderators of DKA risk differed substantially from those of efficacy outcomes, indicating a selection of T1DM patients with the highest benefit/risk ratio with SGLT2i is feasible.

Third, a consistent dose-response gradient with increasing SGLT2i doses was observed for major efficacy outcomes, but not for DKA and other AEs.

Fourth, among non-glycemic benefits, we disclosed signals for renal and eye protection associated with SGLT2i treatment.

Patients with T1DM need adjunctive therapies to improve glycemic control and mitigate unwanted effects of insulin [1,2,3]. SGLT2i confer extensive glycemic and non-glycemic benefits, which, however, must be weighed against the risk of DKA in T1DM.

To date, there is no evidence-based precision medicine strategy to predict SGLT2i-associated therapeutic responses to SGLT2i, minimize DKA risk, and help select patients with the greatest benefit-to-risk ratio from these drugs. We therefore performed meta-analysis and multivariable meta-regression to disclose independent study-level moderators of risk of DKA and of main efficacy and safety outcomes in patients with T1DM treated with SGLT2i.

We found that 4 independent study-level moderators explained 86% of the variance among studies in DKA risk (Table 2).

The 2 baseline moderators, BMI and eGDR, together explained 61% of the across-study variance in DKA risk (Table 2): Patients with overweight and with insulin resistance may be at increased risk of DKA because they are more prone to unrestricted FFA lipolysis from their increased triglyceride stores during the negative glucose balance and insulin dose down-titration induced by SGLT2i [75]. Notably, the analysis of the regression plot (Fig 3) indicates that the DKA risk starts to increase with BMI ≥27.00 kg/m^2^, which coincides with the cut-off of approval for SGLT2i in Europe [11,12].

The 2 independent treatment-related moderators were the ratio of TID reduction (%)-to-baseline insulin sensitivity and volume depletion events, which together explained 37% of across-study variance in DKA risk.

Excessive insulin dose reduction plays a key role in DKA by enhancing lipolysis and ketogenesis [75], but the exact cut-off of insulin down-titration which augments DKA risk is unclear: We could not confirm the cut-offs suggested by experts (20% of initial TID or 10% of initial basal ID) [18], which were derived from a post hoc analysis of a small phase 2 RCT [47]. Rather, we found that the risk of DKA during insulin dose down-titration was related to individual insulin sensitivity at baseline: The higher insulin resistance, the more cautious TID reduction should be to prevent unrestricted lipolysis and ketogenesis. More specifically, the analysis of the regression plot (Fig 6) indicates that DKA risk starts to increase when the ratio of TID change (%)-to-baseline IS falls below −20.

Volume depletion was the fourth independent predictor of DKA, consistent with recent experimental evidence demonstrating dehydration and insulinopenia are both necessary and sufficient to trigger SGLT2i-associated DKA, through hypothalamic–pituitary–adrenal axis activation, catecholamine and corticosterone axis stimulation, and increased adipose tissue lipolysis [76]. Hence, dehydration would be a central target for DKA prevention in patients with T1DM treated with SGLT2i.

In summary, our multivariable model suggests that patients with T1DM who are overweight and insulin resistant are at higher risk of DKA when they rapidly reduce insulin dose and are volume depleted, as these conditions concur to trigger unrestricted lipolysis and ketogenesis.

Among non-glycemic benefits, a novel finding of our analysis were the signals for renal and eye protection associated with SGLT2i, with reduced albuminuria and risk of diabetic eye disorders. These 2 outcomes were associated with an improvement in CGM metrics rather than in HbA1c, consistent with emerging evidence that glucose swings are major contributors to microvascular complications [24].

The transient eGFR decline observed in the initial 24 weeks of treatment vanished in RCTs of longer duration (**Table B** in S1 Tables) and could be ascribed to the enhanced afferent arteriolar tone with SGLT2i, which reduce intraglomerular pressure and relieve glomerular hyperfiltration and barrier damage [77,78].

If future RCTs of longer duration translate the observed renal and eye-related effects into hard outcomes, the clinical benefit of SGLT2i may be particularly valuable in patients with T1DM who are at lower DKA risk and have established microvascular complications.

The optimal dose of SGLT2i is also debated: Based on individual trial findings [65], some suggested the lowest dose has equal effectiveness and higher safety than moderate-high doses.

Conversely, we documented a consistent dose-response gradient with increasing SGLT2i dosage for most glycemic and non-glycemic benefits, but not for DKA and other AEs (Table 7).

These findings suggest potential benefits of increasing SGLT2i doses may outweigh the risks of DKA, at least in patients not at increased risk of DKA and within the time frame of included RCTs.

Finally, while some discouraged SGLT2i use in T1DM patients with higher HbA1c levels [18], based on a reported increased incidence of DKA in general T1DM population with HbA1c >10% [79], we did not find a direct relationship between baseline HbA1c and the risk of DKA (Fig 3). Hence, it may be reasonable not to withhold these drugs in patients at greater therapeutic need who are compliant to DKA risk mitigation recommendations.

### Clinical policy implications

Our analysis suggests that the risk of DKA is not uniformly distributed across T1DM population; rather, simple, clinical risk factors are associated with the risk of DKA and with other efficacy and safety outcomes in patients with T1DM treated with SGLT2i.

If confirmed by real-world prospective studies, the results of our analysis may enable the targeted use of SGLT2i in patients with T1DM who have the greatest benefit and the lowest risk of DKA from the use of these drugs.

These findings may be implemented into DKA risk mitigation strategies to appropriately select those patients with a higher baseline benefit-risk ratio from SGLT2i therapy and to inform protocols targeting appropriate TID down-titration and dehydration prevention.

### Strengths and limitations

Strengths and limitations of our analysis derive from the characteristics of included evidence: Strengths include the high percentage of explained variance in DKA risk, the good methodological quality of included RCTs, the thorough assessment of efficacy and safety outcomes, and the relevant impact of extracted evidence on clinical policy and decision-making.

Limitations are the relatively short duration of included trials, which prevented assessment of long-term outcomes. Furthermore, we analyzed study-level characteristics, which do not necessarily correspond to individual patient characteristics, including adherence to prescribed DKA risk mitigation strategies. However, individual patient data are rarely available, as most RCTs are sponsored by industry.

For several outcomes the event rate was low and 95% CIs correspondingly wide, mandating caution in interpreting the absence of statistical significance. Finally, the extrapolation of our findings from RCT analysis to real-world needs confirmation, as participants enrolled in RCTs are inherently different from patients in the realities of clinical practice.

## Conclusions

The data presented show that routinely available clinical parameters are associated with the risk of DKA and the therapeutic response to SGLT2i and that factors associated with therapeutic response differ from those associated with unwanted effects of SGLT2i treatment. These findings may thus represent an initial step toward benefit-risk optimization of SGLT2i use in T1DM. Future studies should refine the predictive ability of our model and assess the clinical benefits of implementing this strategy in real-world practice.

## Supporting information

S1 Text Online search terms. Online search strategies. Online data sources. Definitions PRISMA Checklist.
(DOCX)

S1 Tables **Table A in S1 Tables**. Characteristics (panel A) and risk of bias (panel B) of included trials. **Table B in S1 Tables**. Results of subgroup analysis. **Table C in S1 Tables**. Univariable meta-regression for moderators of the risk ratio of diabetic ketoacidosis (DKA). **Table D in S1 Tables**. Univariable meta-regression for moderators of HbA1c changes (%). **Table E in S1 Tables**. Univariable meta-regression for moderators of BMI changes (%). **Table F in S1 Tables**. Univariable meta-regression for moderators of changes in systolic blood pressure. **Table G in S1 Tables**. Univariable meta-regression for moderators of eGFR changes. **Table H in S1 Tables**. Univariable meta-regression for moderators of ACR changes (mg/g). **Table I in S1 Tables**. Univariable meta-regression for moderators of RR of eye disorders. **Table J in S1 Tables**. Summary of main findings of meta-analysis for safety outcomes in included RCTs. **Table L in S1 Tables**. Univariable meta-regression for moderators of RR for GTI(s). **Table M in S1 Tables**. Univariable meta-regression for moderators of the RR of volume depletion events. **Table N in S1 Tables**. Effect of individual SGLT2 inhibitors on different outcomes. **Table O in S1 Tables**. Results of sensitivity analyses with exclusion of RCTs with high risk of bias, with alternative effect measures, pooling methods, and statistical models. **Table P in S1 Tables**. Sensitivity analysis: fully-adjusted multivariable meta-regression Model 1 and Model 2 for moderators of the risk ratio of diabetic ketoacidosis (DKA); variables significantly associated with the risk of DKA (*p*-value set at 0.15) were entered in Model 3. **Table Q in S1 Tables**. Sensitivity analysis: fully-adjusted multivariable meta-regression Model 1 and Model 2 for moderators of HbA1c changes (%); variables significantly associated with HbA1c changes(%) (with *p*-value set at 0.15) were entered in Model 3. **Table R in S1 Tables**. Quality of evidence for clinically relevant efficacy (panel A) and safety (panel B) outcomes: summary of findings table according to the GRADE approach.
(DOCX)

S1 Figs **Fig A in S1 Figs**. Risk of bias summary: risk of bias item for each included RCT according to Cochrane Risk-of-Bias Tool. **Fig B in S1 Figs**. Risk of bias graph: Each risk of bias item is presented as percentages across all included RCTs. **Fig C in S1 Figs**. Funnel plots for main effect outcomes. **Fig D in S1 Figs**. Forest plot of comparison: SGLT2 inhibitors versus control, outcome: fasting plasma glucose (FPG), continuous glucose monitoring (CGM) parameters time-in-range (70–180 mg/dL) and mean amplitude of glucose excursions (MAGE) and urinary glucose excretion (UGE). **Fig E in S1 Figs**. Forest plot of comparison: SGLT2 inhibitors, outcome: daily total, basal, and bolus insulin dose (%) changes from baseline. **Fig F in S1 Figs**. Forest plot of comparison: SGLT2 inhibitors versus control, outcomes: estimated glucose disposal rate (eGDR) changes (%) (panel 1) and relative insulin sensitivity (RIS) changes (%) (panel 2). **Fig G in S1 Figs**. Forest plot of comparison: SGLT2 inhibitors versus control, outcomes: body mass index (BMI) and systolic BP (sysBP). **Fig H in S1 Figs**. Forest plot of comparison: SGLT2 inhibitor versus control versus placebo, outcomes: eGFR and urinary albumin/creatinine ratio (ACR). **Fig I in S1 Figs**. Forest plot of comparison: SGLT2 inhibitors, outcome: hypoglycemia, severe hypoglycemia, urinary tract infections (UTIs), genital tract infections (GTIs), volume depletion events, eye disorders, and major adverse cardiovascular events (MACE). **Fig L in S1 Figs**. leave-one-out meta-analysis for outcomes DKA and HbA1c (%).
(DOC)

## Abbreviations

ADA: American Diabetes Association
AE: adverse event
BHB: ß-hydroxybutyrate
BMI: body mass index
BP: blood pressure
CGM: continuous glucose monitoring
CSI: continuous subcutaneous infusion
DKA: diabetic ketoacidosis
EASD: European Association for the Study of Diabetes
eGDR: estimated glucose disposal rate
EOT: end of treatment
FFA: free fatty acid
FPG: fasting plasma glucose
GRADE: Grading of Recommendations Assessment, Development, and Evaluation
GTI: genital tract infection
HDL: high-density lipoprotein
INS-SGLT2i: insulin-SGLT2 inhibitor
LDL: low-density lipoprotein
LOCF: last-observation-carried-forward
MACE: major adverse cardiovascular events
MMRM: mixed-effects model for repeated measures
PRISMA: Preferred Reporting Items for Systematic Reviews and Meta-Analyses
RCT: randomized controlled trial
RIS: relative insulin sensitivity
RoB: risk-of-bias
RR: relative risk
SGLT: sodium glucose cotransporter
SGLT2: sodium-glucose cotransporter-2
SGLT2i: sodium-glucose cotransporter-2 inhibitors
sysBP: systolic blood pressure
T1DM: type 1 diabetes mellitus
T2DM: type 2 diabetes mellitus
TID: total insulin dose
UGE: urinary glucose excretion
UTI: urinary tract infection
WMD: weighted mean difference
