# Supplementary material for: Assessing the risk of ketoacidosis due to sodium-glucose cotransporter (SGLT)-2 inhibitors in patients with type 1 diabetes: A meta-analysis and meta-regression
Source: PLoS Med. 2020 Dec 29;17(12):e1003461. doi: 10.1371/journal.pmed.1003461 (PMC7771708; doi:10.1371/journal.pmed.1003461)
Supplement: S1 Text — Online search strategies. Online data sources. Definitions PRISMA Checklist. (DOCX) [file pmed.1003461.s001.docx]

**S1 Text**

**Online Search terms**

Randomized controlled trial, controlled clinical trial, randomized, clinical trial, sodium-glucose transporter 2, sodium-glucose transporter 2, SGLT2, SGLT-2, sodium glucose co-transport 2 inhibitors, SGLT2 inhibitors, dapagliflozin OR Farxiga OR BMS-512148, canagliflozin OR invokana OR JNJ-28431754, Empagliflozin OR Jardiance OR BI10773, ertugliflozin OR Steglatro, Ipragliflozin OR Suglat OR ASP1941, Luseogliflozin OR Lusefi OR TS-071, Tofogliflozin OR Apleway OR Deberza, Ertugliflozin OR PF04971729 OR MK-8835, Bexagliflozin OR EGT0001442, Remogliflozin OR remogliflozin etabonate OR GSK189075 OR ISIS388626 OR ISIS-SGLT2Rx, TA-7284, GW869682 OR sergliflozin etabonate OR sergliflozin, EGT0001474, SHR3824, GSK-1614235, BI44847, sotagliflozin OR LX4211 OR LP802034 OR SAR439954 OR Zynquista, management, therapy, treatment, trial, diabetes, type 1 diabetes.

**Online Search strategies**

**Medline and Cochrane Central Register of Controlled Trials**

1. randomized controlled trial.pt 2. controlled clinical trial.pt 3. randomized.tw 4. clinical trial/ 5. randomly.ab 6. trial.ti 7. placebo.tw 8. 1 or 2 or 3 or 4 or 5 or 6 or 7 9. sodium-glucose transporter 2/ 10. sodium-glucose transporter 2.tw 11. SGLT2.tw 12. SGLT-2.tw 13. Dapagliflozin.tw or Farxiga.tw or BMS-512148.tw 14. Canagliflozin.tw or Invokana.tw or JNJ-28431754.tw 15. Empagliflozin.tw or Jardiance.tw or BI 10773.tw 16. Ipragliflozin.tw or Suglat.tw or ASP1941.tw 17. Luseogliflozin.tw or Lusefi.tw or TS-071.tw 18. Tofogliflozin.tw or Apleway.tw or Deberza.tw 19. Ertugliflozin.tw or PF04971729.tw or MK-8835.tw 20. Sotagliflozin.tw or LX4211.tw or Zynquista.tw 21. Bexagliflozin.tw or EGT0001442.tw 22. Remogliflozin.tw or remogliflozin etabonate.tw. or GSK 189075.tw 23. ISIS 388626.tw or ISIS-SGLT2Rx.tw 24. TA-7284.tw 25. GW 869682.tw or sergliflozin etabonate.tw OR sergliflozin.tw 26. EGT0001474.tw 27. SHR3824.tw 28. GSK-1614235.tw 29. BI 44847.tw 30. 9 or 10 or 11 or 12 or 13 or 14 or 15 or 16 or 17 or 18 or 19 or 20 or 21 or 22 or 23 or 24 or 25 or 26 or 27 or 28 or 29 31. 8 and 30

**EMBASE**

1. 'randomized controlled trial'/exp OR 'randomized controlled trial' 2. 'sodium glucose cotransporter 2'/exp OR 'sodium glucose cotransporter 2' 3. 'sodium glucose cotransporter 2 inhibitor'/exp OR 'sodium glucose cotransporter 2 inhibitor' 4. 'dapagliflozin'/exp OR 'dapagliflozin' OR 'farxiga' OR 'bms 512148' 5. 'canagliflozin'/exp OR 'canagliflozin' OR 'invokana' OR 'jnj 28431754' 6. 'empagliflozin'/exp OR 'empagliflozin' OR 'jardiance' OR 'bi 10773' 7. 'ipragliflozin'/exp OR 'ipragliflozin' OR 'suglat' OR 'asp1941' 8. 'luseogliflozin'/exp OR 'luseogliflozin' OR 'lusefi' OR 'ts-071' 9. 'tofogliflozin'/exp OR 'tofogliflozin' OR 'apleway' OR 'deberza' 10. 'ertugliflozin'/exp OR 'ertugliflozin' OR 'pf04971729' OR 'mk-8835' 11. 'sotagliflozin'/exp OR 'sotagliflozin' OR 'lx4211’ 12. 'bexagliflozin' OR 'egt0001442' 13. 'remogliflozinetabonate'/exp OR 'remogliflozin' OR 'gsk 189075' 14. 'isis 388626' OR 'isis-sglt2rx' 15. 'ta-7284' 16. 'sergliflozinetabonate'/exp OR 'sergliflozin' OR 'gw 869682' 17. 'egt0001474' 18. 'shr3824' 19. 'gsk-1614235' 20. 'bi 44847'/exp OR 'bi 44847' 21. 2 or 3 or 4 or 5 or 6 or 7 or 8 or 9 or 10 or 11 or 12 or 13 or 14 or 15 or 16 or 17 or 18 or 19 or 20 22. 1 and 21

**CLINICALTRIALS.GOV**

1. Sodium-Glucose Transporter 2 2. SGLT2 3. Dapagliflozin 4. Canagliflozin 5. Empagliflozin.tw 6. Ipragliflozin.tw 7. Luseogliflozin.tw 8. Topogliflozin.tw 9. Remogliflozin.tw 10. PF04971729.tw 11. LX4211.tw 12. EGT0001442.tw 13. TS-071.tw 14. GW 869682.tw 15. ISIS 388626.tw

**FDA EMA and PMDA databases**

1. Sodium-Glucose Transporter 2 2. SGLT2 3. Dapagliflozin 4. Canagliflozin 5. Empagliflozin 6. Ipragliflozin 7. Luseogliflozin 8. Topogliflozin 9. Remogliflozin

**Online Data Sources**

The following online electronic databases and clinical trial registries were searched: Ovid MEDLINE, Ovid MEDLINE Epub Ahead of Print, Ovid MEDLINE In-Process, EMBASE, Cochrane CENTRAL Register of Controlled Trials Cochrane Database of Systematic Reviews, Epistemonikos, [ClinicalTrials.gov](http://www.clinicaltrials.gov/), World Health Organization International Clinical Trials Registry Platform, European Union (EU) Clinical Trials Register, International Standard Randomized Controlled Trial Number (ISRCTN) registry, Australian New Zealand Clinical Trials Registry, and additional national clinical trial registries(see below):

**International and National Trial registries search results**

-**World Health Organization-International Clinical Trials Registry Platform** ([http://apps.who.int
/trialsearch/](http://apps.who.int/trialsearch/)): 237 records

-**ClinicalTrials.gov**(<https://www.clinicaltrials.gov/ct2/home>): 97 records

-**Cochrane CENTRAL Register of Controlled Trials** (<https://www.cochranelibrary.com/central/about-central>): 107 records

- **European Union(EU) Clinical Trials Register** (<https://www.clinicaltrialsregister.eu/>): 39 records

-**ISRCTN** (<http://www.isrctn.com/> ): 18 results

***-*Epistemonikos** *(*<https://www.epistemonikos.org/>): 11 records

-**Health Canada Clinical Trial Database** (<http://www.hc-sc.gc.ca/dhp-mps/prodpharma/databasdonclin/index-eng.php>): 21 records

-**German Clinical Trials Register** (<https://drks-neu.uniklinik-freiburg.de/drks_web/>):  8 results

-**Netherlands Trial Register (Dutch**) (<http://www.trialregister.nl/trialreg/index.asp>): 0 results

-**Swiss National Clinical Trials Portal** ([http://www.kofam.ch/en/swiss -clinical-trials-portal.html](http://www.kofam.ch/en/swiss%20-clinical-trials-portal.html)) 6 results

-**Australian New Zealand Clinical Trials Registry** (<http://www.anzctr.org.au/>): 6 records

-**Chinese Clinical Trial Register** (<http://www.chictr.org.cn/enIndex.aspx>): 0 records

-**Clinical Trials Registry–India**(<http://ctri.nic.in/>): 1 record

**-Iranian Registry of Clinical Trials** (<http://www.irct.ir/>):  0 records

**-Japan Primary Registries Network** (<http://rctportal.niph.go.jp/>): 0 records

-**Clinical Research Information Service, Republic of Korea** (<https://cris.nih.go.kr/cris/en/use_guide/cris_introduce.jsp>): 0 records

-**Philippine Health Research Registry** ([http://registry. healthresearch.ph/](http://registry.healthresearch.ph/)): 0 results

-**Sri Lanka Clinical Trials Registry** (<http://www.slctr.lk/>): 0 records

**-Thai Clinical Trials Registry** (<http://www.clinicaltrials.in.th/>): 0 records

-**Brazilian Clinical Trials Registry** (<http://www.ensaiosclinicos.gov.br/>): 0 records

-**Public Cuban Registry of Clinical Trials** (<http://registroclinico.sld.cu/en/home>): 0 records

**-Peruvian Registry of Clinical Trials** ([http://www.ins.gob.pe/ ensayosclinicos/](http://www.ins.gob.pe/%20ensayosclinicos/)): 0 records

**-Pan African Clinical Trials Registry** (<http://www.pactr.org/>): 0 records

-**South African National Clinical Trials Register**: (<http://www.sanctr.gov.za/>): 0 records

**-Tanzania Clinical Trial Registry** (<http://www.tzctr.or.tz/>): 0 records

**Regulatory Agencies sites search results**

**US Food and Drug Administration (FDA)**: <https://search.usa.gov/search?query=SGLT2+inhibitors&affiliate=fda1>: 100 results

**European Medicines Agency (EMA)**: <https://www.ema.europa.eu/en/search/search?search_api_views_fulltext=SGLT2%20inhibitors>: 819 results

**Japanese Pharmaceutical and Medical Devices Agency (PMDA)**: <https://ss.pmda.go.jp/en_all/search.x?q=SGLT2+inhibitors&ie=UTF-8&page=1>: 34 results

**Definitions**

**Definite diabetic ketoacidosis (DKA):** DKA was diagnosed based on evidence of anion-gap metabolic acidosis related to excessive ketone production without a satisfactory alternative cause for anion-gap acidosis, as outlined by Kitabchi et al. [ref 18,22 of main text].

**Indices of Insulin Sensitivity**

Insulin resistance in T1DM (so-called “double diabetes”) contributes to macro- and micro-vascular complications independently of glycemic control, and may predispose to DKA by promoting unrestricted lipolysis of free fatty acids (FFAs) from adipose tissue to the liver.

We therefore evaluated the effect of treatment on insulin sensitivity as assessed via two independent methods: through the estimated Glucose Disposal Rate (eGDR, mg/kg/min), which was validated against euglycemic hyperinsulinemic clamp and predicted incident CVD and microvascular complications in T1DM independently of glycemic control, metabolic syndrome and overweight[^[[1]](#endnote-1)^,^[[2]](#endnote-2)^]

**Estimated Glucose Disposal Rate(eGDR)**

The eGDR (mg/kg/min) was calculated as previously described: eGDR (mg x kg−1 x min−1)

= 21.158 − [0.090 × WC (cm)] - [3.407 × HT] − [0.551 × HbA1C], where

WC = waist circumference (cm), HT = hypertension (yes = 1/no = 0) and HbA1c = HbA1c (%).

**Relative Insulin Sensitivity(RIS)**

Relative Insulin Sensitivity (RIS) is based on changes in daily Total Insulin Dose(TID)/kg body weight

The mean insulin sensitivity (IS) values in well-controlled pump T1DM patients are 0.53 IU per kg of body weight. Any insulin-deficient individual’s relative insulin sensitivity (RIS) can then be determined by dividing an expected TID for their weight [(0.53 IU/kg. × body weight(kg) by their actual TID: RIS = expected daily TID **/** actual daily TID = [0.53 × body weight(kg)]**/** TID (IU/day).

On this RIS scale, a value of 1.0 represents an average IS, less than 1.0 would be a decreased sensitivity, and greater than 1.0 would be an increased IS.

**SGLT2 inhibitor dose categorization**

Dapagliflozin, 10 mg was considered high dose, 5 mg moderate, and both 2.5 mg and 1 mg of dapagliflozin were classified into low-dose subgroup; Empagliflozin at5 mg, 10 mg and 25 mg, sotagliflozin 75 mg, 200 mg and 400 mg, as well as ipragliflozin at 25 mg, 50 mg and 100 mg were respectively separated into low-, moderate- and high-dose dose subgroups; Canagliflozin at 100 mg and 300 mg was classified into moderate-dose and high-dose subgroups.respectively.

**Residual insulin-SGLT2 inhibitor (INS-SGLT2i) effect**

The pharmacodynamic effect of SGLT2 inhibitors (SGLTi) was estimated as a virtual "insulin dose" using 24-hour UGE values and a recognized insulin-to-carbohydrate counting technique[ref 28, 29, 48 of the main text].

Following an assumption that the glucose lost to urine from SGLT2 inhibitor treatment (ie, UGE) is derived from carbohydrate metabolism^[[3]](#endnote-3)^ and using an existing and recognized insulin-to-carbohydrate counting technique (the “450 rule”; I:C ratio = 450/TID at baseline), together with 24-hour UGE, we estimated a virtual “insulin dose”, equivalent to the amount of glucose cleared in the urine as a result of the pharmacodynamic effect of SGLT2 inhibitors over 24 hours (intestinal SGLT1 inhibition delays, but does not reduce glucose absorption from the intestine, see ref 5 of the main text).

This virtual “insulin dose”, called the INS:24-hour-SGLT2i equivalent, was calculated with the following formula,using data from each RCT: INS:24-hour-SGLTi equivalent” =UGE/I:C.

Expressed as a percentage of TID at baseline, this formula becomes:

“INS:24-hr-SGLT2i equivalent” =UGE/[(I:C) x baseline TID] X 100

The “INS:24-hrSGLT2i equivalent” (%) values were then compared with the actual TID reductions (%) observed in each RCT for each treatment arm, to determine the level of agreement. The difference between the “INS:24-hr-SGLT2i equivalent” and the observed TID reduction was termed the “*Residual INS-SGLT2i effect*” and expressed as a percentage of baseline TID:

Residual INS-SGLT2i effect(%)=

{[(Observed TIDat end-of-treatment−baselineTID) + “INS:24-hr-SGLT2i equivalent] /

baselineTID} x 100.

A TID reduction approaching the residual INS-SGT2i effect has been suggested to restrict DKA risk while also reducing hypoglycemic risk in a phase 2 RCT[47 of main text].

The association between “Residual INS-DAPA effect” (%) and the risk of DKA and of hypoglycemia was then investigated.

**Renal event:** defined according to the following Medical Dictionary for Regulatory Activities preferred

terms:

Acute prerenal failure; Anuria; Azotemia; Blood creatine abnormal; Blood creatine decreased; Blood creatine increased; Blood creatinine abnormal; Blood creatinine decreased; Blood creatinine increased

Blood urea abnormal; Blood urea increased; Blood urea nitrogen/creatinine ratioincreased

Coma uremic; Computerized tomogram kidney abnormal; Creatine urine abnormal; Creatine urine decreased; Creatine urine increased; Creatinine renal clearance abnormal

Creatinine renal clearance decreased; Creatinine urine abnormal; Creatinine urine decreased

Creatinine urine increased; Cystatin C abnormal; Cystatin C increased, Diabetic end stage renal disease; Glomerular filtration rate abnormal; Glomerular filtration rate decreased;

Glomerular filtration rate increased; Hypercreatinemia; Hyperparathyroidism secondary

Inulin renal clearance abnormal; Inulin renal clearance decreased; Kidney fibrosis;

Nephrogenic anemia; Nitrogen balance negative; Edema due to renaldisease;

Oliguria Pericarditis uremic Phenolsulfonphthalein test abnormal; Postoperative renal failure

Prerenal failure; Renal cortical necrosis; Renal disorder; Renal failure;

Renal failure acute; Renal failure chronic; Renal function test abnormal; Renal impairment;

Renal injury; Renalnecrosis; Renal papillary necrosis; Renal scan abnormal; Renal tubular acidosis; Renal tubular atrophy; Renal tubular disorder; Renal tubular necrosis; Ultrasound kidney abnormal;Uremicacidosis;Uremicencephalopathy;UremicgastropathyUremic neuropathy;

Uremic pruritus; Urea renal clearance; Urea renal clearance decreased; Urea renal clearance increased; Uridosis; Urine albumin/creatinine ratio abnormal; Urine albumin/creatinine ratio decreased;

Urine albumin/creatinine ratio increased; Urine output; Urine output decreased; Urine output increased;

Urine protein/creatinine ration abnormal; Urine protein/creatinine ratio decreased;

Urine protein/creatinine ratio increased.

**Volume depletion event:** defined according to the following Medical Dictionary for Regulatory

Activities preferred terms:

Acute prerenal failure; Blood pressure abnormal; Blood pressure ambulatory abnormal; Blood pressure decreased; Blood pressure diastolic abnormal; Blood pressure diastolic decreased; Blood pressure fluctuation; Blood pressure immeasurable; Blood pressure inadequately controlled; Blood pressure orthostasis abnormal; Blood pressure orthostatic decreased; Blood pressure systolic abnormal; Blood pressure systolic decreased; Blood pressure systolic inspiratory decreased; Brachial pulse abnormal;

Brachial pulse decreased; BUN/creatinine ratio increased; Capillary nail refill test abnormal; Cardiac index abnormal; Cardiac index decreased; Cardiac output decreased; Cardiovascular insufficient; Carotid pulse abnormal; Carotid pulse decreased; Central venous pressure abnormal; Central venous pressure decreased;Circulatorycollapse;Decreasedventricularpreload;Dehydration;Diastolichypotension;Femoral pulse abnormal; Femoral pulse decreased; Hemodynamic test abnormal; Heart rate abnormal; Heart rate decreased;

Heart rate increased; Hypoperfusion; Hypotension; Hypovolemia; Hypovolemic shock;

Labile blood pressure; Left ventricular end-diastolic pressure decreased; Maximum heart rate decreased;

Mean arterial pressure decreased; Orthostatic heart rate response increased; Orthostatic hypotension;

Orthostatic intolerance; Pedal pulse abnormal; Pedal pulse decreased; Peripheral circulatory failure; Peripheral coldness; Peripheral pulse decreased; Popliteal pulse abnormal; Popliteal pulse decreased;

Prerenal failure; Presyncope; Pulseabnormal; Pulseabsent; Pulse pressure abnormal; Pulse pressure decreased; Pulse volume decreased; Pulse waveform abnormal; Radial pulse abnormal; Radial pulse decreased; Renal ischemia; Schelling test; Shock; Syncope;Thirst; Tilt table test positive; Urine albumin/creatinine ratio increased; Urine flow decreased; Urine output decreased;

Urine protein/creatinine ratio increased; Vascular test abnormal; Venous pressure abnormal;

Venous pressure decreased; Venous pressure jugular abnormal; Venous pressure jugular decreased;

Volume blood decreased.

**Management of missing data.**

Missing data were managed by contacting via e-mail the corresponding authors of the RCTs. Where this was unsuccessful, we planned to calculate missing data from the raw numbers given in tables and/or estimated from bar charts.

When missing SDs of mean change in parameters were missing, we planned to calculate as mentioned in the Cochrane Handbook of Systematic Reviews(chapter 7.6-7.8 and 16.1.3)(ref. 31 of main text).

Where the p value was provided for a comparison between treated and control groups, we planned to calculate the standard deviation by converting the p value into a t value with appropriate degrees of freedom, and then calculating standard error and standard deviation. If neither the standard deviations nor the p values were supplied, we planned to impute a standard deviation from studies with similar measurement methods, duration and measurement error was used if available1 and tested in a sensitivity analysis and reported if the estimate differed meaningfully from previous estimates. If no similar studies were available, a narrative approach would have been used to summarize the data

**PRISMA Checklist**

| **Section/topic** | **#** | **PRISMA Checklist item** | **Reported on Section- paragraph #** |
| --- | --- | --- | --- |
| **TITLE** | | |  |
| Title | 1 | Identify the report as a systematic review, meta-analysis, or both. | Title page |
| **ABSTRACT** | | |  |
| Structured summary | 2 | Provide a structured summary including, as applicable: background; objectives; data sources; study eligibility criteria, participants, and interventions; study appraisal and synthesis methods; results; limitations; conclusions and implications of key findings; systematic review registration number. | Abstract |
| **INTRODUCTION** | | |  |
| Rationale | 3 | Describe the rationale for the review in the context of what is already known. | Para 2 |
| Objectives | 4 | Provide an explicit statement of questions being addressed with reference to participants, interventions, comparisons, outcomes, and study design (PICOS). | Para 2 |
| **METHODS** | | |  |
| Protocol and registration | 5 | Indicate if a review protocol exists, if and where it can be accessed (e.g., Web address), and, if available, provide registration information including registration number. | Methods  22^nd^ para |
| Eligibility criteria | 6 | Specify study characteristics (e.g., PICOS, length of follow-up) and report characteristics (e.g., years considered, language, publication status) used as criteria for eligibility, giving rationale. | Methods 4^th^ para |
| Information sources | 7 | Describe all information sources (e.g., databases with dates of coverage, contact with study authors to identify additional studies) in the search and date last searched. | Methods 1^st^ para |
| Search | 8 | Present full electronic search strategy for at least one database, including any limits used, such that it could be repeated. | S1 suppl. text |
| Study selection | 9 | State the process for selecting studies (i.e., screening, eligibility, included in systematic review, and, if applicable, included in the meta-analysis). | Figure 1 |
| Data collection process | 10 | Describe method of data extraction from reports (e.g., piloted forms, independently, in duplicate) and any processes for obtaining and confirming data from investigators. | Methods 6^th^ para |
| Data items | 11 | List and define all variables for which data were sought (e.g., PICOS, funding sources) and any assumptions and simplifications made. | Methods 1^st^-6^th^ para, Table 1 |
| Risk of bias in individual studies | 12 | Describe methods used for assessing risk of bias of individual studies (including specification of whether this was done at the study or outcome level), and how this information is to be used in any data synthesis. | Methods 6^th^ para  S1 table 2 |
| Summary measures | 13 | State the principal summary measures (e.g., risk ratio, difference in means). | Methods 7^th^ parag |
| Synthesis of results | 14 | Describe the methods of handling data and combining results of studies, if done, including measures of consistency (e.g., I^2^) for each meta-analysis. | Methods 7^th^-8^th^ para |

1. [Kilpatrick ES](https://www.ncbi.nlm.nih.gov/pubmed/?term=Kilpatrick%20ES%5BAuthor%5D&cauthor=true&cauthor_uid=17327345), [Rigby AS](https://www.ncbi.nlm.nih.gov/pubmed/?term=Rigby%20AS%5BAuthor%5D&cauthor=true&cauthor_uid=17327345), [Atkin SL](https://www.ncbi.nlm.nih.gov/pubmed/?term=Atkin%20SL%5BAuthor%5D&cauthor=true&cauthor_uid=17327345). Insulin resistance, the metabolic syndrome, and complication risk in type 1 diabetes: "double diabetes" in the Diabetes Control and Complications Trial. [Diabetes Care.](https://www.ncbi.nlm.nih.gov/pubmed/17327345) 2007;30:707-12. [↑](#endnote-ref-1)
2. [Merger SR](https://www.ncbi.nlm.nih.gov/pubmed/?term=Merger%20SR%5BAuthor%5D&cauthor=true&cauthor_uid=27449710), [Kerner W](https://www.ncbi.nlm.nih.gov/pubmed/?term=Kerner%20W%5BAuthor%5D&cauthor=true&cauthor_uid=27449710), [Stadler M](https://www.ncbi.nlm.nih.gov/pubmed/?term=Stadler%20M%5BAuthor%5D&cauthor=true&cauthor_uid=27449710), [Zeyfang A](https://www.ncbi.nlm.nih.gov/pubmed/?term=Zeyfang%20A%5BAuthor%5D&cauthor=true&cauthor_uid=27449710), [Jehle P](https://www.ncbi.nlm.nih.gov/pubmed/?term=Jehle%20P%5BAuthor%5D&cauthor=true&cauthor_uid=27449710), [Müller-Korbsch M](https://www.ncbi.nlm.nih.gov/pubmed/?term=M%C3%BCller-Korbsch%20M%5BAuthor%5D&cauthor=true&cauthor_uid=27449710), et al. Prevalence and comorbidities of double diabetes. [Diabetes Res Clin Pract.](https://www.ncbi.nlm.nih.gov/pubmed/27449710) 2016;119:48-56. [↑](#endnote-ref-2)
3. Monnier L, Colette C. Target for glycemic control: concentrating onglucose. Diabetes Care. 2009;32(suppl 2):S199-S204. [↑](#endnote-ref-3)
