# Supplementary material for: Assessing the risk of ketoacidosis due to sodium-glucose cotransporter (SGLT)-2 inhibitors in patients with type 1 diabetes: A meta-analysis and meta-regression
Source: PLoS Med. 2020 Dec 29;17(12):e1003461. doi: 10.1371/journal.pmed.1003461 (PMC7771708; doi:10.1371/journal.pmed.1003461)
Supplement: S1 Tables — Table A in S1 Tables. Characteristics (panel A) and risk of bias (panel B) of included trials. Table B in S1 Tables. Results of subgroup analysis. Table C in S1 Tables. Univariable meta-regression for moderators of the risk ratio of diabetic ketoacidosis (DKA). Table D in S1 Tables. Univariable meta-regression for moderators of HbA1c changes (%). Table E in S1 Tables. Univariable meta-regression for moderators of BMI changes (%). Table F in S1 Tables. Univariable meta-regression for moderators of changes in systolic blood pressure. Table G in S1 Tables. Univariable meta-regression for moderators of eGFR changes. Table H in S1 Tables. Univariable meta-regression for moderators of ACR changes (mg/g). Table I in S1 Tables. Univariable meta-regression for moderators of RR of eye disorders. Table J in S1 Tables. Summary of main findings of meta-analysis for safety outcomes in included RCTs. Table L in S1 Tables. Univariable meta-regression for moderators of RR for GTI(s). Table M in S1 Tables. Univariable meta-regression for moderators of the RR of volume depletion events. Table N in S1 Tables. Effect of individual SGLT2 inhibitors on different outcomes. Table O in S1 Tables. Results of sensitivity analyses with exclusion of RCTs with high risk of bias, with alternative effect measures, pooling methods, and statistical models. Table P in S1 Tables. Sensitivity analysis: fully-adjusted multivariable meta-regression Model 1 and Model 2 for moderators of the risk ratio of diabetic ketoacidosis (DKA); variables significantly associated with the risk of DKA (p-value set at 0.15) were entered in Model 3. Table Q in S1 Tables. Sensitivity analysis: fully-adjusted multivariable meta-regression Model 1 and Model 2 for moderators of HbA1c changes (%); variables significantly associated with HbA1c changes(%) (with p-value set at 0.15) were entered in Model 3. Table R in S1 Tables. Quality of evidence for clinically relevant efficacy (panel A) and safety (panel B) outcomes: summary [file pmed.1003461.s002.docx]

**S1 Tables**

**S1 Table A. Characteristics (panel A) and Risk of Bias (panel B) of included trials.**

| **Supplementary Table A panel 1. Characteristics of included randomized controlled trials** | **Renal function**(ml/min/1.73 m^2)^ | eGFR ≥60  ml/min/1.73 m^2^ | | | | | eGFR >60  ml/min/1.73 m^2^ | | |
| --- | --- | --- | --- | --- | --- | --- | --- | --- | --- |
|  | **Dropout rate (%)** | 7.7% | 7.7% | 0% | 21.4% | 20% | 0% | 0% | 0% |
|  | **Background treatment/**  **Daily TID (IU/kg)** | 0.62 IU/kg | 0.74 IU/kg | 0.85 IU/kg | 0.59 IU/kg | 0.73 IU/kg | 0.65 IU/kg | 0.61 IU/kg | 0.64 IU/kg |
|  | **Diabetes duration (yr)** | 16 | 20 | 22 | 17 | 18 | 17 | 16 | 15 |
|  | **eGDR**  **(mg/kg/min)** | 8.42 | 8.74 | 8.62 | 8.92 | 8.43 | 9.11 | 9.28 | 9.45 |
|  | **HbA1c**  **(%)** | 8.7 | 8.2 | 8.4 | 8.5 | 8.4 | 8.1 | 7.9 | 7.9 |
|  | **Bodyweight (kg)**  **/ BMI (kg/m^2^)** | 78 kg/ 25.3 | 77 kg/ 25.1 | 77.4 kg/ 24.8 | 67.3 kg/ 23.4 | 78.4 kg/ 25.8 | 57.2 kg/ 22.9 | 61.6 kg/ 23 | 59.8 kg/ 22.2 |
|  | **Gender**  **(%M)** | 61 | 38 | 73 | 57 | 53 | 21 | 57 | 50 |
|  | **Age (yr)** | 34 | 34 | 36 | 35 | 38 | 43 | 37 | 37 |
|  | **Study**  **Arms** | placebo | Dapa 1 mg | Dapa2.5 mg | Dapa 5 mg | Dapa 10 mg | placebo | Dapa 5 mg | Dapa 10 mg |
|  | **Study duration (week)** | 2 | | | | | 1 | | |
|  | **N** | 70 | | | | | 42 | | |
|  | **Author** | **Henry 2015** | | | | | **Watada 2018** | | |

| **Supplementary Table A panel 2. Characteristics of included randomized controlled trials** | **Renal function**(ml/min/1.73 m^2)^ | eGFR >30  ml/min/1.73 m^2^ | | eGFR ≥60  ml/min/1.73 m^2^ | | | eGFR ≥60  ml/min/1.73 m^2^ | | |
| --- | --- | --- | --- | --- | --- | --- | --- | --- | --- |
|  | **Dropout rate**  **(%)** | 10% | 15% | 9.1% | 8.7% | 10.7% | 12.1% | 10% | 9.3% |
|  | **Background treatment /**  **Daily TID (IU/kg)** | 0.57 IU/kg+ liraglutide | 0.70 IU/kg+ liraglutide | 0.74 IU/kg | 0.76 IU/kg | 0.71 IU/kg | 0.71 IU/kg | 0.73 IU/kg | 0.73 IU/kg |
|  | **Diabetes duration (yr)** | 31 | 25 | 21 | 20 | 20 | 19 | 19 | 19 |
|  | **eGDR**  (mg/kg/min) | 8.50 | 7.39 | 7.09 | 7.24 | 7.25 | 8.12 | 8.18 | 8.39 |
|  | **HbA1c((%)** | 7.4 | 7.8 | 8.5 | 8.5 | 8.5 | 8.4 | 8.4 | 8.4 |
|  | **Bodyweight (kg)**  **/ BMI (kg/m^2^)** | 79 kg/ 27 | 85 kg/ 31 | 84.4 kg 28.6 | 81 kg/ 28.4 | 82.1 kg/ 28.2 | 78.8 kg/ 27.6 | 78.7 kg/ 27.2 | 80.1 kg/ 27.8 |
|  | **Gender**  **(%M)** | 33 | 41 | 51 | 43 | 50 | 44 | 44 | 45 |
|  | **Age (yr)** | 52 | 55 | 43 | 42 | 43 | 43 | 43 | 42 |
|  | **Study Arms** | Placebo | Dapa 10 mg | placebo | Dapa 5 mg | Dapa 10 mg | placebo | Dapa 5 mg | Dapa 10 mg |
|  | **Study duration (week)** | 12 | | 52 | | | 52 | | |
|  | **N** | 30 | | 833 | | | 813 | | |
|  | **Author** | **Kuhadya 2016** | | **Dandona 2017** | | | **Mathieu 2018** | | |

| **Supplementary Table 1 panel A. Characteristics of included randomized controlled trials** | **Renal function**(ml/min/1.73 m^2)^ | eGFR >60  ml/min/1.73 m^2^ | | | | eGFR >60  ml/min/1.73 m^2^ | | eGFR >60  ml/min/1.73 m^2^ | | |
| --- | --- | --- | --- | --- | --- | --- | --- | --- | --- | --- |
|  | **Dropout rate(%)** | 9% | 0% | 0% | 0% | 10% | 3% | 8.5% | 5.1% | 6% |
|  | **Background treatment /**  **Daily TID (IU/kg)** | 0.69 IU/kg | 0.69 IU/kg | 0.60 IU/kg | 0.78 IU/kg | 0.76 IU/kg | 0.74 IU/kg | 0.69 IU/kg | 0.62 IU/kg | 0.63 IU/kg |
|  | **Diabetes duration (yr)** | 18 | 19 | 15 | 11 | 14 | 13 | 23 | 22 | 22 |
|  | **eGDR**  (mg/kg/min) | 8.64 | 8.65 | 8.57 | 8.52 | 8.72 | 8.64 | 8.16 | 8.18 | 8.06 |
|  | **HbA1c**  **(%)** | 8.7 | 8.5 | 8.4 | 8.8 | 8.7 | 8.7 | 7.9 | 7.9 | 8 |
|  | **Bodyweight (kg)**  **/ BMI (kg/m^2^)** | 63.1 kg/23.9 | 67.3 kg/ 26.6 | 64.8 kg/ 24.5 | 66.5 kg/ 24.3 | 64.7 kg/24.2 | 66.1 kg/24.7 | 83 kg/ 28 | 84.1 kg/ 28 | 82.9 kg/ 28.1 |
|  | **Gender**  **(%M)** | 30 | 33 | 17 | 40 | 46 | 47 | 54 | 59 | 56 |
|  | **Age (yr)** | 45 | 47 | 43 | 42 | 48 | 49 | 42 | 42 | 43 |
|  | **Study Arms** | placebo | Ipra 25 mg | Ipra 50 mg | Ipra 100 mg | placebo | Ipra 50 mg | placebo | Cana 100 mg | Cana 300 mg |
|  | **Study duration (week)** | 2 | | | | 24 | | 18 | | |
|  | **N** | 43 | | | | 175 | | 351 | | |
|  | **Author** | **Kaku 2019** | | | | **Kaku (a) 2019** | | **Henry 2015** | | |

| 42 | **Renal Renal function**(ml/min/1.73 m^2)^ | eGFR≥60  ml/min/1.73 m^2^ | | | | eGFR≥30  ml/min/1.73 m^2^ | | | eGFR≥30  ml/min/1.73 m^2^ | | | |
| --- | --- | --- | --- | --- | --- | --- | --- | --- | --- | --- | --- | --- |
|  | **Dropout rate(%)** | 0% | | | | 13% | 8.7% | 5.4% | 7.4% | 4.1% | 5.2% | 4.9% |
|  | **Background treatment /**  **Daily TID (IU/kg)** | 0.66 IU/kg | 0.65 IU/kg | 0.71 IU/kg | 0.65 IU/kg | 0.70 U/kg | 0.70 U/kg | 0.74 U/kg | 0.70 U/kg | 0.70 U/kg | 0.71 U/kg | 0.71 U/kg |
|  | **Diabetes duration (yr)** | 20 | 20 | 16 | 24 | 22 | 23 | 22 | 22 | 21 | 20 | 21 |
|  | **eGDR**  (mg/kg/min) | 8.55 | 8.61 | 8.19 | 8.57 | 8.15 | 7.90 | 7.90 | 8.37 | 8.38 | 7.94 | 8.06 |
|  | **HbA1c**  **(%)** | 8.2 | 8.3 | 8.3 | 8.1 | 8.1 | 8.1 | 8.1 | 8.2 | 8.1 | 8.2 | 8.2 |
|  | **Bodyweight (kg) / BMI (kg/m^2^)** | 79.8 kg/ 25.4 | 75.9 kg/ 24.7 | 87.1 kg/ 27.4 | 76.9 kg/ 25.4 | 83.4 kg/ 28.5 | 86.2 kg/ 29.5 | 85.6 kg/ 29.5 | 80.7 kg/ 27.8 | 81.6 kg/ 28 | 83.7 kg/ 28.7 | 83.3 kg/ 28.4 |
|  | **Gender**  **(%M)** | 68 | 79 | 79 | 56 | 46 | 49 | 46 | 48 | 50 | 47 | 51 |
|  | **Age (yr)** | 41 | 42 | 40 | 42 | 44 | 46 | 45 | 44 | 44 | 44 | 47 |
|  | **Study Arms** | placebo | Empa 2.5 mg | Empa10 mg | Empa 25 mg | placebo | Empa 10 mg | Empa 25 mg | placebo | Empa 2.5 mg | Empa 10 mg | Empa 25 mg |
|  | **Study duration (week)** | 4 | | | | 52 | | | 26 | | | |
|  | **N** | 75 | | | | 730 | | | 975 | | | |
|  | **Author** | **Pieber 2015** | | | | **Rosenstock 2018 )EASE-2)** | | | **Rosenstock 2018 )EASE-3)** | | | |

| **Supplementary Table 1 panel A. Characteristics of included randomized controlled trials** | **Renal function** | eGFR≥60 ml/min/1.73 m^2^ | | | | eGFR≥60 ml/min/1.73 m^2^ | |
| --- | --- | --- | --- | --- | --- | --- | --- |
|  | **Dropout rate(%)** | 0% | 8.3% | 0% | 0% | 0% | 0% |
|  | **Background treatment /**  **Daily TID (IU/kg)** | 0.71 U/kg | 0.73 U/kg | 0.73 U/kg | 0.66 U/kg | Insulin 0.6 IU/kg | Insulin 0.6 IU/kg |
|  | **Diabetes duration (yr)** | 15 | 17 | 14 | 21 | 18.5 | 16.8 |
|  | **eGDR**  (mg/kg/min) | 8.87 | 8.82 | 9.13 | 9.25 | 8.46 | 8.27 |
|  | **HbA1c**  **(%)** | 8.2 | 8 | 8.1 | 7.9 | 7.94 | 7.98 |
|  | **Bodyweight (kg) / BMI (kg/m^2^)** | 63.6 kg/ 23.7 | 63.3 kg/ 24.4 | 59.9 kg/ 22.7 | 60.5 kg/ 22.6 | 74.2 kg /  27.1 kg/m^2^ | 72.7 kg / 26.2 |
|  | **Gender**  **(%M)** | 45 | 38 | 33 | 67 | 50 | 47 |
|  | **Age (yr)** | 44 | 44 | 44 | 47 | 45 | 44 |
|  | **Study Arms** | placebo | Empa 2.5 mg | Empa 10 mg | Empa 25 mg | Sota 400 mg | placebo |
|  | **Study duration (week)** | 4 | | | | 4 | |
|  | **N** | 48 | | | | 33 | |
|  | **Author** | **Shimada 2018** | | | | **Sands 2015** | |

|  | **Renal function** | eGFR≥45  ml/min/1.73 m^2^ | | eGFR≥60  ml/min/1.73 m^2^ | | | |
| --- | --- | --- | --- | --- | --- | --- | --- |
|  | **Dropout rate(%)** | 0% | 4.8% | 0% | 0% | 2.7% | 2.7% |
|  | **Background treatment /**  **Daily TID (IU/kg)** | Insulin 0.8 IU/kg | Insulin 0.8 IU/kg | Insulin  0.7 IU/kg | Insulin  0.7 IU/kg | Insulin  0.7 IU/kg | Insulin  0.7 IU/kg |
|  | **Diabetes duration (yr)** | 12 | 12 | 24 | 24 | 23 | 27 |
|  | **eGDR**  (mg/kg/min) | 6.95 | 7.39 | 6.62 | 7.19 | 8.01 | 7.37 |
|  | **HbA1c**  **(%)** | 9.9 | 9.7 | 8.1 | 8.1 | 8.0 | 8.0 |
|  | **Bodyweight (kg) / BMI (kg/m^2^)** | 83.8 kg / 29.0 | 80.7 kg / 27.9 | 84.1 kg / 29 | 81.9 kg / 28 | 78.1 kg / 27 | 89.6 kg /31 |
|  | **Gender**  **(%M)** | 49 | 45 | 57 | 47 | 40 | 42 |
|  | **Age (yr)** | 23 | 22 | 45 | 47 | 42 | 48 |
|  | **Study Arms** | Sota 400 mg | placebo | Sota 400 mg | Sota 200 mg | Sota 75 mg | placebo |
|  | **Study duration (week)** | 12 | | 12 | | | |
|  | **N** | 87 | | 141 | | | |
|  | **Author** | **Bode 2017** | | **Baker 2017** | | | |

| **Renal function** | eGFR≥45  ml/min/1.73 m^2^ | | eGFR≥45  ml/min/1.73 m^2^ | | | eGFR≥45  ml/min/1.73 m^2^ | | |
| --- | --- | --- | --- | --- | --- | --- | --- | --- |
| **Dropout rate** | 13% | 11% | 10% | 9% | 12% | 8% | 8% | 8% |
| **Background treatment /**  **Daily TID (IU/kg)** | Insulin  0.7 IU/kg | Insulin  0.7 IU/kg | Insulin  0.7 IU/kg | Insulin  0.7 IU/kg | Insulin  0.7 IU/kg | Insulin  0.7 IU/kg | Insulin  0.7 IU/kg | Insulin  0.7 IU/kg |
| **Diabetes duration (yr)** | 20 | 20 | 24 | 25 | 24 | 19 | 18 | 18 |
| **eGDR**  (mg/kg/min) | 6.92 | 6.98 | 7.14 | 7.09 | 7.18 | 7.27 | 7.15 | 7.27 |
| **HbA1c**  **(%)** | 8.2 | 8.2 | 7.6 | 7.6 | 7.5 | 7.7 | 7.7 | 7.7 |
| **Bodyweight (kg) / BMI (kg/m^2^)** | 82.4 kg/ 28.3 | 81.6/28.1 | 86.5 kg/29.6 | 86.9 kg/29.8 | 87.3 kg/29.6 | 81.9 kg/ 27.9 | 81.9 kg/ 27.9 | 81.1 kg/ 27.5 |
| **Gender**  **(%M)** | 51 | 48 | 46 | 48 | 51 | 51 | 53 | 52 |
| **Age (yr)** | 43 | 42 | 46 | 47 | 45 | 41 | 42 | 40 |
| **Study Arms** | Sota 400 mg | placebo | Sota 400 mg | Sota 200 mg | placebo | Sota 400 mg | Sota 200 mg | placebo |
| **Study duration (week)** | 24 | | 52 | | | 52 | | |
| **N** | 1402 | | 793 | | | 782 | | |
| **Author** | **Garg 2017** | | **Buse**  **2018** | | | **Danne**  **2018** | | |

| **Supplementary Table 1 panel B. Risk of Bias of included randomized controlled trials** | **Other:**  **sponsorship bias** | Low risk.  Low risk  Industry funded but no high risk of bias feature encountered* | High risk.  Item G present* | Low risk.  No industry funding of the trial disclosed |
| --- | --- | --- | --- | --- |
|  | **Selective reporting** | Low risk.  Prespecified outcomes available on a clinical trial database and all reported in publication | Low risk.  Prespecified outcomes available on a clinical trial database and all reported in publication | Low risk.  Prespecified outcomes available on a clinical trial database and all reported in publication |
|  | **Incomplete outcome data** | Low risk.  Low dropout rate: 11% | Low risk.  Dropout rate: 0% | Low risk.  Dropout rate: 13% |
|  | **Blinding pf outcome assesment** | Low risk.  Quadruple  masking (Participant, Care Provider, Investigator, Sponsor) | High risk.  Single blind (participants) | Low risk.  Quadruple  masking |
|  | **Blinding of participants and personnel** | Low risk.  Quadruple  masking (Participant, Care Provider, Investigator, Sponsor) | High risk.  Single blind (participants) | Low risk.  Quadruple  masking (participant, care provider, investigator, outcome assessor) |
|  | **Allocation concealment** | Low risk.  Central allocation,  web-based randomization | High risk.  Single blind (participants) | Low risk.  Central allocation,  web-based randomization |
|  | **Random sequence generation** | Low risk.  Computer generated list | Unclear risk.  Randomization procedure not detailed | Low risk.  Computer generated list |
|  | **Author** | **Henry 2015 (DAPA)** | **Watada 2018** | **Kuhadya 2016** |

| **Panel B(continued). Risk of Bias of included randomized controlled trials** | **Other: sponsorship bias** | Low risk  Industry funded but no high risk of bias feature encountered* | Low risk  Industry funded but no high risk of bias feature encountered* | Low risk  Industry funded but no high risk of bias feature encountered* |
| --- | --- | --- | --- | --- |
|  | **Selective reporting** | Low risk  Prespecified outcomes available on a clinical trial database and all reported in publication | Low risk  Prespecified outcomes available on a clinical trial database and all reported in publication | Low risk  Prespecified outcomes available on a clinical trial database and all reported in publication |
|  | **Incomplete outcome data** | Low risk  Low dropout rate: 9.1% | Low risk  Low dropout rate:  Missing observations at EOT imputed as nonresponse. | Low risk  Low dropout rate: 2.3% |
|  | **Blinding pf outcome assesment** | Low risk  Quadruple  masking | Low risk  Quadruple  masking | Low risk  Quadruple  masking |
|  | **Blinding of participants and èpersonnel** | Low risk  Quadruple  masking (Participant, Care Provider, Investigator, Outcomes Assessor) | Low risk  Quadruple  Masking | Low risk  Quadruple  Masking |
|  | **Allocation concealment** | Low risk  Central allocation, web-based randomization | Low risk  Central allocation, web-based randomization | Low risk  Central allocation, web-based randomization |
|  | **Random sequence generation** | Low risk.  Computer generated list | Low risk.  Computer generated list | Low risk.  Computer generated list |
|  | **Author** | **Dandona 2017** | **Mathieu 2018** | **Kaku 2019** |

| **Panel B(continued). Risk of Bias of included randomized controlled trials** | **Other: sponsorship bias** | Low risk  Industry funded but no high risk of bias feature encountered* | Low risk  Industry funded but no high risk of bias feature encountered* | Low risk  Industry funded but no high risk of bias feature encountered* |
| --- | --- | --- | --- | --- |
|  | **Selective reporting** | Low risk  Prespecified outcomes available on a clinical trial database and all reported in publication | Low risk  Prespecified outcomes available on a clinical trial database and all reported in publication | Low risk  Prespecified outcomes available on a clinical trial database and all reported in publication |
|  | **Incomplete outcome data** | Low risk  Low dropout rate: 6%  Missing observations at EOT imputed as nonresponse. | Low risk  Low dropout rate: 6% | Low risk  Dropout rate:: 0% |
|  | **Blinding pf outcome assesment** | Low risk  Quadruple  masking | Low risk  Double  Masking  Matching placebo capsule | Low risk  Double blind (triple-dummy technique) |
|  | **Blinding of participants and èpersonnel** | Low risk  Quadruple  masking (Participant, Care Provider, Investigator, Outcomes Assessor) | Low risk  Double  Masking | Low risk  Double blind (triple-dummy technique) |
|  | **Allocation concealment** | Low risk  Central allocation, web-based randomization | Low risk  Central allocation, web-based randomization | Low risk  Central, computer-based allocation via IxVRS |
|  | **Random sequence generation** | Low risk.  Computer generated list | Low risk.  Computer generated schedule | Low risk.  Computer-based IxVRS |
|  | **Author** | **Kaku (a) 2019** | **Henry 2015 (CANA)** | **Pieber 2015** |

| **Panel B(continued). Risk of Bias of included randomized controlled trials** | **Other: sponsorship bias** | Low risk  Industry funded but no high risk of bias feature encountered* | Low risk  Industry funded but no high risk of bias feature encountered* | High risk  Item G present |
| --- | --- | --- | --- | --- |
|  | **Selective reporting** | Low risk  Prespecified outcomes available on a clinical trial database and all reported in publication | Low risk  Prespecified outcomes available on a clinical trial database and all reported in publication | Low risk  Prespecified outcomes available on a clinical trial database and all reported in publication |
|  | **Incomplete outcome data** | Low risk  Low dropout rate: 9%  Missing observations at EOT imputed as nonresponse. | Low risk  Low dropout rate: 6% | Low risk  Low dropout rate:  Missing observations at EOT imputed as nonresponse. |
|  | **Blinding pf outcome assesment** | Low risk  Double blind (triple-dummy technique) | Low risk  Double blind (triple-dummy technique) | Low risk  Quadruple  masking |
|  | **Blinding of participants and èpersonnel** | Low risk  Double blind (triple-dummy technique) | Low risk  Double blind (triple-dummy technique) | Low risk  Quadruple  Masking |
|  | **Allocation concealment** | Low risk  Double blind (triple-dummy technique) | Low risk  Double blind (triple-dummy technique) | Low risk  Central allocation, web-based randomization |
|  | **Random sequence generation** | Low risk.  Computer generated | Low risk.  Computer generated | Low risk.  Computer generated list |
|  | **Author** | **Rosenstock 2018 (EASE-2)** | **Rosenstock 2018 (EASE-3)** | **Shimada 2018** |

| **Supplementary Table 1 panel BRisk of Bias of included randomized controlled trials** | **Other:**  **sponsorship bias** | Low risk.  The Robert and Janice McNair Foundation funded the study | Low risk.  JDRF funded the study | Low risk.  Industry funded but no high risk of bias feature encountered* |
| --- | --- | --- | --- | --- |
|  | **Selective reporting** | Low risk.  Prespecified outcomes available on a clinical trial database and all reported in publication | Low risk.  Prespecified outcomes available on a clinical trial database and all reported in publication | Low risk.  Prespecified outcomes available on a clinical trial database and all reported in publication |
|  | **Incomplete outcome data** | Low risk.  No patients dropped out | Low risk.  Low dropout rate: | Low risk.  Low dropout rate: |
|  | **Blinding pf outcome assesment** | Low risk.  Quadruple  masking (Participant, Care Provider, Investigator, Outcomes Assessors) | Low risk.  Quadruple  masking | Low risk.  Quadruple  masking |
|  | **Blinding of participants and èpersonnel** | Low risk.  Quadruple  masking (Participant, Care Provider, Investigator, Outcomes Assessor) | Low risk.  Quadruple  masking | Low risk.  Quadruple  masking |
|  | **Allocation concealment** | Low risk.  Central allocation,  web-based randomization | Low risk.  Central allocation,  web-based randomization | Low risk.  Central allocation,  web-based randomization |
|  | **Random sequence generation** | Low risk.  Computer generated list | Low risk.  Computer generated list | Low risk.  Computer generated list |
|  | **Author** | **Sands 2015** | **Bode 2017** | **Baker 2017** |

| **Panel B(continued). Risk of Bias of included randomized controlled trials** | **Other: sponsorship bias** | Low risk  Industry funded but no high risk of bias feature encountered* | Low risk  Industry funded but no high risk of bias feature encountered* | Low risk  Industry funded but no high risk of bias feature encountered* |
| --- | --- | --- | --- | --- |
|  | **Selective reporting** | Low risk  Prespecified outcomes available on a clinical trial database and all reported in publication | Low risk  Prespecified outcomes available on a clinical trial database and all reported in publication | Low risk  Prespecified outcomes available on a clinical trial database and all reported in publication |
|  | **Incomplete outcome data** | Low risk  Low dropout rate:  Missing observations at EOT imputed as nonresponse. | Low risk  Low dropout rate:  Missing observations at EOT imputed as nonresponse. | Low risk  Low dropout rate:  Missing observations at EOT imputed as nonresponse. |
|  | **Blinding pf outcome assesment** | Low risk  Quadruple  masking | Low risk  Quadruple  masking | Low risk  Quadruple  masking |
|  | **Blinding of participants and èpersonnel** | Low risk  Quadruple  masking (Participant, Care Provider, Investigator, Outcomes Assessor) | Low risk  Quadruple  Masking | Low risk  Quadruple  Masking |
|  | **Allocation concealment** | Low risk  Central allocation, web-based randomization | Low risk  Central allocation, web-based randomization | Low risk  Central allocation, web-based randomization |
|  | **Random sequence generation** | Low risk.  Computer generated list | Low risk.  Computer generated list | Low risk.  Computer generated list |
|  | **Author** | **Garg 2017** | **Buse 2018** | **Danne**  **2018** |

**Abbreviations**: eGFR: estimated glomerular filtration rate;JDRF: Juvenile Diabetes Research Foundation; Sota: sotagliflozin; TID: total insulin dose; IxVRS: Interactive Voice/Web Response System;

**^a^**Insulin dose optimization during the 6 weeks preceding randomization(target: FPG 80-130 mg/dL and 2hr-PPG<180 mg/dL)

***Assessment of sponsorship bias**: in the presence of industry sponsorship, the following list of 8 items in trial designing, conducting or reporting, empirically linked by existing literature to biased outcomes in industry-funded trials and not captured by the Cochrane Risk of Bias domains, were assessed: if any one item was present, the trial was downgraded to “high risk of bias”.

**Item a**:unclearclinical relevance of outcome measures, i.e., the clinical relevance of trial outcomes is not supported by international guidelines (American Association for the study of Diabetes-ADA or European Association for the Study of Diabetes-EASD guidelines).

**Item b**: if active comparator was used: inadequacy of doses timing or way of administration,

**Item c**: -deviation from study protocol or original protocol changes or amendments after trial initiation

**Item d**: post-hoc selection of the major findings and endpoints

**Item e**: use of last observation carried forward analysis to impute missing data

**Item f**:on-treatment outcome reporting /absence of data and safety monitoring board

**Item g**:absence of sponsor-independent data analysis

**Item h**: early trial termination before the endpoint recorded on clinical trial registries

**S1 Table B**. Results of subgroup analysis.

| **Diabetes duration** | | |
| --- | --- | --- |
| **Outcome** | **diabetes duration < 20 y** | **diabetes duration ≥ 20 yr** |
| **DKA** | 2.46(1.29, 4.66) I2=0%, p=0.006, N=17, 2067 participants | 3.01 (1.94, 4.66), I^2^=0%, p<0.00001, N=21, 5329 participants |
| **HbA1c(%)** | -0.33 (-0.40, -0.26), I^2^=0%, p<0.00001, N =10 comparisons, 2099 participants | -0.38(-0.42, -0.33), I^2^=0%, p<0.00001,  N =10 comparisons, 5144 participants |
| **FPG(mg/dL)** | -19.27 (-26.29, -12.24), I^2^=0%, p=0.005, N=17, 2067 participants | -18.80 (-22.46, -15.13), I^2^=5%, p<0.00001, N=21, 5329 participants |
| **Time-in-**  **Range (%)** | 9.89(8.30,11.47) I^2^=0%, p<0.00001, N=14, 1210 participants | 9.32(7.44, 11.19) I^2^=32%, p<0.00001, N=17, 1840 participants |
| **MAGE(mg/dL)** | -12.53(-16.13,-8.94) I^2^=0%, p<0.00001, N=14, 1210 participants | -17.52(-20.01,-15.03) I^2^=0%, p<0.00001, N=17, 1840 participants |
| **Total insulin**  **dose (IU/d)** | -11.78(-14.55, -9.01), I^2^=35%, p<0.00001, N=17, 2067 participants | -10.38(-11.51, -9.26), I^2^=0%, p<0.00001, N=21, 5329 participants |
| **Basalinsulin dose**  **(IU/d)** | -13.27(-17.76, -8.78), I^2^=0%, p<0.00001, N=17, 2067 participants | -11.97(-13.68, -10.27]) I^2^=23%, p<0.00001,N=21, 5329 participants |
| **Bolus insulin dose**  **(IU/d)** | -9.83 (-11.99, -7.67)I^2^=0%, p<0.00001, N=17, 2067 participants | -9.73(-12.06, -7.41), I^2^=33%, p<0.00001, N=21, 5329 participants |
| **eGDR change (%)** | 10.62(9.46, 11.77), I^2^=0%, p<0.00001, N=17, 2067 participants | 11.43(10.15, 12.72), I^2^=33%, p<0.00001, N=21, 5329 participants |
| **BMIchange (%)** | -2.76(-3.38, -2.14), I^2^=30%, p<0.00001, N=17, 2067 participants | -3.43(-3.83, -3.02), I^2^=32%, p<0.00001, N=21, 5329 participants |
| **SystolicBP(mmHg)** | -3.48(-4.88, -2.07), I^2^=0%, p<0.00001, N=17, 2067 participants | -3.91(-4.70, -3.13), I^2^=0%, p<0.00001, N=21, 5329 participants |
| **eGFR**  **(ml/min/1.73 m^2^)** | -0.99(-1.89, -0.08), I^2^=0%, p=0.03, N=17, 2067 participants | -0.68(-1.31, -0.05), I^2^=0%, p=0.03, N=21, 5329 participants |
| **Albumin-creatinine**  **ratio (ACR)(mg/g)** | -13.69 (-24.33, -3.06), I^2^=0%, p=0.01, N=5, 807 participants | -7.80 (-15.73, -0.13), I^2^=0%, p=0.04, N=5, 2245 participants |
| **Hypoglycemia** | 0.82(0.22, 1.42), I^2^=0%, p=0.41, N=17, 2067 participants | 0.91(0..35, 1.69), I^2^=0%, p=0.78, N=21, 5329 participants |
| **Severe hypoglycemia** | 0.69(0.38, 1.28), I^2^=0%, p=0.24, N=17, 2067 participants | 0.92(0.60, 1.39), I^2^=0%, p=0.68, N=21, 5329 participants |
| **UTI** | 0.90(0.77,1.21]I^2^=0%, p=0.76, N=17, 2067 participants | 1.08(0.72, 1.63), I^2^=0%, p<0.00001, N=21, 5329 participants |
| **GTI** | 3.83(2.19, 6.70), I^2^=0%, p<0.00001, N=17, 2067 participants | 3.11(2.20, 4.39), I^2^=0%, p<0.00001, N=21, 5329 participants |
| **Volume depletion**  **events** | 1.49(0.78, 2.87), I^2^=0%, p=0.13, N=17, 2067 participants | 1.56(0.95, 2.56), I^2^=0%, p=0.08, N=21, 5329 participants |
| **Eye disorders** | 0.46(0.08, 2.57), I^2^=0%, p=0.29, N=17, 2067 participants | 0.22(0.08, 0.65), I^2^=0%, p=0.006, N=21, 5329 participants |
| **MACE** | 1.39(0.40, 4.85), I^2^=0%, p=0.71, N=17, 2067 participants | 0.95(0.42, 2.11), I^2^=0%, p=0.89, N=21, 5329 participants |
| **Baseline HbA1c levels** | | |
| **Outcome** | **baseline HbA1c levels < 8%** | **baseline HbA1c levels ≥8%** |
| **DKA** | 3.67(1.73, 7.77),I^2^=0%, p=0.0007, N=14, 2119 participants | 2.26(1.46, 3.49), I^2^=0%, p=0.0003, N=25, 5290 participants |
| **HbA1c (%)** | -0.32 (-0.38, -0.25), I^2^=4%, p<0.00001, N =11 comparisons, 2064 participants | -0.40(-0.45, -0.35), I^2^=0%, p<0.00001,  N =18 comparisons, 5179 participants |
| **FPG(mg/dL)** | -13.83(-19.08, -8.59), I^2^=0%, p<0.00001, N=14, 2119 participants | -23.04 (-29.32, -16.76), I2=25%, p<0.00001, N=25, 5290 participants |
| **Time-in-**  **Range (%)** | 10.28(7.86, 12.70), I^2^=0%, p<0.00001, N=11, 550 participants | 9.59(8.23, 10.95), I^2^=20%, p<0.00001, N=21, 2513 participants |
| **MAGE(mg/dL)** | -19.38(-25.11, -13.64),I^2^=0%, p<0.00001, N=11, 550 participants | -15.21 (-18.42, -12.01), I^2^=0%, p<0.00001, N=21, 2513 participants |
| **Total insulin**  **dose (IU/d)** | -10.71(-13.63, -7.79), I^2^=40%, p<0.00001, N=14, 2119 participants | -11.08 (-12.91, -9.25), I2=36%, p<0.00001, N=25, 5290 participants |
| **Basal insulin dose**  **(IU/d)** | -10.89(-14.39, -7.39), I^2^=34%, p<0.00001, N=14, 2119 participants | -13.12(-15.04, -11.21), I^2^=27%, p<0.00001, N=25, 5290 participants |
| **Bolus insulin dose**  **(IU/d)** | -10.18(-13.88, -6.48), I^2^=41%, p<0.00001, N=14, 2119 participants | -9.93(-11.57, -8.30), I2=24%, p<0.00001, N=25, 5290 participants |
| **eGDR change (%)** | 11.46(10.22, 12.71), I^2^=20%, p<0.00001, N=14, 2119 participants | 10.89 (9.68, 12.09), I^2^=30%, p<0.00001, N=25, 5290 participants |
| **BMI change (%)** | -3.42(-3.99, -2.84, I^2^=31%, p<0.00001, N=14, 2119 participants | -3.05(-3.49, -2.62), I^2^=41%, p<0.00001, N=25, 5290 participants |
| **Systolic BP (mmHg)** | -3.81(-5.07, -2.55), I^2^=0%, p<0.00001, N=14, 2119 participants | -3.81(-4.62, -3.00), I^2^=0%, p<0.00001, N=25, 5290 participants |
| **eGFR**  **(ml/min/1.73 m2)** | -1.07(-2.06, -0.09), I^2^=0%, p=0.03, N=14, 2119 participants | -0.67(-1.27, -0.06), I^2^=0%, p=0.03, N=25, 5290 participants |
| **Albumin-creatinine**  **ratio (ACR)(mg/g)** | -11.49(-22.78, -0.79), I^2^=0%, p=0.04, N=4, 1575 participants | -9.54(-16.55, -2.53), I^2^=0%, p=0.008, N=4, 1477 participants |
| **Hypoglycemia** | 0.92(0.64, 1.33),, I^2^=0%, p=0.65, N=14, 2119 participants | 0.94(0.70, 1.26), I^2^=0%, p=0.89, N=25, 5290 participants |
| **Severe**  **hypoglycemia** | 0.75(0.51, 1.10), I^2^=0%, p=0.14, N=14, 2119 participants | 0.89(0.58, 1.35), I^2^=0%, p=0.58, N=25, 5290 participants |
| **UTI** | 1.04(0.71, 1.55), I^2^=0%, p=0.83, N=14, 2119 participants | 0.97(0.78, 1.22), I^2^=0%, p=0.81, N=25, 5290 participants |
| **GTI** | 2.95(1.86, 4.66), I^2^=0%, p<0.00001, N=14, 2119 participants | 3.28(2.46, 4.38), I^2^=0%, p<0.00001, N=25, 5290 participants |
| **Volume depletion**  **events** | 1.89 (1.03, 3.95) I^2^=3%, p=0.04, N=14, 2119 participants | 1.48 (1.06, 1.92), I^2^=0%, p=0.04, N=25, 5290 participants |
| **Eye disorders** | 0.32(0.08, 0.96), I^2^=0%, p=0.04, N=14, 2119 participants | 0.24(0.07, 0.80), I^2^=0%, p=0.02, N=25, 5290 participants |
| **MACE** | 0.89(0.33, 2.44), I^2^=0%, p=0.94, N=25, 5290 participants | 1.02(0.43, 2.40), I^2^=0%, p=0.91, N=25, 5290 participants |
| **Baseline BMI** | | |
| **Outcome** | **baseline BMI >27 kg/m^2^** | **baseline BMI ≤27 kg/m^2^** |
| **DKA** | 3.95 (2.40, 6.50), I^2^=0%, p<0.00001, N=19, 6156 participants | 1.09 (0.38, 3.08),, I^2^=0%, p=0.87, N=19, 1240 participants |
| **HbA1c (%)** | -0.36(-0.41, -0.31),I^2^=8%,P<0.00001, N =19 comparisons, 6156 participants | -0.33(-0.42, -0.24),   \|  \| \| --- \|   I^2^=0%, p=0.0001, N =10 comparisons, 1087 participants |
| **FPG(mg/dL)** | -19.25 (-22.91, -15.60), I^2^=10%, p<0.0001, N=19, 6156 participants | -18.37(-25.85, -10.89), I^2^=0%, p<0.00001, N=19, 1240 participants |
| **Time-in-**  **Range (%)** | 10.50 (9.49, 11.52) I^2^=0%, p<0.00001, N=16, 2007 participants | 9.98(5.25, 14.51) I^2^=0%, p<0.00001, N=15, 1043 participants |
| **MAGE(mg/dL)** | -15.33(-18.13,-12.52)I^2^=0%, p<0.00001, N=16, 2007 participants | -18.24 (-23.54, -12.94) I^2^=0%, p<0.00001, N=15, 1043 participants |
| **Total insulin**  **dose (IU/d)** | -10.05(-11.12, -8.98), I^2^=0%, p<0.00001, N=19, 6156 participants | -15.62 (-19.37, -11.87),, I^2^=0%, p<0.00001, N=19, 1240 participants |
| **Basal insulin dose**  **(IU/d)** | -11.41 [-13.13, -9.69]  , I^2^=32%, p<0.00001, N=19, 6156 participants | -21.10(-26.71, -15.49),  I^2^=0%, p<0.00001, N=19, 1240 participants |
| **Bolus insulin dose**  **(IU/d)** | -9.09 [-11.00, -7.17]I^2^=30%, p<0.00001, N=19, 6156 participants | -15.43(-24.33, -6.52),, I2=27%, p=0.0007, N=19, 1240 participants |
| **eGDR change (%)** | 11.78 (10.72, 12.85), I^2^=39%, p<0.00001, N=19, 6156 participants | 9.20 (7.68, 10.73),I^2^=0%, p<0.00001, N=19, 1240 participants |
| **BMI change (%)** | -3.62(-3.88, -3.36), I^2^=0%, p=0.0004, p<0.0001, N=19, 6156 participants | \| -2.27(-3.05, -1.49), \| \| --- \|   I^2^=27%, p<0.00001, N=19, 1240 participants |
| **Systolic BP (mmHg)** | -3.97(-4.74, -3.21), I^2^=0%, p<0.00001, N=19, 6156 participants | -3.13 (-5.31, -0.95), I^2^=0%, p=0.0005, N=19, 1240 participants |
| **eGFR**  **(ml/min/1.73 m2)** | -0.71(-1.28, -0.14), I^2^=0%, p=0.01, N=19, 6156 participants | -1.07(-2.28, 0.14), I^2^=0%, p=0.08, N=19, 1240 participants |
| **Albumin-creatinine**  **ratio (ACR)(mg/g)** | -14.75(-26.70, -2.80), I^2^=0%, p=0.02,  N=19, 6156 participants | -7.99(-15.50, -0.48)  , I^2^=0%, p=0.04, N=3, 75 participants |
| **Hypoglycemia** | 0.99(0.60, -1.39), I^2^=0%, p=0.81, N=19, 6156 participants | 0.92(0.71, 1.24), I^2^=0%, p=0.81, N=19, 1240 participants |
| **Severe**  **hypoglycemia** | 0.84(0.62, 1.13), I^2^=0%, p=0.25, N=19, 6156 participants | 0.55(0.21, 1.44), I2=0%, p=0.22, N=19, 1240 participants |
| **UTI** | 1.06(0.86, 1.31), I^2^=0%, p=0.59, N=19, 6156 participants | 0.68(0.41, 1.13), I^2^=0%, p=0.14, N=19, 1240 participants |
| **GTI** | 3.28 (2.52, 4.26), I^2^=0%, p<0.00001, N=19, 6156 participants | 2.60(1.31, 5.16), I^2^=0%, p=0.001, N=19, 1240 participants |
| **Volume depletion**  **events** | 1.93(1.17, 3.17), I^2^=9%, p=0.010, N=19, 6156 participants | 1.03(0.54, 1.98), I^2^=0%, p=0.68, N=19, 1240 participants |
| **Eye disorders** | 0.25(0.09, 0.67), I^2^=0%, p=0.006, N=19, 6156 participants | 0.42 (0.15, 0.69), I^2^=0%, p=0.02, N=19, 1240 participants |
| **MACE** | 1.02 (0.50, 2.07), I^2^=0%, p=0.91, N=19, 6156 participants | 1.50(0.17, 7.43), I^2^=0%, p=0.99, N=19, 1240 participants |
| **Baseline eGDR** | | |
| **Outcome** | **baseline eGDR<8.3 mg/kg/min** | **baseline eGDR≥8.3 mg/kg/min** |
| **DKA** | 3.87(2.40, 6.23),I^2^=0%, p<0.00001, N=18, 4872 participants | 1.38(0.81, 2.36), I^2^=0%, p=0.36, N=20, 2524 participants |
| **HbA1c (%)** | -0.37 (-0.42, -0.32), I^2^=4%, p<0.00001, N =18comparisons, 5498 participants | -0.34(-0.41, -0.27), I^2^=0%, p<0.00001,  N =11 comparisons, 1771 participants |
| **FPG(mg/dL)** | -16.74 (-28.49, -5.00), I^2^=10%, p<0.00001, N=18, 4872 participants | -22.40 (-31.21, -13.58), I^2^=0%, p<0.00001, N=20, 2524 participants |
| **Time-in-**  **Range (%)** | 10.61(9.46, 11.76),  I^2^=0%, p<0.00001, N=18, 2066 participants | 8.52 (6.74, 10.29), I^2^=0%, p<0.00001, N=17, 1057 participants |
| **MAGE(mg/dL)** | -15.30(-10.75,-20.87) I^2^=0%, p<0.00001, N=18, 2066 participants | -17.74(-21.84, -13.64), I^2^=0%, p<0.00001, N=17, 1057 participants |
| **Total insulin**  **dose (IU/d)** | -10.08 (-11.23, -8.94), I^2^=0%, p<0.00001, N=18, 4872 participants | -12.20(-15.55, -8.85), I^2^=33%, p<0.00001, N=20, 2524 participants |
| **Basal insulin dose**  **(IU/d)** | -11.52 (-13.42, -9.63)  , I^2^=4%, p<0.00001, N=18, 4872 participants | -15.24(-20.69, -9.80),  I^2^=39%, p<0.00001, N=20, 2524 participants |
| **Bolus insulin dose**  **(IU/d)** | -9.16(-11.21, -7.11), I^2^=19%, p<0.00001, N=18, 4872 participants | -11.26 (-13.84, -8.69), I^2^=0%, p<0.00001, N=20, 2524 participants |
| **eGDR change (%)** | 12.53(11.79, 13.26), I^2^=3%, p<0.00001, N=18, 4872 participants | 10.34(9.11, 11.56), I^2^=0%, p<0.00001, N=20, 2524 participants |
| **BMI change (%)** | -3.52(-3.78, -3.26),, I^2^=0%, p<0.00001, N=18, 4872 participants | -3.16(-3.53, -2.79), I^2^=0%, p<0.00001, p<0.00001, N=20, 2524 participants |
| **Systolic BP (mmHg)** | -3.98(-4.75, -3.21), I^2^=0%, p<0.00001, N=18, 4872 participants | -2.94(-4.76, -1.12), I^2^=0%, p<0.00001, N=20, 2524 participants |
| **eGFR**  **(ml/min/1.73 m2)** | -0.69(-1.29, -0.09),, I^2^=0%, p=0.02, N=18, 4872 participants | -1.06(-2.06, -0.06), I^2^=0%, p=0.04, p<0.00001, N=20, 2524 participants |
| **Albumin-creatinine**  **ratio (ACR)(mg/g)** | -9.53(-18.44, -1.62), I^2^=0%, p=0.01, N=6, 3003 participants | -7.66 (-15.17, -0.15), I^2^=0%, p=0.04, N=3, 75 participants |
| **Hypoglycemia** | 0.84(0.67, 1.05), I^2^=0%, p=0.81, N=18, 4872 participants | 0.92(0.69, 1.23), I^2^=0%, p=0.58, N=20, 2524 participants |
| **Severe**  **hypoglycemia** | 0.87(0.65, 1.16), I^2^=0%, p=0.64, N=18, 4872 participants | 0.54(0.24, 1.20), I^2^=0%, p=0.13, N=20, 2524 participants |
| **UTI** | 1.03(0.83, 1.29),I^2^=0%, p=0.94, N=18, 4872 participants | 0.97(0.67, 1.42), I^2^=0%, p=0.94, p<0.00001, N=20, 2524 participants |
| **GTI** | 3.20(2.44, 4.21), I^2^=0%, p<0.00001, N=18, 4872 participants | 3.67(2.17, 6.21), I^2^=0%, p<0.00001, p<0.00001, N=20, 2524 participants |
| **Volume depletion**  **events** | 1.97(1.24, 3.12), I^2^=0%, p=0.004, N=18, 4872 participants | 1.16(0.65, 2.07), I^2^=0%, p=0.68, N=20, 2524 participants |
| **Eye disorders** | 0.20(0.07, 0.60), I^2^=0%, p<0.00001, N=18, 4872 participants | 0.51(0.14, 0.88)  , I^2^=0%, p=0.04, N=20, 2524 participants |
| **MACE** | 0.79(0.42, 1.47), I^2^=0%, p<0.00001, N=18, 4872 participants | 1.00(0.19, 5.28), I^2^=0%, p=0.98, N=20, 2524 participants |
| **Baseline renal function stage** | | |
| **Outcome** | **eGFR≥60 ml/min/1.73 m^2^** | **eGFR<60 ml/min/1.73m^2^** |
| **DKA** | 2.45 [1.19, 5.07],I^2^=0%, p=0.01, N=26, 2622 participants | 3.60 [2.03, 6.38], I^2^=0%, p=0.0001, N=12, 4774 participants |
| **HbA1c (%)** | -0.34 [-0.41, -0.28], I^2^=0%, p<0.00001, N=17 comparisons, 2469 participants | -0.37 [-0.44, -0.29] I^2^=34%, p<0.00001,  N =12 comparisons, 4774 participants |
| **FPG(mg/dL)** | -18.87 [-23.79, -13.94], I^2^=0%, p<0.00001, N=26, 2622 participants | -19.09 [-23.65, -14.53], I^2^=20%, p<0.00001, N=12, 4774 participants |
| **Time-in-**  **Range (%)** | 9.23 [7.90, 10.56] I^2^=0%, p<0.00001, N=20, 1818 participants | 10.58 [8.73, 12.42], I^2^=26%, p<0.00001, N=11, 1232 participants |
| **MAGE(mg/dL)** | -14.93 [-18.00, -11.86] I^2^=35%, p<0.00001, N=20, 1818 participants | -16.69 [-19.43, -13.94] I^2^=0%, p<0.00001, N=11, 1232 participants |
| **Total insulin**  **dose (IU/d)** | -12.02 [-13.67, -10.37], I^2^=0%, p<0.00001, N=26, 2622 participants | -9.64 [-10.93, -8.34], I^2^=14%, p<0.00001, N=12, 4774 participants |
| **Basal insulin dose**  **(IU/d)** | -15.63 [-18.16, -13.09], I^2^=17%, p<0.00001, N=26, 2622 participants | -10.16 [-11.68, -8.64],I^2^=1%, p<0.00001, N=12, 4774 participants |
| **Bolus insulin dose**  **(IU/d)** | -11.52 [-14.31, -8.74] I^2^=11%, p<0.00001, N=26, 2622 participants | -10.03 [-12.18, -7.88], I^2^=0%, p<0.00001, N=12, 4774 participants |
| **eGDR change (%)** | 10.12 [9.15, 11.10], I^2^=0%, p<0.00001, N=26, 2622 participants | 12.15 [10.64, 13.65], I^2^=34%, p<0.00001, N=12, 4774 participants |
| **BMI change (%)** | -2.78 [-3.33, -2.24], I^2^=0%, p<0.00001, N=26, 2622 participants | -3.56 [-3.95, -3.17], I^2^=30%, p<0.00001, N=12, 4774 participants |
| **Systolic BP (mmHg)** | -4.06 [-5.39, -2.73], I^2^=0%, p<0.00001, N=26, 2622 participants | -3.72 [-4.52, -2.92], I^2^=0%, p<0.00001, N=12, 4774 participants |
| **eGFR**  **(ml/min/1.73 m2)** | -1.09 [-1.91, -0.27], I^2^=0%, p=0.009, N=26, 2622 participants | -0.57 [-1.24, 0.10], I^2^=0%, p=0.09, N=12, 4774 participants |
| **Albumin-creatinine**  **ratio (ACR)(mg/g)** | -7.99 [-15.50, -0.48], I^2^=0%, p=0.04, N=3, 75 participants | -14.75 [-26.70, -2.80], I^2^=0%, p=0.02, N=5, 2977 participants |
| **Hypoglycemia** | 0.88(0.50, 1.28), I^2^=0%, p=0.81, N=26, 2622 participants | 0.91(0.45, 1.36), I^2^=0%, p=0.92, N=12, 4774 participants |
| **Severe**  **hypoglycemia** | 0.90 [0.43, 1.87], I^2^=0%, p=0.78, N=26, 2622 participants | 0.79 [0.58, 1.08], I^2^=0%, p=0.14, N=12, 4774 participants |
| **UTI** | 0.99 [0.70, 1.40], I^2^=0%, p=0.95, N=26, 2622 participants | 0.99 [0.78, 1.26], I^2^=0%, p=0.95, N=12, 4774 participants |
| **GTI** | 3.17 [2.01, 5.01], I^2^=0%, p<0.00001, N=26, 2622 participants | 3.19 [2.38, 4.26], I^2^=0%, p<0.00001, N=12, 4774 participants |
| **Volume depletion**  **events** | 1.62 (1.08, 2.16), I^2^=0%, p=0.04, N=26, 2622 participants | 1.92 [1.11, 3.33], I^2^=0%, p=0.02,  N=12, 4774 participants |
| **Eye disorders** | 0.46 [0.40, 0.86], I^2^=0%, p=0.04, N=26, 2622 participants | 0.19 [0.07, 0.55], I^2^=0%, p=0.002,  N=12, 4774 participants |
| **MACE** | 0.87 [0.23, 3.31], I^2^=0%, p=0.75, N=26, 2622 participants | 1.13 [0.52, 2.47], I^2^=0%, p=0.75, N=12, 4774 participants |
| **Study duration** | | |
| **Outcome** | **duration<24 weeks** | **duration≥24 weeks** |
| **DKA** | 1.95 [1.15, 3.43] I^2^=0%, p=0.01, N=23, 914 participants | 3.18 [1.96, 5.15], I^2^=0%, p<0.00001, N=15, 6482 participants |
| **HbA1c (%)** | -0.36 [-0.45, -0.27], I^2^=0%, p<0.00001, N=14 comparisons, 761 participants | -0.36 [-0.40, -0.31], I^2^=0%, p<0.00001,  N =15 comparisons, 6482 participants |
| **FPG(mg/dL)** | -14.83 [-21.10, -8.56], I^2^=0%, p<0.00001, N=23, 914 participants | -20.50 [-24.61, -16.39], I^2^=23%, p<0.00001, N=15, 6482 participants |
| **Time-in-**  **Range (%)** | 7.88 [5.31, 10.46] I^2^=0%, p<0.00001, N=18, 455 participants | 10.28 [8.99, 11.57] I^2^=24%, p<0.00001, N=13, 2595 participants |
| **MAGE(mg/dL)** | -16.37 [-21.74, -11.00] I^2^=0%, p<0.00001, N=18, 455 participants | -15.62 [-18.32, -12.92], I^2^=20%, p<0.00001, N=13, 2595 participants |
| **Total insulin**  **dose (IU/d)** | -11.36 [-14.15, -8.58], I^2^=3%, p=0.009, N=23, 914 participants | -10.46 [-11.71, -9.20], I^2^=31%, p<0.00001, N=15, 6482 participants |
| **Basal insulin dose**  **(IU/d)** | -13.07 [-16.75, -9.39], I^2^=14%, p<0.00001, N=23, 914 participants | -12.14 [-14.21, -10.08] I^2^=30%, p<0.00001, N=15, 6482 participants |
| **Bolus insulin dose**  **(IU/d)** | -12.24 [-16.76, -7.73],I^2^=43%, p<0.00001, N=23, 914 participants | -3.24 [-3.92, -2.56], I^2^=0%, p<0.00001, N=15, 6482 participants |
| **eGDR change (%)** | 9.44 [7.78, 11.09], I^2^=0%, p<0.00001, N=23, 914 participants | 11.61 [10.46, 12.76], I^2^=33%, p<0.00001, N=15, 6482 participants |
| **BMI change (%)** | -2.42 [-2.91, -1.92],I2=9%, p<0.00001, N=23, 914 participants | -3.66 [-4.01, -3.31], I^2^=43%, p<0.00001, N=15, 6482 participants |
| **Systolic BP (mmHg)** | -4.82 [-6.49, -3.15], I^2^=0%, p<0.00001, N=23, 914 participants | -3.60 [-4.36, -2.85], I^2^=0%, p<0.00001, N=15, 6482 participants |
| **eGFR**  **(ml/min/1.73 m2)** | -1.35(-1.49, -0.14), I^2^=0%, p=0.01, N=23, 914 participants | -0.50(-1.06, 0.05), I^2^=0%, p=0.16, N=15, 6482 participants |
| **Albumin-creatinine**  **ratio (ACR)(mg/g)** | -7.99 [-15.50, -0.48], I2=0%, p=0.04, N=3, 75 participants | -14.75 [-26.70, -2.80], I2=0%, p=0.02, N=5, 2977 participants |
| **Hypoglycemia** | 0.82(0.50, 1.12), I^2^=0%, p=0.88, N=23, 914 participants | 0..91(0.65, 1.56), I^2^=0%, p=0.52, N=15, 6482 participants |
| **Severe**  **hypoglycemia** | 1.09 [0.43, 2.78], I^2^=0%, p=0.86, N=23, 914 participants | 0.79 [0.58, 1.06],I^2^=0%, p=0.11, N=15, 6482 participants |
| **UTI** | 0.94 [0.43, 2.06], I^2^=0%, p=0.88, N=23, 914 participants | 0.99 [0.81, 1.22], I^2^=0%, p=0.96, N=15, 6482 participants |
| **GTI** | 1.95 [1.18, 3.13], I^2^=0%, p=0.02, N=23, 914 participants | 3.48 [2.69, 4.51], I^2^=0%, p<0.00001, N=15, 6482 participants |
| **Volume depletion**  **events** | 1.59 [1.04, 2.14], I^2^=0%, p=0.04, N=23, 914 participants | 1.75 [1.07, 2.85],I^2^=0%, p=0.03, N=15, 6482 participants |
| **Eye disorders** | 1.07 [0.05, 22.25], I^2^=0%, p=0.96, N=23, 914 participants | 0.24 [0.09, 0.62], I^2^=0%, p=0.003, N=15, 6482 participants |
| **MACE** | No events, I^2^=NA, N=23, 914 participants | 1.06 [0.54, 2.08], I^2^=0%, p=0.87, N=15, 6482 participants |
| **Background therapy** | | |
| **Outcome** | **no pre-randomization insulin optimization** | **pre-randomization insulin optimization** |
| **DKA** | 2.12 [1.22, 3.67] I^2^=0%, p=0.007, N=20, 2978 participants | 3.12 [1.84, 5.29], I^2^=0%, p<0.0001, N=18, 4418 participants |
| **HbA1c (%)** | -0.45 [-0.52, -0.38], I^2^=0%, p<0.00001, N =11 comparisons, 261 participants | -0.35[-0.40, -0.29], I^2^=0%, p<0.00001,  N=17 comparisons, 4418 participants |
| **FPG(mg/dL)** | -19.58 [-24.45, -14.71], I^2^=0%, p<0.00001, N=20, 2978 participants | -18.88 [-23.53, -14.23], I^2^=21%, p<0.00001, N=18, 4418 participants |
| **Time-in-**  **Range (%)** | 7.15 [4.62, 9.69],I^2^=2%, p<0.00001, N=13, 421 participants | 10.58 [9.52, 11.65] I^2^=0%, p<0.00001, N=18, 2629 participants |
| **MAGE(mg/dL)** | \| -12.24 [-18.13, -6.36] \| \| --- \|   I^2^=0%, p<0.00001, N=13, 421 participants | -16.41 [-18.85, -13.97] I^2^=9%, p<0.00001, N=18, 2629 participants |
| **Total insulin**  **dose (IU/d)** | -11.20 [-13.60, -8.79],I^2^=36%, p<0.00001, N=20, 2978 participants | -10.34 [-11.56, -9.12], I^2^=0%, p<0.00001, N=18, 4418 participants |
| **Basal insulin dose**  **(IU/d)** | -11.29 [-14.49, -8.09], I^2^=41%, p<0.00001, N=20, 2978 participants | -13.02 [-15.10, -10.94] I^2^=34%, p<0.00001, N=18, 4418 participants |
| **Bolus insulin dose**  **(IU/d)** | -10.77 [-13.33, -8.20],I^2^=0%, p<0.00001, N=20, 2978 participants | -9.87 [-12.38, -7.37] I^2^=40%, p<0.00001, N=18, 4418 participants |
| **eGDR change (%)** | 9.82 [8.19, 11.45], I^2^=22%, p<0.00001, N=20, 2978 participants | 11.67 [10.72, 12.63], I^2^=27%, p<0.00001, N=18, 4418 participants |
| **BMI change (%)** | -2.74 [-3.33, -2.16], I^2^=44%, p<0.00001, N=20, 2978 participants | -3.50 [-3.91, -3.09], I^2^=33%, p<0.00001, N=18, 4418 participants |
| **Systolic BP (mmHg)** | -3.75 [-4.65, -2.84] I^2^=0%, p<0.00001, N=20, 2978 participants | -3.89 [-4.94, -2.84], I^2^=0%, p<0.00001, N=18, 4418 participants |
| **eGFR**  **(ml/min/1.73 m2)** | -0.43 [-0.52, -0.08], I^2^=0%, p=0.04, N=20, 2978 participants | -1.01 [-1.68, -0.34], I^2^=0%, p=0.003, N=18, 4418 participants |
| **Albumin-creatinine**  **ratio (ACR)(mg/g)** | -20.10 [-39.73, -0.47] I^2^=NA, p=0.04, N=1, 1402 participants | -14.57(-26.87, -2.28), I2=0%, p=0.01, N=7, 1650 participants |
| **Hypoglycemia** | 0.85 [0.66, 1.10] I^2^=0%, p=0.23, N=20, 2978 participants | 0.94 [0.73, 1.19] I^2^=0%, p=0.60, N=18, 4418 participants |
| **Severe**  **hypoglycemia** | 0.96 [0.57, 1.60] I^2^=0%, p=0.86,  N=20, 2978 participants | 0.75 [0.53, 1.06], I^2^=0%, p=0.10, N=18, 4418 participants |
| **UTI** | 0.90 [0.62, 1.29], I^2^=0%, p=0.92, N=20, 2978 participants | 1.03 [0.82, 1.30], I^2^=0%, p=0.78, N=18, 4418 participants |
| **GTI** | 2.69 [1.75, 4.13],I^2^=0%, p<0.0001, N=20, 2978 participants | 3.45 [2.56, 4.65], I^2^=0%, p<0.00001, N=18, 4418 participants |
| **Volume depletion**  **events** | 1.46 [1.03, 2.01], I^2^=0%, p=0.04, N=20, 2978 participants | 1.60 [1.02, 2.18], I^2^=0%, p=0.04, N=18, 4418 participants |
| **Eye disorders** | 0.16 [0.03, 0.97], I^2^=0%, p=0.04, N=20, 2978 participants | 0.32 [0.12, 0.91], I^2^=0%, p=0.03, N=18, 4418 participants |
| **MACE** | 2.40 [0.42, 13.80] , I^2^=0%, p=0.33,  N=20, 2978 participants | 0.92 [0.44, 1.90], I^2^=0%, p=0.82, N=18, 4418 participants |

Abbreviations: AE: adverse events; FPG: fasting plasma glucose; MACE: major adverse cardiovascular outcomes DKA: diabetic ketoacidosis; GTI: genital tract infections; PPG: postprandial plasma glucose; UTI: urinary tract infections

**S1 Table C. Univariable meta-regression for moderators of the Risk Ratio of Diabetic Ketoacidosis (DKA).**

| **Univariable Meta-Regression: baseline predictors of incident DKA** | | | | | | |
| --- | --- | --- | --- | --- | --- | --- |
| **Patient-related factors** |  | | | | | |
| **Moderator** | **Coefficient** | **95%CI** | **SE** | **P** | **N-comparisons** | **N-participants** |
| **Age(yr)** | 0.096 | -0.005 0.198 | 0.052 | 0.063 | 38 | 7396 |
| **Gender(% M)** | -0.006 | -0.056 0.044 | 0.025 | 0.821 | 38 | 7396 |
| **Ethnicity:** |  | | | | | |
| **Caucasians (%)** | -0.005 | -0.055 0.045 | 0.026 | 0.842 | 38 | 7396 |
| **Asians (%)** | -0.017 | -0.031 0.003 | 0.007 | 0.129 | 38 | 7396 |
| **Hispanic (%)** | 0.006 | -0.051, 0.048 | 0.028 | 0.614 | 38 | 7396 |
| **Black (%)** | 0.140 | -0.019 0.299 | 0.081 | **0**.084 | 38 | 7396 |
| **CSI users (%)** | -1.710 | -5.731, 0.231 | 0.063 | 0.404 | 38 | 7396 |
| **Total ID(IU/d)** | 0.068 | 0.003, 0.133 | 0.033 | **0.040** | 38 | 7396 |
| **Diabetes duration(yr)** | 0.146 | -0.003, 0.295 | 0.076 | 0.061 | 38 | 7396 |
| **BMI(kg/m^2^)** | 0.459 | 0.241, 0.677 | 0.111 | **<0.00001** | 38 | 7396 |
| **HbA1c(%)** | -1.226 | -2.385, -0.068 | 0.591 | **0.038** | 38 | 7396 |
| **FPG(mg/dL)** | 0.009 | -0.023, 0.040 | 0.017 | 0.589 | 38 | 7396 |
| **eGDR(mg/kg/min)** | -1.028 | -1.600, -0.456 | 0.292 | **<0.00001** | 38 | 7396 |
| **Renal function stage** | -0.412 | -0.907, 0.084 | 0.253 | 0.103 | 38 | 7396 |
| **Fasting BHB(mmol/L)** | -2.446 | -7.918, 3.026 | 2.792 | 0.381 | 24 | 2538 |
| **Study design-related factors** |  | | | | | |
| **Study duration(wk)** | 0.018 | -0.005, 0.041 | 0.012 | 0.127 | 38 | 7396 |
| **Study sample size (N)** | 0.008 | -0.002, 0.015 |  | 0.121 | 38 | 7396 |
| **SGLT2 inhibitor dose** | 0.525 | -0.103, 1.149 | 0.318 | 0.122 | 38 | 7396 |
| **SGLT2 inhibitor drug:** |  | | | | | |
| **DAPA vs. others** | -0.380 | -1.275, 0.514 | 0.456 | 0.405 | 38 | 7396 |
| **EMPA vs. others** | -0.327 | -1.734, 1.079 | 0.717 | 0.648 | 38 | 7396 |
| **CANA vs. others** | -0.154 | -1.071, 0.763 | 0.468 | 0.742 | 38 | 7396 |
| **IPRA vs others** | -0.138 | -1.078, 0.802 | 0.480 | 0.774 | 38 | 7396 |
| **SOTA vs. others** | -1.798 | -3.753, 0.156 | 0.997 | 0.071 | 38 | 7396 |
| **Pre-randomization insulin optimization**  **(present vs. absent)** | 0.215 | -0.420, 0.850 | 0.324 | 0.397 | 38 | 7396 |
| **Risk-of-Bias**  **(high vs. low-unclear)** | 0.192 | -0.488, 0.872 | 0.347 | 0.782 | 38 | 7396 |
| **Univariable Meta-Regression: treatment-related predictors of DKA** | | | | | | |
| **Moderator** | **Coefficient** | **95%CI** | **SE** | **P** | **N-comparisons** | **N-participants** |
| **Total ID change (%)** | 0.064 | -0.029 0.157 | 0.048 | 0.278 | 38 | 7396 |
| **Basal ID change (%)** | 0.019 | -0.040 0.078 | 0.030 | 0.530 | 38 | 7396 |
| **Bolus ID change (%)** | 0.015 | -0.043, 0.073 | 0.030 | 0.605 | 38 | 7396 |
| **TID change (%)/baseline BMI ratio** | 0.029 | -0.002, 0.060 | 0.016 | 0.055 | 38 | 7396 |
| **TID change (%)/baseline RIS ratio (IU^2^/kg/d)** | -0.054 | -0.084, -0.024 | 0.015 | **0.0007** | 38 | 7396 |
| **Residual INS-SGLT2i effect (%)** | 0.006 | -0.047, 0.060 | 0.027 | 0.819 | 38 | 7396 |
| **BMI change (%)** | -0.642 | -1.007, -0.276 | 0.186 | **0.001** | 38 | 7396 |
| **HbA1 change(%)** | -1.844 | -6.287, 2.599 | 2.267 | 0.416 | 38 | 7396 |
| **FPG change(mg/dL)** | 0.043 | -0.009, 0.076 | 0.017 | 0.102 | 38 | 7396 |
| **Time-in-range(%) change** | 0.027 | -0.121, 0.174 | 0.076 | 0.801 | 31 | 3050 |
| **MAGE(mg/dL) change** | -0.003 | -0.049, 0.045 | 0.024 | 0.965 | 31 | 3050 |
| **eGDR change (%)** | 0.317 | 0.136, 0.499 | 0.093 | **0.0001** | 38 | 7396 |
| **RIS change (%)** | -6.299 | -12.130, -0.467 | 2.975 | **0.034** | 38 | 7396 |
| **BHB change (mmol/L)** | 0.001 | -0.001, 0.001 | 0.0002 | 0.613 | 24 | 2538 |
| **Volume depletion events** | 0.357 | 0.163, 0.551 | 0.099 | **<0.00001** | 38 | 7396 |
| **UTIs** | 0.307 | -0.186, 0.799 | 0.251 | 0.222 | 38 | 7396 |
| **GTIs** | -0.005 | -0.329, 0.318 | 0.165 | 0.974 | 38 | 7396 |
| **Respiratory infections** | 0.216 | -0.156, 0.616 | 0.104 | 0.214 | 38 | 7396 |
| **Severe hypoglycemia** | 0.273 | -0.244, 0.790 | 0.264 | 0.579 | 38 | 7396 |

**Abbreviations.** DKA: diabetic ketoacidosisFPG: fasting plasma glucose; ID: insulin dose, CIS: continuous subcutaneous infusion; eGDR: estimated Glucose Disposal rate; eGFR: estimated glomerular filtration rate; BHB: Beta-Hydroxybutyrate;;RIS: relative insulin sensitivity; SGLT2i: SGLT2 inhibitorGTI: genital tract infections;UTI: urinary tract infections

**S1 Table D. Univariable meta-regression for moderators of HbA1c changes(%)**

| **Univariable Meta-Regression: baseline predictors of HbA1c(%) changes** | | | | | | | |
| --- | --- | --- | --- | --- | --- | --- | --- |
| **Patient-related factors** | |  | | | | | |
| **Moderator** | | **Coefficient** | **95%CI** | **SE** | **P** | **N-comparisons** | **N-participants** |
| **Age(yr)** | | -0.003 | -0.015, 0.008 | 0.006 | 0.583 | 29 | 7243 |
| **Gender(% M)** | | 0.001 | -0.005, 0.008 | 0.003 | 0.654 | 29 | 7243 |
| **Ethnicity:** | |  | | | | | |
| **Caucasians (%)** | | 0.001 | -0.002 0.003 | 0.001 | 0.607 | 29 | 7243 |
| **Asians (%)** | | 0.001 | -0.001 0.002 | 0.001 | 0.538 | 29 | 7243 |
| **Hispanic (%)** | | 0.004 | -0.007, 0.011 | 0.003 | 0.689 | 29 | 7243 |
| **Black (%)** | | -0.013 | -0.032 0.005 | 0.009 | **0**.157 | 29 | 7243 |
| **CSI users (%)** | | 0.190 | -0.179, 0.559 | 0.188 | 0.313 | 29 | 7243 |
| **Total ID (IU/d)** | | -0.001 | -0.008, 0.008 | 0.004 | 0.936 | 29 | 7243 |
| Diabetes duration(yr) | | -0.002 | -0.017, 0.013 | 0.008 | 0.764 | 29 | 7243 |
| **BMI (kg/m^2^)** | | -0.012 | -0.040,0.016 | 0.014 | 0.402 | 29 | 7243 |
| **HbA1c(%)** | | -0.060 | -0.169, 0.050 | 0.056 | 0.288 | 29 | 7243 |
| **FPG(mg/dL)** | | -0.001 | -0.005 0.002 | 0.002 | 0.529 | 29 | 7243 |
| **eGDR(mg/kg/min)** | | 0.001 | -0.068, 0.069 | 0.035 | 0.981 | 29 | 7243 |
| **Renal function stage** | | -0.046 | -0.098, 0.006 | 0.026 | 0.081 | 29 | 7243 |
| **Fasting BHB(mmol/L)** | | 0.001 | -0.001, 0.001 | 0.001 | 0.915 | 29 | 7243 |
| **Study design-related factors** | |  | | | | | |
| **Study duration (wk)** | | 0.001 | -0.001, 0.004 | 0.001 | 0.251 | 29 | 7243 |
| **Study sample size (N)** | | -0.001 | -0.002, 0.001 | 0.001 | 0.154 | 29 | 7253 |
| **SGLT2 inhibitor dose** | | -0.083 | -0.140, -0.027 | 0.029 | **0.001** | 29 | 7243 |
| **SGLT2 inhibitor drug:** | |  | | | | | |
| **DAPA vs. others** | | -0.002 | -0.117, 0.113 | 0.059 | 0.972 | 29 | 7243 |
| **EMPA vs. others** | | -0.055 | -0.144, 0.034 | 0.045 | 0.225 | 29 | 7243 |
| **CANA vs. others** | | 0.102 | -0.091, 0.295 | 0.099 | 0.301 | 29 | 7243 |
| **IPRA vs others** | | 0.001 | -0.234, 0.236 | 0.120 | 0.993 | 29 | 7243 |
| **SOTA vs. others** | | 0.033 | -0.057, 0.123 | 0.046 | 0.477 | 29 | 7243 |
| **Pre-randomization insulin optimization**  **(present vs. absent)** | | 0.094 | 0.002, 0.186 | 0.047 | **0.044** | 29 | 7243 |
| **Risk-of-Bias**  **(high/unclear vs. low)** | | 0.131 | -0.412, 0.676 | 0.144 | 0.813 | 29 | 7243 |
| **Univariable Meta-Regression: treatment-related predictors of HbA1c (%) changes** | | | | | | | |
| **Moderator** | **Coefficient** | | **95%CI** | **SE** | **P** | **N-comparisons** | **N-participants** |
| **Total ID change (%)** | 0.012 | | -0.004, 0.029 | 0.009 | 0.150 | 29 | 7243 |
| **Basal ID change (%)** | 0.006 | | -0.004, 0.016 | 0.005 | 0.261 | 29 | 7243 |
| **Bolus ID change (%)** | 0.014 | | -0.042, 0.072 | 0.029 | 0.594 | 29 | 7243 |
| **TID change (%)/baseline BMI ratio** | 19.914 | | -34.857, 74.685 | 27.945 | 0.476 | 29 | 7243 |
| **TID change (%)/baseline RIS ratio (IU^2^/kg/d)** | -0.002 | | -0.007, 0.003 | 0.003 | 0.446 | 29 | 7243 |
| **Residual INS-SGLT2i effect(%)** | -0.006 | | -0.015, 0.002 | 0.004 | 0.144 | 29 | 7243 |
| **BMI change (%)** | 0.030 | | -0.023 0.083 | 0.027 | 0.267 | 29 | 7243 |
| **FPG change(mg/dL)** | 0.001 | | -0.003,0.005 | 0.002 | 0.566 | 29 | 7243 |
| **eGDR change (%)** | -0.024 | | -0.045 -0.002 | 0.010 | **0.031** | 29 | 7243 |
| **RIS change (%)** | -0.003 | | -0.009, 0.003 | 0.003 | 0.375 | 29 | 7243 |
| **BHB change (mmol/L)** | -0.434 | | -1.064, 0.196 | 0.321 | 0.177 | 29 | 7243 |
| **DKA** | 0.003 | | -0.012, 0.019 | 0.008 | 0.671 | 29 | 7243 |
| **Volume depletion events** | -0.017 | | -0.038, 0.003 | 0.011 | 0.100 | 29 | 7243 |
| **UTIs** | -0.019 | | -0.071 0.036 | 0.028 | 0.535 | 29 | 7243 |
| **GTIs** | -0.016 | | -0.072, 0.037 | 0.027 | 0.579 | 29 | 7243 |
| **Respiratory infections** | -0.018 | | -0.074, 0.039 | 0.129 | 0.619 | 29 | 7243 |

**Abbreviations.** DKA: diabetic ketoacidosis FPG: fasting plasma glucose; ID: insulin dose, CIS: continuous subcutaneous infusion; eGDR: estimated Glucose Disposal rate; eGFR: estimated glomerular filtration rate; BHB: Beta-Hydroxybutyrate;; RIS: relative insulin sensitivity; SGLT2i: SGLT2 inhibitor GTI: genital tract infections; UTI: urinary tract infections

**S1 Table E. Univariable meta-regression for moderators of BMI changes(%)**

| **Univariable Meta-Regression: baseline predictors of BMI changes(%)** | | | | | | |
| --- | --- | --- | --- | --- | --- | --- |
| **Patient-related factors** |  | | | | | |
| **Moderator** | **Coefficient** | **95%CI** | **SE** | **P** | **N-comparisons** | **N-participants** |
| **Age(yr)** | -0.059 | -0.137, 0.019 | 0.040 | 0.136 | 38 | 7396 |
| **Gender(% M)** | 0.005 | -0.030, 0.040 | 0.018 | 0.789 | 38 | 7396 |
| **Ethnicity:** |  | | | | | |
| **Caucasians (%)** | 0.013 | -0.002 0.027 | 0.007 | 0.081 | 38 | 7396 |
| **Asians (%)** | 0.008 | -0.003, 0.020 | 0.006 | 0.140 | 38 | 7396 |
| **Hispanic (%)** | -0.095 | -0.246, 0.056 | 0.077 | 0.214 | 38 | 7396 |
| **Black (%)** | -0.011 | -0.034 0.007 | 0.011 | **0**.394 | 38 | 7396 |
| **CSI users (%)** | 0.550 | -2.678, 3.777 | 1.647 | 0.739 | 38 | 7396 |
| **Total ID (IU/d)** | -0.060 | -0.117,-0.004 | 0.029 | **0.037** | 38 | 7396 |
| **Diabetes duration(yr)** | -0.077 | -0.191, 0.037 | 0.058 | 0.186 | 38 | 7396 |
| **BMI (kg/m^2^)** | -0.305 | -0.504,-0.107 | 0.101 | **0.003** | 38 | 7396 |
| **HbA1c(%)** | 0.531 | -0.367, 1.430 | 0.458 | 0.246 | 38 | 7396 |
| **FPG(mg/dL)** | -0.018 | -0.043, 0.007 | 0.013 | 0.168 | 38 | 7396 |
| **eGDR(mg/kg/min)** | 0.620 | -0.064, 1.177 | 0.284 | **0.029** | 38 | 7396 |
| **Renal function stage** | -0.354 | -0.784, 0.076 | 0.219 | 0.107 | 38 | 7396 |
| **Fasting BHB(mmol/L)** | 0.011 | -0.011, 0.011 | 0.010 | 0.713 | 24 | 2193 |
| **Study design-related factors** |  | | | | | |
| **Study duration (wk)** | -0.033 | -0.209, 0.143 | 0.090 | 0.149 | 38 | 7396 |
| **Study sample size (N)** | -0.006 | -0.007, 0.006 | 0.006 | 0.358 | 38 | 7396 |
| **SGLT2 inhibitor dose** | -0.882 | -1.246, -0.519 | 0.185 | **<0.0001** | 38 | 7396 |
| **SGLT2 inhibitor drug:** |  | | | | | |
| **DAPA vs. others** | 0.154 | -0.706,1.014 | 0.439 | 0.726 | 38 | 7396 |
| **EMPA vs. others** | 0.122 | -0.650, 0.894 | 0.395 | 0.756 | 38 | 7396 |
| **CANA vs. others** | -0.916 | -2.392, 0.559 | 0.753 | 0.224 | 38 | 7396 |
| **IPRA vs others** | 0.817 | -0.363, 1.996 | 0.602 | 0.175 | 38 | 7396 |
| **SOTA vs. others** | -0.351 | -1.129, 0.427 | 0.397 | 0.477 | 38 | 7396 |
| **Pre-randomization insulin optimization**  **(present vs. absent)** | -0.587 | -0.115, 1.289 | 0.358 | 0.238 | 38 | 7396 |
| **Risk-of-Bias**  **(high/unclear vs. low)** | 0.101 | -0.382, 0.706 | 0.174 | 0.648 | 38 | 7396 |
| **Univariable Meta-Regression: treatment-related predictors of BMI changes (%)** | | | | | | |
| **Moderator** | **Coefficient** | **95%CI** | **SE** | **P** | **N-comparisons** | **N-participants** |
| **Total ID change (%)** | 0.017 | -0.087, 0.122 | 0.053 | 0.745 | 38 | 7396 |
| **Basal ID change (%)** | 0.034 | -0.024, 0.092 | 0.030 | 0.247 | 38 | 7396 |
| **Bolus ID change (%)** | -0.013 | -0.051, 0.026 | 0.020 | 0.518 | 38 | 7396 |
| **TID change (%)/baseline BMI ratio** | -192.985 | -430.709, 44.738 | 121.790 | 0.112 | 38 | 7396 |
| **TID change (%)/baseline RIS ratio (IU^2^/kg/d)** | 0.050 | -0.024, 0.124 | 0.038 | 0.185 | 38 | 7396 |
| **Residual INS-SGLT2i effect(%)** | -0.009 | -0.059, 0.040 | 0.025 | 0.708 | 38 | 7396 |
| **FPG change(mg/dL)** | -0.002 | -0.029,0.025 | 0.014 | 0.897 | 38 | 7396 |
| **HBA1c(%) change** | 0.715 | -2.327, 3.757 | 1.552 | 0.645 | 29 | 7243 |
| **eGDR change (%)** | -0.364 | -0.483 -0.244 | 0.062 | **0.0001** | 38 | 7396 |
| **RIS change (%)** | 0.008 | -0.026, 0.042 | 0.017 | 0.639 | 38 | 7396 |
| **BHB change (mmol/L)** | 5.641 | -0.006, 11.288 | 2.881 | 0.061 | 24 | 2193 |
| **DKA** | -0.176 | -0.290, -0.062 | 0.058 | **0.020** | 38 | 7396 |
| **Volume depletion events** | -0.221 | -0.448, 0.006 | 0.116 | 0.066 | 38 | 7396 |
| **UTIs** | 0.067 | -0.073, 0.038 | 0.030 | 0.592 | 38 | 7396 |
| **GTIs** | 0.068 | -0.320, 0.457 | 0.198 | 0.730 | 38 | 7396 |
| **Respiratory infections** | -0.020 | -0.076, 0.041 | 0.131 | 0.813 | 38 | 7396 |

**Abbreviations.** DKA: diabetic ketoacidosis FPG: fasting plasma glucose; ID: insulin dose, CIS: continuous subcutaneous infusion; eGDR: estimated Glucose Disposal rate; eGFR: estimated glomerular filtration rate; BHB: Beta-Hydroxybutyrate;; RIS: relative insulin sensitivity; SGLT2i: SGLT2 inhibitor GTI: genital tract infections; UTI: urinary tract infections

**S1 Table F. Univariable meta-regression for moderators of changes in systolic blood pressure**

| **Univariable Meta-Regression: baseline predictors of change in sysBP (mmHg)** | | | | | | |
| --- | --- | --- | --- | --- | --- | --- |
| **Patient-related factors** |  | | | | | |
| **Moderator** | **Coefficient** | **95%CI** | **SE** | **P** | **N-comparisons** | **N-participants** |
| **Age(yr)** | 0.002 | -0.155, 0.158 | 0.080 | 0.985 | 38 | 7396 |
| **Gender(% M)** | 0.038 | -0.095 0.130 | 0.058 | 0.761 | 38 | 7396 |
| **Ethnicity:** |  | | | | | |
| **Caucasians (%)** | -0.007 | -0.035, 0.022 | 0.001 | 0.014 | 38 | 7396 |
| **Asians (%)** | 0.002 | -0.006 0.007 | 0.006 | 0.723 | 38 | 7396 |
| **Hispanic (%)** | 0.005 | -0.018, 0.227 | 0.011 | 0.676 | 38 | 7396 |
| **Black (%)** | -0.049 | -0.290 0.191 | 0.123 | **0**.688 | 38 | 7396 |
| **CSI users (%)** | -2.784 | -9.295 3.727 | 0.322 | 0.402 | 38 | 7396 |
| **Total ID (IU/d)** | -0.000 | -0.130, 0.129 | 0.066 | 0.996 | 38 | 7396 |
| **Diabetes duration(yr)** | 0.002 | -0.201, 0.206 | 0.104 | 0.986 | 38 | 7396 |
| **BMI (kg/m^2^)** | 0.065 | -0.352 0.481 | 0.213 | 0.761 | 38 | 7396 |
| **Sys BP(mmHg)** | 0.190 | 0.004, 0.375 | 0.085 | **0.045** | 38 | 7396 |
| **HbA1c(%)** | -0.074 | -1.645 1.497 | 0.801 | 0.927 | 38 | 7396 |
| **FPG(mg/dL)** | -0.003 | -0.056 0.050 | 0.027 | 0.913 | 38 | 7396 |
| **eGDR(mg/kg/min)** | -0.187 | -1.217, 0.843 | 0.526 | 0.722 | 38 | 7396 |
| **Renal function stage** | 0.163 | -0.858, 1.184 | 0.521 | 0.755 | 38 | 7396 |
| **eGFR( ml/min/1.73 m^2^)** | 0.076 | -0.097 0.249 | 0.088 | 0.391 | 38 | 7396 |
| **Fasting BHB(mmol/L)** | -0.443 | -13.759 12.874 | 6.794 | 0.948 | 24 | 2538 |
| **Study design-related factors** |  | | | | | |
| **Study duration (wk)** | 0.011 | -0.033 0.055 | 0.022 | 0.622 | 38 | 7396 |
| **Study sample size (N)** | 0.003 | -0.011 0.016 | 0.007 | 0.706 | 38 | 7396 |
| **SGLT2 inhibitor dose** | -1.520 | -2.639, -0.400 | 0.570 | **0.003** | 38 | 7396 |
| **SGLT2 inhibitor drug:** |  | | | | | |
| **DAPA vs. others** | 0.197 | -2.533 2.927 | 1.393 | 0.888 | 38 | 7396 |
| **EMPA vs. others** | 0.114 | -1.538 1.767 | 0.843 | 0.892 | 38 | 7396 |
| **CANA vs. others** | 0.107 | -0.096, 0.300 | 0.104 | 0.607 | 38 | 7396 |
| **IPRA vs others** | 0.006 | -0.239, 0.241 | 0.125 | 0.814 | 38 | 7396 |
| **SOTA vs. others** | -0.077 | -1.460 1.305 | 0.705 | 0.913 | 38 | 7396 |
| **Pre-randomization insulin optimization**  **(present vs. absent)** | -0.318 | -1.690 ,1.055 | 0.700 | 0.650 | 38 | 7396 |
| **Risk-of-Bias**  **(high-unclear vs. low)** | 0.130 | -0.417, 0.681 | 0.149 | 0.912 | 38 | 7396 |
| **Univariable Meta-Regression: treatment-related predictors of changes in sysBP (mmHg)** | | | | | | |
| **Moderator** | **Coefficient** | **95%CI** | **SE** | **P** | **N-comparisons** | **N-participants** |
| **Total ID change (%)** | 0.092 | -0.148, 0.333 | 0.123 | 0.459 | 38 | 7396 |
| **Basal ID change (%)** | 0.093 | -0.070 0.257 | 0.084 | 0.264 | 38 | 7396 |
| **Bolus ID change (%)** | 0.090 | -0.047, 0.077 | 0.034 | 0.603 | 38 | 7396 |
| **TID change (%)/baseline BMI ratio** | 101.612 | -492.150, 695.373 | 302.943 | 0.737 | 38 | 7396 |
| **TID change (%)/baseline RIS ratio (IU^2^/kg/d)** | 3.667 | -13.339, 20.673 | 8.677 | 0.673 | 38 | 7396 |
| **Residual INS-SGLT2i effect(%)** | 0.008 | -0.135, 0.151 | 0.073 | 0.916 | 38 | 7396 |
| **BMI change (%)** | 0.429 | -0.348 1.206 | 0.027 | 0.396 | 38 | 7396 |
| **HbA1c (%) change** | -0.307 | -7.938, 7.325 | 3.894 | 0.937 | 29 | 7243 |
| **FPG change(mg/dL)** | 0.010 | -0.045, 0.065 | 0.028 | 0.721 | 38 | 7396 |
| **Time-in-ragne (%) change** | 0.087 | -0.087 0.261 | 0.089 | 0.327 | 31 | 3050 |
| **MAGE (mg/dL) change** | 0.011 | -0.041 0.063 | 0.027 | 0.682 | 31 | 3050 |
| **eGDR change (%)** | 0.087 | -0.333, 0.507 | 0.214 | 0.685 | 38 | 7396 |
| **RIS change (%)** | 0.052 | -0.048, 0.152 | 0.051 | 0.305 | 38 | 7396 |
| **BHB change (mmol/L)** | -1.584 | -9.640, 6.472 | 4.110 | 0.700 | 24 | 2538 |
| **DKA** | -0.046 | -0.293 0.201 | 0.126 | 0.717 | 38 | 7396 |
| **Volume depletion events** | 0.014 | -0.268 0.297 | 0.144 | 0.921 | 38 | 7396 |
| **UTIs** | -0.024 | -0.076, 0.041 | 0.033 | 0.540 | 38 | 7396 |
| **GTIs** | 0.170 | -0.346 0.687 | 0.264 | 0.518 | 38 | 7396 |
| **Respiratory infections** | -0.019 | -0.079, 0.043 | 0.134 | 0.782 | 38 | 7396 |
| **Severe hypoglycemia** | -0.000 | -0.003 0.003 | 0.002 | 0.961 | 38 | 7396 |

**Abbreviations.** DKA: diabetic ketoacidosis FPG: fasting plasma glucose; ID: insulin dose, CIS: continuous subcutaneous infusion; eGDR: estimated Glucose Disposal rate; eGFR: estimated glomerular filtration rate; BHB: Beta-Hydroxybutyrate;; RIS: relative insulin sensitivity; SGLT2i: SGLT2 inhibitor GTI: genital tract infections; UTI: urinary tract infections

**S1 Table G. Univariable meta-regression for moderators of eGFR changes.**

| **Univariable Meta-Regression: baseline predictors of change in eGFR (ml/min/1.73 m^2^)** | | | | | | |
| --- | --- | --- | --- | --- | --- | --- |
| **Patient-related factors** |  | | | | | |
| **Moderator** | **Coefficient** | **95%CI** | **SE** | **P** | **N-comparisons** | **N-participants** |
| **Age(yr)** | 0.002 | -0.155, 0.158 | 0.080 | 0.985 | 38 | 7396 |
| **Gender(% M)** | 0.001 | -0.005, 0.008 | 0.003 | 0.654 | 38 | 7396 |
| **Ethnicity:** |  | | | | | |
| **Caucasians (%)** | 0.001 | -0.002 0.003 | 0.001 | 0.607 | 38 | 7396 |
| **Asians (%)** | 0.001 | -0.001 0.002 | 0.001 | 0.538 | 38 | 7396 |
| **Hispanic (%)** | 0.004 | -0.007, 0.011 | 0.003 | 0.689 | 38 | 7396 |
| **Black (%)** | -0.013 | -0.032 0.005 | 0.009 | **0**.157 | 38 | 7396 |
| **CSI users (%)** | 0.190 | -0.179, 0.559 | 0.188 | 0.313 | 38 | 7396 |
| **Total ID (IU/d)** | -0.011 | -0.121 0.098 | 0.056 | 0.838 | 38 | 7396 |
| **Diabetes duration(yr)** | 0.002 | -0.201, 0.206 | 0.104 | 0.986 | 38 | 7396 |
| **BMI (kg/m^2^)** | 0.065 | -0.352 0.481 | 0.213 | 0.761 | 38 | 7396 |
| **Systolic BP(mmHg)** | 0.003 | -0.024 0.029 | 0.013 | 0.851 | 38 | 7396 |
| **HbA1c(%)** | 0.079 | -1.299, 1.457 | 0.703 | 0.911 | 29 | 7243 |
| **FPG(mg/dL)** | -0.001 | -0.005 0.002 | 0.002 | 0.529 | 38 | 7396 |
| **eGDR(mg/kg/min)** | 0.001 | -0.068, 0.069 | 0.035 | 0.981 | 38 | 7396 |
| **Renal function stage** | -0.577 | -1.153, -0.002 | 0.287 | **0.044** | 38 | 7396 |
| **Baseline eGFR( ml/min/1.73 m^2^)** | 0.026 | -0.098, 0.150 | 0.063 | 0.684 | 38 | 7396 |
| **Fasting BHB(mmol/L)** | 0.001 | -0.001, 0.001 | 0.001 | 0.915 | 24 | 2538 |
| **Study design-related factors** |  | | | | | |
| **Study duration (wk)** | 0.027 | 0.003, 0.049 | 0.010 | **0.013** | 38 | 7396 |
| **Study sample size (N)** | -0.001 | -0.002, 0.001 | 0.001 | 0.154 | 38 | 7396 |
| **SGLT2 inhibitor dose** | 0.229 | -0.607 1.064 | 0.426 | 0.591 | 38 | 7396 |
| **SGLT2 inhibitor drug:** |  | | | | | |
| **DAPA vs. others** | -0.002 | -0.117, 0.113 | 0.059 | 0.972 | 38 | 7396 |
| **EMPA vs. others** | -0.055 | -0.144, 0.034 | 0.045 | 0.225 | 38 | 7396 |
| **CANA vs. others** | 0.102 | -0.091, 0.295 | 0.099 | 0.301 | 38 | 7396 |
| **IPRA vs others** | 0.001 | -0.234, 0.236 | 0.120 | 0.993 | 38 | 7396 |
| **SOTA vs. others** | 0.033 | -0.057, 0.123 | 0.046 | 0.477 | 38 | 7396 |
| **Pre-randomization insulin optimization**  **(present vs. absent)** | 0.094 | 0.002, 0.186 | 0.047 | **0.044** | 38 | 7396 |
| **Risk-of-Bias**  **(high/unclear vs. low)** | 0.131 | -0.412, 0.676 | 0.144 | 0.813 | 38 | 7396 |
| **Univariable Meta-Regression: treatment-related predictors of change in eGFR (ml/min/1.73 m2)** | | | | | | |
| **Moderator** | **Coefficient** | **95%CI** | **SE** | **P** | **N-comparisons** | **N-participants** |
| **Total ID change (%)** | -0.044 | -0.231 0.144 | 0.096 | 0.646 | 38 | 7396 |
| **Basal ID change (%)** | 0.005 | -0.148 0.158 | 0.078 | 0.953 | 38 | 7396 |
| **Bolus ID change (%)** | 0.001 | -0.099 0.101 | 0.051 | 0.981 | 38 | 7396 |
| **TID change (%)/baseline BMI ratio** | 19.914 | -34.857, 74.685 | 27.945 | 0.476 | 38 | 7396 |
| **TID change (%)/baseline RIS ratio (IU^2^/kg/d)** | 0.002 | -0.064, 0.069 | 0.034 | 0.949 | 38 | 7396 |
| **Residual INS-SGLT2i effect(%)** | -0.028 | -0.120, 0.065 | 0.047 | 0.558 | 38 | 7396 |
| **BMI change (%)** | -0.173 | -0.811 0.464 | 0.325 | 0.594 | 38 | 7396 |
| **Systolic BP change(mmHg)** | -0.066 | -0.342 0.210 | 0.141 | 0.640 | 38 | 7396 |
| **HbA1c (%) change** | -3.154 | -9.197 2.889 | 3.083 | 0.306 | 38 | 7396 |
| **FPG change(mg/dL)** | 0.001 | -0.003,0.005 | 0.002 | 0.566 | 38 | 7396 |
| **Time-in-range(%)** | 0.035 | -0.139, 0.209 | 0.089 | 0.693 | 31 | 3050 |
| **MAGE(mg/dL)** | 0.013 | -0.059, 0.085 | 0.037 | 0.729 | 31 | 3050 |
| **eGDR change (%)** | -0.024 | -0.045 -0.002 | 0.010 | **0.031** | 38 | 7396 |
| **RIS change (%)** | -0.003 | -0.009, 0.003 | 0.003 | 0.375 | 38 | 7396 |
| **BHB change (mmol/L)** | -1.584 | -9.640, 6.472 | 4.110 | 0.700 | 24 | 2538 |
| **DKA** | 0.003 | -0.012, 0.019 | 0.008 | 0.671 | 29 | 7243 |
| **Volume depletion events** | -0.017 | -0.038, 0.003 | 0.011 | 0.100 | 29 | 7243 |
| **UTIs** | -0.019 | -0.071 0.036 | 0.028 | 0.535 | 29 | 7243 |
| **GTIs** | -0.016 | -0.072, 0.037 | 0.027 | 0.579 | 29 | 7243 |
| **Respiratory infections** | -0.018 | -0.074, 0.039 | 0.129 | 0.619 | 29 | 7243 |

**Abbreviations.** DKA: diabetic ketoacidosis FPG: fasting plasma glucose; ID: insulin dose, CIS: continuous subcutaneous infusion; eGDR: estimated Glucose Disposal rate; eGFR: estimated glomerular filtration rate; BHB: Beta-Hydroxybutyrate;; RIS: relative insulin sensitivity; SGLT2i: SGLT2 inhibitor GTI: genital tract infections; UTI: urinary tract infections

**S1 Table H. Univariable meta-regression for moderators of ACR changes (mg/g)**

| **Univariable Meta-Regression: baseline predictors of ACR changes (mg/g)** | | | | | | |
| --- | --- | --- | --- | --- | --- | --- |
| **Patient-related factors** |  | | | | | |
| **Moderator** | **Coefficient** | **95%CI** | **SE** | **P** | **N-comparisons** | **N-participants** |
| **Age(yr)** | 1.625 | -2.293, 5.543 | 1.999 | 0.416 | 8 | 3052 |
| **Gender(% M)** | 0.001 | -0.005, 0.008 | 0.003 | 0.654 | 8 | 3052 |
| **Ethnicity:** |  | | | | | |
| **Caucasians (%)** | 0.001 | -0.002 0.003 | 0.001 | 0.607 | 8 | 3052 |
| **Asians (%)** | 0.001 | -0.001 0.002 | 0.001 | 0.538 | 8 | 3052 |
| **Hispanic (%)** | 0.004 | -0.007, 0.011 | 0.003 | 0.689 | 8 | 3052 |
| **Black (%)** | -0.013 | -0.032 0.005 | 0.009 | **0**.157 | 8 | 3052 |
| **CSI users (%)** | 0.190 | -0.179, 0.559 | 0.188 | 0.313 | 8 | 3052 |
| **Total ID (IU/d)** | -0.276 | -1.356, 0.804 | 0.551 | 0.616 | 8 | 3052 |
| **Diabetes duration(yr)** | -0.002 | -0.017, 0.013 | 0.008 | 0.764 | 8 | 3052 |
| **BMI (kg/m^2^)** | -1.442 | -5.545 2.662 | 2.094 | 0.491 | 8 | 3052 |
| **HbA1c(%)** | 5.186 | -21.864 ,32.236 | 13.801 | 0.707 | 8 | 3052 |
| **FPG(mg/dL)** | -0.001 | -0.005 0.002 | 0.002 | 0.529 | 8 | 3052 |
| **eGDR(mg/kg/min)** | 0.001 | -0.068, 0.069 | 0.035 | 0.981 | 8 | 3052 |
| **Renal function stage** | -0.046 | -0.098, 0.006 | 0.026 | 0.081 | 8 | 3052 |
| **Fasting BHB(mmol/L)** | 0.001 | -0.001, 0.001 | 0.001 | 0.915 | 8 | 3052 |
| **Study design-related factors** |  | | | | | |
| **Study duration (wk)** | 0.001 | -0.001, 0.004 | 0.001 | 0.251 | 8 | 3052 |
| **Study sample size (N)** | -0.001 | -0.002, 0.001 | 0.001 | 0.154 | 8 | 3052 |
| **SGLT2 inhibitor dose** | -9.977 | -16.076, -3.878 | 3.812 | **0.004** | 8 | 3052 |
| **SGLT2 inhibitor drug:** |  | | | | | |
| **DAPA vs. others** | -0.002 | -0.117, 0.113 | 0.059 | 0.972 | 8 | 3052 |
| **EMPA vs. others** | -0.055 | -0.144, 0.034 | 0.045 | 0.225 | 8 | 3052 |
| **CANA vs. others** | 0.102 | -0.091, 0.295 | 0.099 | 0.301 | 8 | 3052 |
| **IPRA vs others** | 0.001 | -0.234, 0.236 | 0.120 | 0.993 | 8 | 3052 |
| **SOTA vs. others** | 0.033 | -0.057, 0.123 | 0.046 | 0.477 | 8 | 3052 |
| **Pre-randomization insulin optimization**  **(present vs. absent)** | 0.094 | 0.002, 0.186 | 0.047 | **0.044** | 8 | 3052 |
| **Risk-of-Bias**  **(high/unclear vs. low)** | 0.131 | -0.412, 0.676 | 0.144 | 0.813 | 8 | 3052 |
| **Univariable Meta-Regression: treatment-related predictors of ACR changes (mg/g)** | | | | | | |
| **Moderator** | **Coefficient** | **95%CI** | **SE** | **P** | **N-comparisons** | **N-participants** |
| **Total ID change (%)** | 45.771 | -94.014, 185.557 | 71.320 | 0.521 | 8 | 3052 |
| **Basal ID change (%)** | 0.006 | -0.004, 0.016 | 0.005 | 0.261 | 8 | 3052 |
| **Bolus ID change (%)** | 0.014 | -0.042, 0.072 | 0.029 | 0.594 | 8 | 3052 |
| **TID change (%)/baseline BMI ratio** | 19.914 | -34.857, 74.685 | 27.945 | 0.476 | 8 | 3052 |
| **TID change (%)/baseline RIS ratio (IU^2^/kg/d)** | -0.002 | -0.007, 0.003 | 0.003 | 0.446 | 8 | 3052 |
| **Residual INS-SGLT2i effect(%)** | 0.345 | -0.729 1.420 | 0.548 | 0.529 | 8 | 3052 |
| **BMI change (%)** | -0.100 | -0.225, 0.025 | 0.064 | 0.116 | 8 | 3052 |
| **HbA1c(%) change** | 19.922 | -59.945, 99.788 | 40.749 | 0.626 | 8 | 3052 |
| **FPG change(mg/dL)** | 0.001 | -0.003,0.005 | 0.002 | 0.566 | 8 | 3052 |
| **SysBP(mmHg)** | 0.880 | -2.016, 3.775 | 1.477 | 0.552 | 8 | 3052 |
| **eGDR change (%)** | -2.340 | -7.235, 2.555 | 2.498 | 0.349 | 8 | 3052 |
| **RIS change (%)** | 0.005 | -0.013, 0.023 | 0.009 | 0.590 | 8 | 3052 |
| **BHB change (mmol/L)** | -0.434 | -1.064, 0.196 | 0.321 | 0.177 | 8 | 3052 |
| **Time-in-range (%) change** | -0.919 | -1.795,-0.042 | 0.349 | **0.041** | 8 | 3052 |
| **MAGE (mg/dL) change** | 0.913 | 0.078, 1.729 | 0.412 | **0.010** | 8 | 3052 |
| **DKA** | 0.003 | -0.012, 0.019 | 0.008 | 0.671 | 8 | 3052 |
| **Volume depletion events** | -0.017 | -0.038, 0.003 | 0.011 | 0.100 | 8 | 3052 |
| **UTIs** | -0.019 | -0.071 0.036 | 0.028 | 0.535 | 8 | 3052 |
| **GTIs** | -0.016 | -0.072, 0.037 | 0.027 | 0.579 | 8 | 3052 |
| **Respiratory infections** | -0.018 | -0.074, 0.039 | 0.129 | 0.619 | 8 | 3052 |

**Abbreviations.** DKA: diabetic ketoacidosis FPG: fasting plasma glucose; ID: insulin dose, CIS: continuous subcutaneous infusion; eGDR: estimated Glucose Disposal rate; eGFR: estimated glomerular filtration rate; BHB: Beta-Hydroxybutyrate;; RIS: relative insulin sensitivity; SGLT2i: SGLT2 inhibitor GTI: genital tract infections; UTI: urinary tract infections

**S1 Table I. Univariable meta-regression for moderators of RR of diabetic eye disorders**

| **Univariable Meta-Regression: baseline predictors of eye disorders** | | | | | | |
| --- | --- | --- | --- | --- | --- | --- |
| **Patient-related factors** |  | | | | | |
| **Moderator** | **Coefficient** | **95%CI** | **SE** | **P** | **N-comparisons** | **N-participants** |
| **Age(yr)** | 1.625 | -2.293, 5.543 | 1.999 | 0.416 | 38 | 7396 |
| **Gender(% M)** | 0.001 | -0.005, 0.008 | 0.003 | 0.654 | 38 | 7396 |
| **Ethnicity:** |  | | | | | |
| **Caucasians (%)** | 0.001 | -0.002 0.003 | 0.001 | 0.607 | 38 | 7396 |
| **Asians (%)** | 0.001 | -0.001 0.002 | 0.001 | 0.538 | 38 | 7396 |
| **Hispanic (%)** | 0.004 | -0.007, 0.011 | 0.003 | 0.689 | 38 | 7396 |
| **Black (%)** | -0.013 | -0.032 0.005 | 0.009 | **0**.157 | 38 | 7396 |
| **CSI users (%)** | 0.190 | -0.179, 0.559 | 0.188 | 0.313 | 38 | 7396 |
| **Total ID (IU/d)** | -0.276 | -1.356, 0.804 | 0.551 | 0.616 | 38 | 7396 |
| **Diabetes duration(yr)** | -0.002 | -0.017, 0.013 | 0.008 | 0.764 | 38 | 7396 |
| **BMI (kg/m^2^)** | -1.442 | -5.545 2.662 | 2.094 | 0.491 | 38 | 7396 |
| **HbA1c(%)** | 5.186 | -21.864 ,32.236 | 13.801 | 0.707 | 38 | 7396 |
| **FPG(mg/dL)** | -0.001 | -0.005 0.002 | 0.002 | 0.529 | 38 | 7396 |
| **eGDR(mg/kg/min)** | 0.001 | -0.068, 0.069 | 0.035 | 0.981 | 38 | 7396 |
| **Renal function stage** | -0.046 | -0.098, 0.006 | 0.026 | 0.081 | 38 | 7396 |
| **Fasting BHB(mmol/L)** | 0.001 | -0.001, 0.001 | 0.001 | 0.915 | 24 | 2538 |
| **Study design-related factors** |  | | | | | |
| **Study duration (wk)** | 0.001 | -0.001, 0.004 | 0.001 | 0.251 | 38 | 7396 |
| **Study sample size (N)** | -0.001 | -0.002, 0.001 | 0.001 | 0.154 | 38 | 7396 |
| **SGLT2 inhibitor dose** | -0.742 | -1.443,-0.041 | 0.317 | **0.031** | 38 | 7396 |
| **SGLT2 inhibitor drug:** |  | | | | | |
| **DAPA vs. others** | -0.002 | -0.117, 0.113 | 0.059 | 0.972 | 38 | 7396 |
| **EMPA vs. others** | -0.055 | -0.144, 0.034 | 0.045 | 0.225 | 38 | 7396 |
| **CANA vs. others** | 0.102 | -0.091, 0.295 | 0.099 | 0.301 | 38 | 7396 |
| **IPRA vs others** | 0.001 | -0.234, 0.236 | 0.120 | 0.993 | 38 | 7396 |
| **SOTA vs. others** | 0.033 | -0.057, 0.123 | 0.046 | 0.477 | 38 | 7396 |
| **Pre-randomization insulin optimization**  **(present vs. absent)** | 0.090 | -0.002, 0.182 | 0.047 | 0.101 | 38 | 7396 |
| **Risk-of-Bias**  **(high/unclear vs. low)** | 0.131 | -0.412, 0.676 | 0.144 | 0.813 | 38 | 7396 |
| **Univariable Meta-Regression: treatment-related predictors of eye disorders** | | | | | | |
| **Moderator** | **Coefficient** | **95%CI** | **SE** | **P** | **N-comparisons** | **N-participants** |
| **Total ID change (%)** | 45.771 | -94.014, 185.557 | 71.320 | 0.521 | 38 | 7396 |
| **Basal ID change (%)** | 0.006 | -0.004, 0.016 | 0.005 | 0.261 | 38 | 7396 |
| **Bolus ID change (%)** | 0.014 | -0.042, 0.072 | 0.029 | 0.594 | 38 | 7396 |
| **TID change (%)/baseline BMI ratio** | 19.914 | -34.857, 74.685 | 27.945 | 0.476 | 38 | 7396 |
| **TID change (%)/baseline RIS ratio (IU^2^/kg/d)** | -0.002 | -0.007, 0.003 | 0.003 | 0.446 | 38 | 7396 |
| **Residual INS-SGLT2i effect(%)** | 0.345 | -0.729 1.420 | 0.548 | 0.529 | 38 | 7396 |
| **BMI change (%)** | -0.100 | -0.225, 0.025 | 0.064 | 0.116 | 38 | 7396 |
| **HbA1c(%) change** | 1.583 | -4.563 7.729 | 3.136 | 0.632 | 29 | 7243 |
| **FPG change(mg/dL)** | 0.001 | -0.003,0.005 | 0.002 | 0.566 | 38 | 7396 |
| **SysBP(mmHg)** | 0.880 | -2.016, 3.775 | 1.477 | 0.552 | 38 | 7396 |
| **eGDR change (%)** | -0.075 | -0.271 0.121 | 0.100 | 0.451 | 38 | 7396 |
| **RIS change (%)** | 0.005 | -0.013, 0.023 | 0.009 | 0.590 | 38 | 7396 |
| **BHB change (mmol/L)** | -0.434 | -1.064, 0.196 | 0.321 | 0.177 | 24 | 2538 |
| **Time-in-range (%) change** | -0.117 | -0.211, -0.004 | 0.049 | **0.009** | 31 | 3050 |
| **MAGE (mg/dL) change** | -0.002 | -0.049, 0.045 | 0.024 | 0.929 | 31 | 3050 |
| **DKA** | 0.003 | -0.012, 0.019 | 0.008 | 0.671 | 38 | 7396 |
| **Volume depletion events** | -0.017 | -0.038, 0.003 | 0.011 | 0.100 | 38 | 7396 |
| **UTIs** | -0.019 | -0.071 0.036 | 0.028 | 0.535 | 38 | 7396 |
| **GTIs** | -0.016 | -0.072, 0.037 | 0.027 | 0.579 | 38 | 7396 |
| **Respiratory infections** | -0.018 | -0.074, 0.039 | 0.129 | 0.619 | 38 | 7396 |

**Abbreviations.** DKA: diabetic ketoacidosis FPG: fasting plasma glucose; ID: insulin dose, CIS: continuous subcutaneous infusion; eGDR: estimated Glucose Disposal rate; eGFR: estimated glomerular filtration rate; BHB: Beta-Hydroxybutyrate;; RIS: relative insulin sensitivity; SGLT2i: SGLT2 inhibitor GTI: genital tract infections; UTI: urinary tract infections

**S1 Table J: Summary of main findings of meta-analysis for safety outcomes in included RCTs**

| **Outcome** | **Comparisons**  **(n)** | **Events/Participants**  **(n/N)** | | **RR**  [**95%CI**] | **I^2^**  **(%)** |
| --- | --- | --- | --- | --- | --- |
|  |  | **SGLT2 inhibitors** | **Control** |  |  |
| **Diabetic ketoacidosis (DKA)** | **38** | **175/4808** | **21/2588** | \| **RR: 2.81**  **(1.97, 4.01)** \| \| --- \| | **0** |
| **Occurring at blood glucose>250 mg/dL**  **N (% total events)** |  | 121 (69%) | 13(67%) |  |  |
| **Occurring at blood glucose≤250 mg/dL(“euglycemic” DKA)**  **n(% total events)** |  | 54(31%) | 8(33%) |  |  |
| **Hypoglicemia** | **38** | **3978/4808** | **2211/2588** | \| **RR: 1.00**  **(0.99, 1.01)** \| \| --- \| | **0** |
| **Severe hypoglycemia** | **38** | **118/4808** | **77/2588** | \| **RR: 0.81**  **(0.61, 1.07)** \| \| --- \| | **0** |
| **Genital mycotic infections (GTIs)** | **38** | **463/4808** | **68/2588** | \| **RR: 3.18**  **(2.49, 4.06)** \| \| --- \| | **0** |
| **Urinary tract infections (UTIs)** | **38** | **287/4808** | **144/2588** | \| **RR: 0.99**  **(0.81, 1.21)** \| \| --- \| | **0** |
| **Sinusitis** | **38** | **156/4808** | **96/2588** | \| **RR: 0.91**  **[0.71, 1.17)** \| \| --- \| | **0** |
| **Nasopharingytis** | **38** | **172/4808** | **84/2588** | \| **RR: 0.89**  **(0.69, 1.13)** \| \| --- \| | **0** |
| **Major adverse cardiovascular outcomes (MACE)** | **38** | **28/4808** | **11/2588** | \| **RR: 1.06**  **(0.54, 2.08)** \| \| --- \| | **0** |
| **AMI** |  | 15 | 4 |  |  |
| **Stroke** |  | 4 | 2 |  |  |
| **Hospitalization for HF/UA** |  | 2 | 3 |  |  |
| **Coronary revascularization** |  | 7 | 2 |  |  |
| **Eye disorders** | **38** | **4/4808** | **10/2588** | \| **RR: 0.27**  **(0.11, 0.67)** \| \| --- \| | **0** |
| **Haemorrhagic retinopathy**  **n(% total events)** |  | 3 | 8 |  |  |
| **Macular degeneration**  **n(% total events)** |  | 0 | 0 |  |  |
| **Glaucoma**  **n(% total events)** |  | 1 | 1 |  |  |
| **Vision loss**  **n(% total events)** |  | 0 | 1 |  |  |
| **Diarrhea** | **38** | **178/4808** | **75/2588** | \| **RR: 1.25**  **(0.96, 1.63)** \| \| --- \| | **0** |
| **Nausea-vomiting** | **38** | **117/4808** | **48/2588** | **RR: 1.10**  **(0.80, 1.51)**   \|  \| \| --- \| | **0** |
| **Renal events** | **38** | **68/4808** | **24/2588** | **RR: 1.28**  **(0.81, 2.01)** | **0** |
| **Volume depletion events** | **6** | **99/4808** | **21/2588** | **RR: 1.53**  **(1.03, 2.28)** | **0** |
| **Bone fractures** | **38** | **60/4808** | **35/2588** | **RR: 0.79**  **(0.52, 1.20)** | **0** |
| **Limb amputation** | **38** | **4/4808** | **3/2588** | **RR: 0.56**  **(0.16, 1.98)** | **0** |
| **Suspected drug-induced liver injury** | **38** | **4/4808** | **3/2588** | **RR: 0.56**  **(0.17, 1.87)**   \|  \| \| --- \| | **0** |
| **Venous thromboembolism** | 38 | **5/4808** | **2/2588** | **RR: 0.74**  **(0.21, 2.63)**  - | 0 |
| **Cancer** | **38** | **14/4808** | **7/2588** | **RR: 0.72**  **(0.31, 1.70)**   \|  \| \| --- \| | **0** |
| **Serious AEs** | **38** | **393/4808** | **177/2588** | **RR: 1.12**  **(0.94, 1.34)** | 0 |
| **All-cause death** | **38** | **2/4808** | **4/2588** | **RR: 0.38**  **(0.10, 1.40)** | **0** |

**Abbreviations:** AE : adverse events; VTE:Venousthromboembolism;Sota: sotagliflozin;TID: total daily insulin dose; plcb: placebo; HF: heart failure; UA: unstable angina.

*****the percentage refers to the percentage of all patients experiencing that AE

For all outcomes, the length of follow-up ranged 4 to 52 weeks

**S1 Table L. Univariable meta-regression for moderators of RR for GTI(s)**

| **Univariable Meta-Regression: baseline predictors of GTIs** | | | | | | |
| --- | --- | --- | --- | --- | --- | --- |
| **Patient-related factors** |  | | | | | |
| **Moderator** | **Coefficient** | **95%CI** | **SE** | **P** | **N-comparisons** | **N-participants** |
| **Age(yr)** | 0.018 | -0.064 0.101 | 0.042 | 0.661 | 38 | 7396 |
| **Gender(% M)** | 0.005 | -0.030, 0.040 | 0.018 | 0.789 | 38 | 7396 |
| **Ethnicity:** |  | | | | | |
| **Caucasians (%)** | 0.013 | -0.002 0.027 | 0.007 | 0.081 | 38 | 7396 |
| **Asians (%)** | 0.008 | -0.003, 0.020 | 0.006 | 0.140 | 38 | 7396 |
| **Hispanic (%)** | -0.095 | -0.246, 0.056 | 0.077 | 0.214 | 38 | 7396 |
| **Black (%)** | -0.011 | -0.034 0.007 | 0.011 | **0**.394 | 38 | 7396 |
| **CSI users (%)** | 0.550 | -2.678, 3.777 | 1.647 | 0.739 | 38 | 7396 |
| **Total ID (IU/d)** | 0.066 | 0.017 0.115 | 0.025 | **0.009** | 38 | 7396 |
| **Diabetes duration(yr)** | 0.018 | -0.084 0.119 | 0.052 | 0.733 | 38 | 7396 |
| **BMI (kg/m^2^)** | 0.219 | 0.055 0.383 | 0.084 | **0.009** | 38 | 7396 |
| **HbA1c(%)** | 0.073 | -0.666 0.811 | 0.377 | 0.847 | 38 | 7396 |
| **FPG(mg/dL)** | 0.010 | -0.009 0.029 | 0.010 | 0.290 | 38 | 7396 |
| **Time-in-rageg(%)** | -0.049 | -0.118 0.019 | 0.035 | 0.159 |  |  |
| **MAGE(mg/dL)** | 0.003 | -0.005 0.012 | 0.004 | 0.428 |  |  |
| **eGDR(mg/kg/min)** | -0.455 | -0.876 -0.034 | 0.215 | **0.034** | 38 | 7396 |
| **Renal function stage** | -0.354 | -0.784, 0.076 | 0.219 | 0.107 | 38 | 7396 |
| **Fasting BHB(mmol/L)** | 0.011 | -0.011, 0.011 | 0.010 | 0.713 | 24 | 2538 |
| **Study design-related factors** |  | | | | | |
| **Study duration (wk)** | -0.033 | -0.209, 0.143 | 0.090 | 0.149 | 38 | 7396 |
| **Study sample size (N)** | -0.006 | -0.007, 0.006 | 0.006 | 0.358 | 38 | 7396 |
| **SGLT2 inhibitor dose** | 0.218 | -0.179 0.616 | 0.203 | 0.281 | 38 | 7396 |
| **SGLT2 inhibitor drug:** |  | | | | | |
| **DAPA vs. others** | 0.154 | -0.706,1.014 | 0.439 | 0.726 | 38 | 7396 |
| **EMPA vs. others** | 0.122 | -0.650, 0.894 | 0.395 | 0.756 | 38 | 7396 |
| **CANA vs. others** | -0.916 | -2.392, 0.559 | 0.753 | 0.224 | 38 | 7396 |
| **IPRA vs others** | 0.817 | -0.363, 1.996 | 0.602 | 0.175 | 38 | 7396 |
| **SOTA vs. others** | -0.351 | -1.129, 0.427 | 0.397 | 0.477 | 38 | 7396 |
| **Pre-randomization insulin optimization**  **(present vs. absent)** | -0.587 | -0.115, 1.289 | 0.358 | 0.238 | 38 | 7396 |
| **Risk-of-Bias**  **(high/unclear vs. low)** | 0.101 | -0.382, 0.706 | 0.174 | 0.648 | 38 | 7396 |
| **Univariable Meta-Regression: treatment-related predictors of GTIs** | | | | | | |
| **Moderator** | **Coefficient** | **95%CI** | **SE** | **P** | **N-comparisons** | **N-participants** |
| **Total ID change (%)** | 0.058 | -0.025 0.140 | 0.042 | 0.170 | 38 | 7396 |
| **Basal ID change (%)** | 0.034 | -0.024, 0.092 | 0.030 | 0.247 | 38 | 7396 |
| **Bolus ID change (%)** | -0.013 | -0.051, 0.026 | 0.020 | 0.518 | 38 | 7396 |
| **TID change (%)/baseline BMI ratio** | -192.985 | -430.709, 44.738 | 121.790 | 0.112 | 38 | 7396 |
| **TID change (%)/baseline RIS ratio (IU^2^/kg/d)** | 0.050 | -0.024, 0.124 | 0.038 | 0.185 | 38 | 7396 |
| **Residual INS-SGLT2i effect(%)** | 0.019 | -0.024 0.061 | 0.022 | 0.389 | 38 | 7396 |
| **HBA1c(%) change** | 0.503 | -2.247 3.252 | 1.403 | 0.720 | 29 | 7243 |
| **FPG change(mg/dL)** | 0.026 | 0.001 0.052 | 0.013 | **0.041** | 38 | 7396 |
| **Time-in-range(%) change** | 0.001 | -0.092 0.094 | 0.047 | 0.987 | 31 | 3050 |
| **MAGE(mg/dL) change** | 0.015 | -0.016 0.047 | 0.016 | 0.346 | 31 | 3050 |
| **eGDR change (%)** | 0.154 | 0.020 0.288 | 0.068 | **0.025** | 38 | 7396 |
| **RIS change (%)** | 0.008 | -0.026, 0.042 | 0.017 | 0.639 | 38 | 7396 |
| **BMI change(%)** | -0.363 | -0.636 -0.089 | 0.139 | **0.009** | 38 | 7396 |
| **SysBP change(mmHg)** | 0.018 | -0.122 0.157 | 0.071 | 0.803 | 38 | 7396 |
| **BHB change (mmol/L)** | 5.641 | -0.006, 11.288 | 2.881 | 0.061 | 24 | 2538 |
| **DKA** | -0.176 | -0.290, -0.062 | 0.058 | **0.002** | 38 | 7396 |
| **Volume depletion events** | 0.029 | -0.091 0.148 | 0.061 | 0.635 | 38 | 7396 |
| **UTIs** | 0.067 | -0.073, 0.038 | 0.030 | 0.592 | 38 | 7396 |
| **Respiratory infections** | -0.020 | -0.076, 0.041 | 0.131 | 0.813 | 38 | 7396 |

**Abbreviations.** DKA: diabetic ketoacidosis FPG: fasting plasma glucose; ID: insulin dose, CIS: continuous subcutaneous infusion; eGDR: estimated Glucose Disposal rate; eGFR: estimated glomerular filtration rate; BHB: Beta-Hydroxybutyrate;; RIS: relative insulin sensitivity; SGLT2i: SGLT2 inhibitor GTI: genital tract infections; UTI: urinary tract infections

**S1 Table M. Univariable meta-regression for moderators of the RR of volume depletion events**

| **Univariable Meta-Regression: baseline predictors of volume depletion** | | | | | | |
| --- | --- | --- | --- | --- | --- | --- |
| **Patient-related factors** |  | | | | | |
| **Moderator** | **Coefficient** | **95%CI** | **SE** | **P** | **N-comparisons** | **N-participants** |
| **Age(yr)** | 0.016 | -0.068 0.101 | 00.043 | 0.704 | 38 | 7396 |
| **Gender(% M)** | 0.019 | -0.028 0.065 | 0.024 | 0.435 | 38 | 7396 |
| **Ethnicity:** |  | | | | | |
| **Caucasians (%)** | 0.002 | -0.013 0.017 | 0.008 | 0.769 | 38 | 7396 |
| **Asians (%)** | -0.005 | -0.016, 0.005 | 0.005 | 0.315 | 38 | 7396 |
| **Hispanic (%)** | -0.097 | -0.252, 0.063 | 0.071 | 0.295 | 38 | 7396 |
| **Black (%)** | 0.066 | -0.084, 0.216 | 0.077 | **0**.390 | 38 | 7396 |
| **CSI users (%)** | -1.656 | -5.550 2.238 | 1.987 | 0.404 | 38 | 7396 |
| **Total ID (IU/d)** | 0.015 | -0.040 0.070 | 0.028 | 0.589 | 38 | 7396 |
| **Diabetes duration(yr)** | 0.029 | -0.081 0.138 | 0.056 | 0.610 | 38 | 7396 |
| **BMI (kg/m^2^)** | 0.127 | -0.059 0.313 | 0.095 | 0.161 | 38 | 7396 |
| **Sys BP(mmHg)** | 0.005 | -0.014 0.024 | 0.010 | 0.625 | 38 | 7396 |
| **HbA1c(%)** | -0.464 | -1.438, 0.51 | 0.497 | 0.351 | 38 | 7396 |
| **FPG(mg/dL)** | -0.000 | -0.026, 0.026 | 0.013 | 0.991 | 38 | 7396 |
| **Time-in-range(%)** | 0.005 | -0.103 0.114 | 0.055 | 0.925 | 31 | 3050 |
| **MAGE(mg/dL)** | 0.001 | -0.010 0.012 | 0.005 | 0.844 | 31 | 3050 |
| **eGDR(mg/kg/min)** | -0.698 | -1.250, -0.145 | 0.279 | **0.009** | 38 | 7396 |
| **Renal function stage** | -0.341 | -0.827 0.145 | 0.248 | 0.168 | 38 | 7396 |
| **eGFR(ml/kg/1.73 m^2^)** | 0.036 | -0.038, 0.109 | 0.037 | 0.340 | 38 | 7396 |
| **Fasting BHB(mmol/L)** | -2.510 | -6.896 1.877 | 2.238 | 0.262 | 24 | 2538 |
| **Study design-related factors** |  | | | | | |
| **Study duration (wk)** | 0.013 | -0.007 0.033 | 0.010 | 0.207 | 38 | 7396 |
| **Study sample size (N)** | 0.005 | -0.002, 0.011 | 0.003 | 0.143 | 38 | 7396 |
| **SGLT2 inhibitor dose** | 0.469 | -0.031, 1.168 | 0.306 | 0.163 | 38 | 7396 |
| **SGLT2 inhibitor drug:** |  | | | | | |
| **DAPA vs. others** | -0.503 | -1.564 0.557 | 0.541 | 0.352 | 38 | 7396 |
| **EMPA vs. others** | -0.487 | -1.980, 1.007 | 0.762 | 0.523 | 38 | 7396 |
| **CANA vs. others** | -0.111 | -1.277, 1.055 | 0.595 | 0.852 | 38 | 7396 |
| **IPRA vs others** | -0.010 | -0.879 0.859 | 0.443 | 0.982 | 38 | 7396 |
| **SOTA vs. others** | -0.426 | -1.609, 0.758 | 0.604 | 0.481 | 38 | 7396 |
| **Pre-randomization insulin optimization**  **(present vs. absent)** | 0.124 | -0.682, 0.930 | 0.411 | 0.762 | 38 | 7396 |
| **Risk-of-Bias**  **(high/unclear vs. low)** | 0.111 | -0.397, 0.721 | 0.190 | 0.812 | 38 | 7396 |
| **Univariable Meta-Regression: treatment-related predictors of volume depletion** | | | | | | |
| **Moderator** | **Coefficient** | **95%CI** | **SE** | **P** | **N-comparisons** | **N-participants** |
| **Total ID change (%)** | -2.336 | -6.594, 1.923 | 2.173 | 0.282 | 38 | 7396 |
| **Basal ID change (%)** | 0.001 | -0.052, 0.054 | 0.027 | 0.970 | 38 | 7396 |
| **Bolus ID change (%)** | -0.006 | -0.056 0.043 | 0.025 | 0.799 | 38 | 7396 |
| **TID change (%)/baseline BMI ratio** | 115.081 | -103.891, 334.053 | 111.723 | 0.303 | 38 | 7396 |
| **TID change (%)/baseline RIS ratio (IU^2^/kg/d)** | 0.008 | -0.060 0.076 | 0.035 | 0.823 | 38 | 7396 |
| **Residual INS-SGLT2i effect(%)** | -0.003 | -0.050 0.043 | 0.024 | 0.891 | 38 | 7396 |
| **BMI change (%)** | -0.218 | -0.553, 0.116 | 0.170 | 0.200 | 38 | 7396 |
| **SysBP change(mmHg)** | 0.048 | -0.117, 0.214 | 0.084 | 0.569 | 38 | 7396 |
| **eGFR change** | 0.270 | -0.190, 0.731 | 0.235 | 0.249 | 38 | 7396 |
| **FPG change(mg/dL)** | 0.017 | -0.009 0.044 | 0.014 | 0.202 | 38 | 7396 |
| **HBA1c(%) change** | -1.929 | -6.554 2.695 | 2.360 | 0.414 | 29 | 7243 |
| **Time-in-range(%) change** | 0.027 | -0.119, 0.172 | 0.074 | 0.722 | 31 | 3050 |
| **MAGE(mg/dL) change** | -0.008 | -0.048, 0.032 | 0.020 | 0.690 | 31 | 3050 |
| **eGDR change (%)** | 0.133 | 0.030, 0.289 | 0.077 | **0.039** | 38 | 7396 |
| **RIS change (%)** | -0.016 | -0.057 0.026 | 0.021 | 0.458 | 38 | 7396 |
| **BHB change (mmol/L)** | 0.000 | -0.001, 0.001 | 0.001 | 0.912 | 24 | 2538 |
| **DKA** | 0.107 | 0.035, 0.249 | 0.031 | **0.034** | 38 | 7396 |
| **UTIs** | 0.495 | -0.049 1.056 | 0.312 | 0.279 | 38 | 7396 |
| **GTIs** | 0.503 | -0.041, 1.048 | 0.278 | 0.070 | 38 | 7396 |
| **Respiratory infections** | 0.391 | -0.217, 0.998 | 0.231 | 0.529 | 38 | 7396 |
| **Severe hypoglycemia** | 0.321 | -0.214, 0.857 | 0.273 | 0.243 | 38 | 7396 |

**Abbreviations.** DKA: diabetic ketoacidosis FPG: fasting plasma glucose; ID: insulin dose, CIS: continuous subcutaneous infusion; eGDR: estimated Glucose Disposal rate; eGFR: estimated glomerular filtration rate; BHB: Beta-Hydroxybutyrate;; RIS: relative insulin sensitivity; SGLT2i: SGLT2 inhibitor GTI: genital tract infections; UTI: urinary tract infections

**S1 Table N**. Effect of individual SGLT2 inhibitors on different outcomes

| **Outcome** | **SGLT2 inhibitor** | **Effect estimate (95%CI)** | | **statistical significance,**  **N-comparisons, N- participants** | **I^2^** |
| --- | --- | --- | --- | --- | --- |
| **DKA** | **DAPA** | RR | 2.30 (1.17, 4.51) | p=0.02, N=11, 1784 participants | 6% |
|  | **EMPA** |  | 2.28 (1.15, 4.54) | p=0.02, N=11, 1807 participants |  |
|  | **IPRA** |  | 0.65(0.17, 2.43) | p=0.52, N=4, 216 participants |  |
|  | **CANA** |  | 0.65(0.17, 2.43) | p=0.07, N=2, 351 participants |  |
|  | **SOTA** |  | 3.93 (1.94, 7.96) | p=0.0001; N=10, 3238 participants |  |
| **HbA1c(%)** | **DAPA** | MD | -0.39 (-0.51, -0.27) | p<0.00001, N =5, 1672 participants | 5% |
|  | **EMPA** |  | -0.36 (-0.47, -0.25) | p<0.00001, N =11, 1807 participants |  |
|  | **IPRA** |  | -0.36 (-0.57, -0.15) | p=0.0008, N =1, 175 participants |  |
|  | **CANA** |  | -0.27(-0.44, -0.09) | p=0.003, N =2, 351 participants |  |
|  | **SOTA** |  | -0.34(-0.41,-0.27) | p<0.00001, N=10, 3238 participants |  |
| **FPG(mg/dL)** | **DAPA** | MD | -23.11 (-30.37, -15.84) | p<0.00001, N=11, 1784 participants | 15% |
|  | **EMPA** |  | -20.61 (-28.04, -13.17) | p<0.00001, N=11, 1807 participants |  |
|  | **IPRA** |  | -42.85 (-66.34, -19.35) | p=0.00004, N=4, 216 participants |  |
|  | **CANA** |  | -10.49 (-20.25, -0.73) | p=0.04, N=2, 351 participants |  |
|  | **SOTA** |  | -16.98(-22.09, -11.86) | p<0.00001, N=10, 3238 participants |  |
| **Time-in-**  **Range**  **(%)** | **DAPA** | MD | 8.77(6.85, 10.69) | p=0.0002, N=9, 1572 participants | 18% |
|  | **EMPA** |  | 11.39(9.66, 13.12) | p<0.00001, N =11, 871 participants |  |
|  | **IPRA** |  | 2.64(-20.10, 14.83) | p=0.77, N=3, 41 participants |  |
|  | **CANA** |  | 14.34(5.54, 23.14) | p=0.001, N=2, 75 participants |  |
|  | **SOTA** |  | +9.73% (6.66, 12.81) | p<0.00001, N=6, 398participants |  |
| **MAGE**  **(mg/dL)** | **DAPA** | MD | -13.66(-17.29, -10.04) | p=0.0002, N=9, 1572 participants | 19% |
|  | **EMPA** |  | -17.63(-20.64,-14.63) | p<0.00001, N =11, 871 participants |  |
|  | **IPRA** |  | -6.22(-37.57, 25.13) | p=0.70, N=3, 41 participants |  |
|  | **CANA** |  | -27.16(-48.04, -6.29) | p=0.01, N=2, 75 participants |  |
|  | **SOTA** |  | -19.52(-28.91, -10.54) | p<0.0001, N=5, 311 participants |  |
| **Daily Total insulin dose (%)** | **DAPA** | MD | -12.56 (-15.88, -9.25) | p<0.00001, N=11, 1784 participants | 5% |
|  | **EMPA** |  | -10.57(-12.29, -8.85) | p<0.00001, N=11, 1807 participants |  |
|  | **IPRA** |  | -14.99(-18.99, -10.98) | p<0.00001, N=4, 216 participants |  |
|  | **CANA** |  | -10.97 (-17.54, -4.40) | p<0.00001, N=2, 351 participants |  |
|  | **SOTA** |  | -8.99% (-10.93, -7.05) | p<0.00001, N=10, 3238 participants |  |
| **Daily Basal**  **insulin dose**  **(%)** | **DAPA** | MD | -15.42(-18.95, -11.89) | p<0.00001, N=11, 1784 participants | 11% |
|  | **EMPA** |  | -11.90 (-13.97, -9.83) | p<0.00001, N =11, 1807 participants |  |
|  | **IPRA** |  | -21.07 (-27.06, -14.88) | p<0.00001, N=4, 216 participants |  |
|  | **CANA** |  | --17.49 (-23.06, -11.92) | p<0.00001, N=2, 351 participants |  |
|  | **SOTA** |  | -8.03 (-10.14, -5.93) | p<0.00001, N=10, 3238 participants |  |
| **Daily**  **Bolus**  **insulin dose**  **(%)** | **DAPA** | MD | -8.45 (-11.05, -5.84) | p<0.00001, N=11, 1784 participants | 9% |
|  | **EMPA** |  | -13.37 (-17.52, -9.23) | p<0.00001, N=11, 1807 participants |  |
|  | **IPRA** |  | -10.92 (-15.08, -6.76) | P<0.00001, N=4, 216 participants |  |
|  | **CANA** |  | -7.29 (-12.09, -2.51) | P=0.01, N=2, 351 participants |  |
|  | **SOTA** |  | -9.14(-12.17, -6.12 | p<0.00001, N=10, 3238 participants |  |
| **eGDR**  **(%)** | **DAPA** | MD | 10.48 (9.26, 11.70) | p<0.00001, N=11, 1784 participants | 20% |
|  | **EMPA** |  | 12.04 (9.85, 14.28) | p<0.00001, N=11, 1807 participants |  |
|  | **IPRA** |  | 8.08 (4.81, 11.35) | p<0.00001, N=4, 216 participants |  |
|  | **CANA** |  | 10.69 (7.95, 13.44) | p<0.00001, N=2, 351 participants |  |
|  | **SOTA** |  | 11.65% (9.15, 14.15) | p<0.00001, N=10, 3238 participants |  |
| **BMI**  **(%)** | **DAPA** | MD | -3.07(-3.78, -2.36) | p<0.00001, N=11, 1784 participants | 20% |
|  | **EMPA** |  | -3.13(-3.71, -2.56) | p<0.00001, N=11, 1807 participants |  |
|  | **IPRA** |  | -2.07(-4.36, -0.23) | p=0.04, N=4, 216 participants |  |
|  | **CANA** |  | -4.19(-6.11, -2.27) | p<0.00001, N=2, 351 participants |  |
|  | **SOTA** |  | -3.54% (-3.98, -3.09) | p<0.00001, N=10, 3238 participants |  |
| **SystolicBP**  **(mmHg)** | **DAPA** | MD | -3.58(-6.20, -0.96) | p=0.007, N=11, 1784 participants | 9% |
|  | **EMPA** |  | -3.67(-5.13, -2.22) | p<0.00001, N =11, 1807 participants |  |
|  | **IPRA** |  | -2.84(-5.10, -0.59) | p=0.01, N=4, 216 participants |  |
|  | **CANA** |  | -5.61(-8.47, -2.74) | p=0.0001, N=2, 351 participants |  |
|  | **SOTA** |  | -3.85(-4.76, -2.93) | p<0.00001, 3238 participants |  |
| **eGFR**  **(ml/min/1.73 m^2^)** | **DAPA** | MD | -1.11(-2.11,-0.11) | p=0.03, N=11, 1784 participants | 0% |
|  | **EMPA** |  | -0.62(-1.71, 0.46) | p=0.26, N =11, 1807 participants |  |
|  | **IPRA** |  | -0.82(-3.45, 1.82) | p=0.54, N=4, 216 participants |  |
|  | **CANA** |  | -1.00(-3.84, 1.85) | p=0.49, N=2, 351 participants |  |
|  | **SOTA** |  | -0.80 (-1.42, -0.18) | p=0.01, N=10, 3238 participants |  |
| **Albumin-**  **creatinine**  **ratio**  **(ACR)(mg/g)** | **DAPA** | MD | No study |  | 0% |
|  | **EMPA** |  | -7.99(-15.50,  -0.48) | p=0.04, N=3, 75 participants |  |
|  | **IPRA** |  | No study |  |  |
|  | **CANA** |  | No study |  |  |
|  | **SOTA** |  | -14.65 (-2.58, -26.72) | p=0.02, N=5, 2977 participants |  |
| **Hypoglycemia** | **DAPA** | RR | 0.99(0.93, 0.05) | p=0.98, N=11, 1784 participants | 0% |
|  | **EMPA** |  | 0.95 (0.82, 1.08) | p=0.95, p=0.30, N=11, 1807 participants |  |
|  | **IPRA** |  | 1.01(0.75, 1.48) | I^2^=0%, p0.97, N=4, 216 participants |  |
|  | **CANA** |  | 0.91(0.78, 1.18) | p=0.82, N=2, 351 participants |  |
|  | **SOTA** |  | 0.94 (0.89, 1.12) | p=0.63; N=10, 3238 participants |  |
| **Severe**  **hypoglycemia** | **DAPA** | RR | 0.77(0.27, 2.15) | p=0.62, N=11, 1784 participants | 0% |
|  | **EMPA** |  | 0.67(0.31, 1.43) | p=0.30, N=11, 1807 participants |  |
|  | **IPRA** |  | 0.18(0.01, 4.24) | p=0.28, N=4, 216 participants |  |
|  | **CANA** |  | 2.55(0.56, 11.61) | p=0.23, N=2, 351 participants |  |
|  | **SOTA** |  | 0.69 (0.49, 0.98) | p=0.04; N=10, 3238 participants |  |
| **UTI** | **DAPA** | RR | 1.01(0.68, 1.50) | p<0.00001, N=11, 1784 participants | 0% |
|  | **EMPA** |  | 0.97(0.68, 1.38) | p=0.86, N=11, 1807 participants |  |
|  | **IPRA** |  | 0.80(0.26, 2.49) | p=0.70, N=4, 216 participants |  |
|  | **CANA** |  | 2.74(0.62, 12.18) | p=0.18, N=2, 351 participants |  |
|  | **SOTA** |  | 0.97(0.71, 1.33) | p=0.84; N=10, 3238 participants |  |
| **GTI** | **DAPA** | RR | 4.18(2.45, 7.12) | p<0.00001, N=1, 1784 participants | 11% |
|  | **EMPA** |  | 2.82(1.83, 4.36) | p<0.00001, N=11, 1807 participants |  |
|  | **IPRA** |  | 6.84(0.39, 19.33) | p=0.04, N=4, 216 participants |  |
|  | **CANA** |  | 2.27(1.09, 3.96) | p=0.01, N=2, 351 participants |  |
|  | **SOTA** |  | 3.12 (2.14, 4.54) | p<0.00001; N=10, 3238 participants |  |
| **Volume**  **depletion**  **events** | **DAPA** | RR | 1.00(0.39, 2.57) | p=0.99, N=11, 1784 participants | 7% |
|  | **EMPA** |  | 1.41 (0.70, 2.84) | p=0.34, N=11, 1807 participants |  |
|  | **IPRA** |  | 1.19 (0.42, 3.40) | p=0.74, N=4, 216 participants |  |
|  | **CANA** |  | 2.76 (0.32, 23.59) | p=0.35, N=2, 351 participants |  |
|  | **SOTA** |  | : 2.19 (1.10, 4.36) | p=0.03; N=10, 3238 participants |  |
| **Eye disorders** | **DAPA** | RR | 0.62(0.04, 9.39) | p=0.73, N=11, 1784 participants | 18% |
|  | **EMPA** |  | 0.21(0.05, 0.87) | p=0.03, N=11, 1807 participants |  |
|  | **IPRA** |  | 0.18(0.01, 4.24) | p=0.28, N=4, 216 participants |  |
|  | **CANA** |  | 0.50(0.03, 7.78) | p=0.62, N=2, 351 participants |  |
|  | **SOTA** |  | 0.25 (0.06, 1.00) | p=0.05; N=10, 3238 participants |  |
| **MACE** | **DAPA** | RR | 0.87(0.23, 3.31) | p=0.83, N=11, 1784 participants | 0% |
|  | **EMPA** |  | 1.35(0.32, 5.63) | p=0.68, N=11, 1807 participants |  |
|  | **IPRA** |  | 0.92(0.05, 18.50) | p=0.96, N=4, 216 participants |  |
|  | **CANA** |  | 1.53(0.06, 36.88) | p=0.80, N=2, 351 participants |  |
|  | **SOTA** |  | 1.06 (0.40, 2.82) | p=0.91; N=10, 3238 participants |  |

**Abbreviations:** ACR: albumin-to-creatinine ratio; DKA: diabetic ketoacidosis; eGFR: estimated Glumerular filtration rate; FPG: fasting plasma glucose; eGDR: estimated Glucose Disposal rate;

**S1 Table O**. Results of sensitivity analyses with exclusion of RCTs with high risk of bias, with alternative effect measures, pooling methods, and statistical models.

| **Sensitivity analysis: RCTs with high risk of bias excluded [54, 67]** | |
| --- | --- |
| **Outcome** | **Effect estimate (95%CI), I^2^, statistical significance, N-comparisons,**  **N-participants** |
| **DKA** | RR: 2.92(2.00, 4.26), I^2^=0%, p<0.00001, N=33, 7306 participants |
| **HbA1c (%)** | WMD: -0.36(-0.41, -0.32), I^2^=0%, p<0.00001, N =26 comparisons, 7195 participants |
| **FPG(mg/dL)** | WMD: -18.96(-22.17, -15.74), I^2^=4%, p<0.00001, N=33, 7306 participants |
| **Time-in-Range (%)** | WMD: 9.74 (8.53, 10.95]) I^2^=18%, p<0.00001, N=28, 3002 participants |
| **MAGE(mg/dL)** | WMD: -15.78 (-17.86, -13.69) I^2^=0%, p<0.00001, N=28, 3002 participants |
| **Total insulin dose (IU/d)** | WMD: -10.34 (-11.28, -9.39) I^2^=0%, p<0.00001, N=28, 3002 participants |
| **Basal insulin dose (IU/d)** | WMD: -12.16(-13.88, -10.44), I^2^=31%, p<0.00001, N=28, 3002 participants |
| **Bolus insulin dose (IU/d)** | WMD: -9.59(-11.32, -7.85), I^2^=24%, p<0.00001, N=28, 3002 participants |
| **eGDR change (%)** | WMD: 11.17 (10.24, 12.09), I^2^=33%, p=0.001, N=33, 7306 participants |
| **BMI change (%)** | WMD: -3.22(-3.58, -2.86), I^2^=18%, p<0.00001, N=33, 7306 participants |
| **Systolic BP (mmHg)** | WMD: -3.76(-4.45, -3.07) I^2^=0%, p<0.00001, N=33, 7306 participants |
| **eGFR (ml/min/1.73 m2)** | WMD: -0.79(-1.31, -0.27) I^2^=0%, p=0.003, N=33, 7306 participants |
| **Albumin-creatinine ratio (ACR)(mg/g)** | WMD: -10.91(-16.26, -3.55) I^2^=0%, p=0.002, N=33, 7306 participants |
| **Hypoglycemia** | RR: 0.83(0.62, 1.07), I^2^=0%, p0.89, N=33, 7306 participants |
| **Severe hypoglycemia** | RR: 0.81(0.61, 1.07) I^2^=0%, p=0.14, N=33, 7306 participants |
| **UTI** | RR: 0.99(0.81, 1.21])I^2^=0%, p=0.93, N=33, 7306 participants |
| **GTI** | RR: 3.23(2.53, 4.14), I^2^=0%, p<0.00001, N=33, 7306 participants |
| **Volume depletion events** | RR: 1.50(1.10, 2.26), I2=0%, p=0.02, N=33, 7306 participants |
| **Eye disorders** | RR: 0.24(0.09, 0.62), I^2^=0%, p=0.003, N=33, 7306 participants |
| **MACE** | RR: 1.06(0.54, 2.08), I^2^=0%, p=0.87, , N=33, 7306 participants |

| **Sensitivity analysis: Peto Odds Ratio, fixed-effect model** | |
| --- | --- |
| **Outcome** | **Effect estimate (95%CI), I^2^, statistical significance, N-comparisons,**  **N- participants** |
| **DKA** | 3.04(2.23, 4.15), I^2^=0%, p<0.00001, N=38, 7396 participants |
| **Hypoglycemia** | 0.83(0.62, 1.07), I^2^=0%, p0.89, N=38, 7396 participants |
| **Severe hypoglycemia** | 0.86(0.63, 1.16), I^2^=0%, p=0.32, N=38, 7396 participants |
| **Upper respiratory Infections** | 0.91(0.74, 1.10), I^2^=0%, p=0.33, N=38, 7396 participants |
| **UTI** | 1.03 (0.84, 1.27), I^2^=0%, p=0.76, N=38, 7396 participants |
| **GTI** | 2.85(2.36, 3.44), I^2^=0%, p<0.00001, N=38, 7396 participants |
| **Volume depletion events** | 2.14(1.45, 3.16), I2=0%, p=0.0001, N=38, 7396 participants |
| **Renal events** | 1.49(0.92, 2.32), I^2^=0%, p=0.12, N=38, 7396 participants |
| **Venous thromboembolism** | 1.10(0.22, 5.54; I^2^=0%, p=0.91, N=38, 7396 participants |
| **Eye disorders** | 0.16(0.05, 0.50), I^2^=0%, p=0.001, N=38, 7396 participants |
| **MACE** | \|  \| \| --- \|   1.26(0.64, 2.47), I^2^=0%, p=0.50, N=38, 7396 participants |
| **Bone fractures** | 0.85 (0.55, 1.31), I^2^=0%, p=0.45, N=38, 7396 participants |
| **Limb amputation** | 0.61(0.12, 2.98), I^2^=6%, p=0.54, N=38, 7396 participants |
| **Cancer** | 1.03 (0.41, 2.57), I^2^=22%, p=0.95, N=38, 7396 participants |
| **All-cause death** | 0.25(0.05, 1.36), I^2^=0%, p=0.11, N=38, 7396 participants |

**S1 Table P**. Sensitivity analysis: fully-adjusted multivariable meta-regression Model 1 and Model 2 for moderators of the Risk Ratio of Diabetic Ketoacidosis (DKA); variables significantly associated with the risk of DKA (p-value set at 0.15) were entered in Model 3.

| **Multi-variable meta-regression Model 1: baseline predictors of incident DKA** | | | | | | |
| --- | --- | --- | --- | --- | --- | --- |
| **Moderator** | **β(95%CI)** | **SE** | **P** | **R^2^** | **N-comparisons** | **N-participants** |
| **Age(yr)** | 0.002 (-0.181, 0.185) | 0.093 | 0.984 | 59% | 38 | 7396 |
| **Gender(% M)** | -0.001  (-0.061, 0.049) | 0.030 | 0.873 |  | 38 | 7396 |
| **Ethnicity**  **(% Caucasians)** | -0.009  (-0.095, 0.085) | 0.031 | 0.913 |  | 38 | 7396 |
| **CSI users (%)** | -1.501  (-5.941, 0.441) | 0.274 | 0.581 |  | 38 | 7396 |
| **Total ID(IU/d)** | 0.062 (-0.115 0.238) | 0.090 | 0.493 |  | 38 | 7396 |
| **Diabetes duration(yr)** | -0.197(-0.529, 0.135) | 0.135 | 0.245 |  | 38 | 7396 |
| **BMI(kg/m^2^)** | 0.405  (0.184, 0.626) | 0.113 | **0.0001** |  | 38 | 7396 |
| **HbA1c(%)** | -1.907  (-3.909, 0.095) | 1.021 | 0.072 |  | 38 | 7396 |
| **FPG(mg/dL)** | 0.013(-0.034, 0.060) | 0.024 | 0.712 |  | 38 | 7396 |
| **eGDR(mg/kg/min)** | -1.214  (-1.675, -0.753) | 0.235 | **0.0008** |  | 38 | 7396 |
| **Renal function stage** | -0.216  (-0,798, 0.366) | 0.297 | 0.394 |  | 38 | 7396 |
| **Study duration(wk)** | 0.012(-0.370, 0.394) | 0.195 | 0.215 |  | 38 | 7396 |
| **Study sample size (N)** | 0.002( -0.366, 0.370) | 0.188 | 0.719 |  | 38 | 7396 |
| **SGLT2 inhibitor dose** | 0.332 (-0.534, 1.198) | 0.442 | 0.452 |  | 38 | 7396 |
| **SGLT2 inhibitor drug** | -0.171  (-0.939, 0.597) | 0.392 | 0.613 |  | 38 | 7396 |
| **Pre-randomization insulin optimization** | 0.109(-0.310, 0.528) | 0.214 | 0.513 |  | 38 | 7396 |
| **Risk-of-Bias**  **(high vs. low-unclear)** | 0.095(-0.136, 0.326) | 0.118 | 0.782 |  | 38 | 7396 |
| **Multi-variable meta-regression model 2: treatment-related predictors of DKA** | | | | | | |
| **Moderator** | **β(95%CI)** | **SE** | **P** | **R^2^** | **N-comparisons** | **N-participants** |
| **Total ID change (%)** | -0.171( -1.413, 1.071) | 0.634 | 0.786 | **39%** | 38 | 7396 |
| **Basal ID change (%)** | -0.003(-0.800, 0.794 | 0.407 | 0.994 |  | 38 | 7396 |
| **Bolus ID change (%)** | -0.012( 0.046, 0.076) | 0.033 | 0.707 |  | 38 | 7396 |
| **TID change (%)/baseline BMI ratio** | -0.029 (-0.060, 0.002) | 0.013 | 0.211 |  | 38 | 7396 |
| **TID change (%)/baseline RIS ratio (IU^2^/kg/d)** | -0.044(-0.024, -0.064 | 0.010 | **0.0009** |  | 38 | 7396 |
| **Residual INS-SGLT2i effect (%)** | 0.005(-0.050, 0.060) | 0.023 | 0.713 |  | 39 | 7396 |
| **BMI change (%)** | 0.055( -6.191, 6.301) | 3.187 | 0.392 |  | 38 | 7396 |
| **HbA1 change(%)** | 2.202( -22.793 27.197 ) | 12.753 | 0.863 |  | 38 | 7396 |
| **FPG change(mg/dL)** | 0.047(-0.125, 0.219) | 0.088 | 0.593 |  | 38 | 7396 |
| **Time-in-range(%) change** | 0.125(-0.081, 0.331) | 0.105 | 0.236 |  | 31 | 3050 |
| **MAGE(mg/dL) change** | 0.030(-0.064, 0.124) | 0.048 | 0.537 |  | 31 | 3050 |
| **eGDR change (%)** | -0.096( -0.537 , 0.345) | 0.225 | 0.670 |  | 38 | 7396 |
| **RIS change (%)** | -0.011(-0.054 ,0.032) | 0.022 | 0.620 |  | 38 | 7396 |
| **Volume depletion events** | 0.359  (0.199, 0.481) | 0.080 | **0.00007** |  | 38 | 7396 |
| **UTIs** | 0.251  -0.133, 0.635 | 0.196 | 0.519 |  | 38 | 7396 |
| **GTIs** | 0.268 (-0.226, 0.762) | 0.252 | 0.287 |  | 38 | 7396 |
| **Respiratory infections** | 0.113  -0.101, 0.327 | 0.109 | 0.719 |  | 38 | 7396 |
| **Severe hypoglycemia** | 0.179 ( -1.093, 1.451) | 0.649 | 0.783 |  | 38 | 7396 |
| **Multi-variable meta-regression model 3: all predictors of DKA** | | | | | | |
| **Moderator** | **β(95%CI)** | **SE** | **P** | **R^2^** | **N-comparisons** | **N-participants** |
| **BMI(kg/m^2^)** | 0.389  (0.171, 0.607) | 0.111 | **0.009** | **88%** | 38 | 7396 |
| **HbA1c(%)** | -1.145  (-3.886, 1.596) | 1.398 | 0.479 |  | 38 | 7396 |
| **eGDR(mg/kg/min)** | -0.915  (-1.554, -0.276) | 0.326 | **0.001** |  | 38 | 7396 |
| **TID change (%)/baseline RIS ratio (IU^2^/kg/d)** | -0.038(-0.046, -0.030 | 0.004 | **0.0008** |  | 38 | 7396 |
| **Volume depletion events** | 0.307  (0.287, 0.327) | 0.010 | **0.0001** |  | 38 | 7396 |

**Abbreviations.** DKA: diabetic ketoacidosis; FPG: fasting plasma glucose; ID: insulin dose, CIS: continuous subcutaneous infusion; eGDR: estimated Glucose Disposal rate; eGFR: estimated glomerular filtration rate;RIS: relative insulin sensitivity; SGLT2i: SGLT2 inhibitor; GTI: genital tract infections; UTI: urinary tract infections

**S1 Table Q.** Sensitivity analysis: fully-adjusted multivariable meta-regression Model 1 and Model 2 for moderators of HbA1c changes (%); variables significantly associated with HbA1c changes(%) (with p-value set at 0.15) were entered in Model 3.

| **Multi-variable meta-regression model 1: baseline predictors of HbA1c changes (%)** | | | | | | | |
| --- | --- | --- | --- | --- | --- | --- | --- |
| **Moderator** | | **β(95%CI)** | **SE** | **P** | **R^2^** | **N-comparisons** | **N-participants** |
| **Age(yr)** | | 0.001  (-0.016, 0.019) | 0.008 | 0.913 | **59%** | 29 | 7243 |
| **Gender(% M)** | | 0.005  (-0.009, 0.012) | 0.007 | 0.654 |  | 29 | 7243 |
| **Ethnicity**  **(% Caucasians)** | | 0.001  (-0.002, 0.003) | 0.001 | 0.713 |  | 29 | 7243 |
| **CSI users (%)** | | 0.185  (-0.184, 0.555 | 0.184 | 0.398 |  | 29 | 7243 |
| **Total ID (IU/d)** | | 0.006  (-0.012, 0.023) | 0.009 | 0.539 |  | 29 | 7243 |
| **Diabetes duration(yr)** | | 0.001  (-0.014, 0.016) | 0.008 | 0.813 |  | 29 | 7243 |
| **BMI (kg/m^2^)** | | -0.039( -0.098, 0.019) | 0.030 | 0.187 |  | 29 | 7243 |
| **HbA1c(%)** | | -0.019  (-0.164, 0.126 | 0.074 | 0.801 |  | 29 | 7243 |
| **FPG(mg/dL)** | | -0.002  (-0.006, 0.002 | 0.002 | 0.512 |  | 29 | 7243 |
| **eGDR(mg/kg/min)** | | -0.038 (-0.128, 0.053) | 0.046 | 0.417 |  | 29 | 7243 |
| **Renal function stage** | | 0.028  (-0.005, 0.061) | 0.017 | 0.312 |  | 29 | 7243 |
| **Study duration (wk)** | | 0.001(-0.003, 0.004) | 0.002 | 0.513 |  | 29 | 7243 |
| **Study sample size (N)** | | 0.001  (-0.005, 0.007) | 0.003 | 0.285 |  | 29 | 7253 |
| **SGLT2 inhibitor dose** | | -0.094  (-0.149,  -0.039) | 0.028 | **0.0009** |  | 29 | 7243 |
| **SGLT2 inhibitor drug** | | -0.003  (-0.118, 0.112) | 0.060 | 0.816 |  | 29 | 7243 |
| **Pre-randomization insulin optimization** | | 0.090 (-0.034, 0.214) | 0.063 | 0.151 |  | 29 | 7243 |
| **Risk-of-Bias**  **(high/unclear vs. low)** | | 0.111  (-0.392, 0.696) | 0.164 | 0.798 |  | 29 | 7243 |
| **Multi-variable meta-regression model 2: treatment-related predictors of HbA1c changes (%)** | | | | | | | |
| **Moderator** | **β(95%CI)** | | **SE** | **P** | **R^2^** | **N-comparisons** | **N-participants** |
| **Total ID change (%)** | 0.762 (-0.548, 2.073) | | 0.669 | 0.254 | 25% | 29 | 7243 |
| **Basal ID change (%)** | -0.004(-0.020, 0.013) | | 0.008 | 0.652 |  | 29 | 7243 |
| **Bolus ID change (%)** | 0.015(-0.043, 0.073) | | 0.030 | 0.601 |  | 29 | 7243 |
| **TID change (%)/baseline BMI ratio** | 15.168(-38.129, 69.318) | | 22.371 | 0.698 |  | 29 | 7243 |
| **TID change (%)/baseline RIS ratio (IU^2^/kg/d)** | -0.261(-2.719, 2.198) | | 1.254 | 0.835 |  | 29 | 7243 |
| **Residual INS-SGLT2i effect(%)** | 0.004 (-0.064, 0.071 | | 0.034 | 0.915 |  | 29 | 7243 |
| **BMI change (%)** | 0.007(-0.096, 0.110) | | 0.053 | 0.891 |  | 29 | 7243 |
| **FPG change (mg/dL)** | 0.001(-0.006, 0.008) | | 0.004 | 0.773 |  | 29 | 7243 |
| **eGDR change (%)** | -0.053(-0.111, -0.005) | | 0.024 | **0.039** |  | 29 | 7243 |
| **RIS change (%)** | -0.002(-0.011, 0.006) | | 0.004 | 0.566 |  | 29 | 7243 |
| **DKA** | 0.001(-0.014, 0.017) | | 0.008 | 0.815 |  | 29 | 7243 |
| **Volume depletion events** | -0.007(-0.028, 0.014) | | 0.007 | 0.269 |  | 29 | 7243 |
| **UTIs** | -0.011(-0.052, 0.032) | | 0.021 | 0.512 |  | 29 | 7243 |
| **GTIs** | -0.071(-0.212, 0.007) | | 0.072 | 0.761 |  | 29 | 7243 |
| **Respiratory infections** | -0.013(-0.050, 0.024) | | 0.019 | 0.529 |  | 29 | 7243 |
| **Multi-variable meta-regression model 3: all predictors of HbA1c changes (%)** | | | | | | | |
| **Moderator** | **β(95%CI)** | | **SE** | **P** | **R^2^** | **N-comparisons** | **N-participants** |
| **SGLT2 inhibitor dose** | -0.072  (-0.121,  -0.023) | | 0.025 | **0.001** | **65%** | 29 | 7243 |
| **Pre-randomization insulin optimization** | 0.070 (-0.029, 0.169) | | 0.051 | 0.213 |  | 29 | 7243 |
| **eGDR change (%)** | -0.041(-0.095, 0.014) | | 0.028 | 0.139 |  | 29 | 7243 |

**Abbreviations.** DKA: diabetic ketoacidosis FPG: fasting plasma glucose; ID: insulin dose, CIS: continuous

subcutaneous infusion; eGDR: estimated Glucose Disposal rate; eGFR: estimated glomerular filtration rate;

RIS: relative insulin sensitivity; SGLT2i: SGLT2 inhibitor GTI: genital tract infections; UTI: urinary tract

infections

| **S1 Table R. Quality of evidence for clinically relevant efficacy (panel A) and safety (panel B) outcomes: Summary of Findings Table according to the GRADE approach** | | | | | | | | | | | | |  |
| --- | --- | --- | --- | --- | --- | --- | --- | --- | --- | --- | --- | --- | --- |
| **Panel A: SGLT2 inhibitors compared to placebo for type 1 diabetes: efficacy outcomes** | | | | | | | | | | | | |  |
| Outcomes | | **Anticipatedabsoluteeffects^*^**(95% CI) | | | | Relative effect (95% CI) | № of participants (studies) | | Certainty of the evidence (GRADE) | | Comments | |  |
|  |  | **Risk with placebo** | | **Risk with SGLT2 inhibitors** | |  |  |  |  |  |  |  |  |
| Mean change in HbA1c(%) follow up: range 4 weeks to 52 weeks | | The mean change in HbA1c(%) ranged from **-0.99 to +0.17** % | | MD **0.37 % lower** (0.41 lower to 0.33 lower) | | - | 7243 (16RCTs) | | ⨁⨁⨁⨁ HIGH | | large effect  dose-response gradient across different doses | |  |
| Mean change in % time-in-range (70-180 mg/d) follow up: range 2 weeks to 52 weeks | | The mean change in time-in-range(%)) ranged from **-7 to +7.5%** % | | MD **9.87 % higher** (8.75 higher to 10.99 higher) | | - | 3050 (14RCTs) | | ⨁⨁⨁⨁ HIGH | | dose-response gradient across different doses | |  |
| Mean change in eGDR(%)  follow up: range 1 week to 52 weeks | | The mean change in eGDR ranged from **-1.5 to +2.3** % | | MD **11.06 % higher** (10.16 higher to 11.96 higher) | | - | 7396 (18 RCTs) | | ⨁⨁⨁⨁ HIGH | | dose-response gradient across different doses | |  |
| Mean change in BMI (%)  follow up: range 1 week to 52 weeks | | The mean change in BMI ranged from **-0.39 to +1.33** % | | MD **3.2 % lower** (3.54 lower to 2.86 lower) | | - | 7396 (18 RCTs) | | ⨁⨁⨁⨁ HIGH | | dose-response gradient across different doses | |  |
| Mean change in systolic blood pressure (BP)(mmHg) follow up: range 1 week to 52 weeks | | The mean change in sysBP ranged from **-7.9 to +3.7** mmHg | | MD **3.8 mmHg lower** (4.48 lower to 3.11 lower) | | - | 7396 (18 RCTs) | | ⨁⨁⨁⨁ HIGH | | dose-response gradient across different doses | |  |
| Mean change in eGFR (ml/min/1.73 m^2^) follow up: range 1 week to 52 weeks | | The mean change ineGFR ranged from **-3.84 to +0.92** ml/min/1.73 m2 | | MD **0.78 ml/min/1.73 m2 lower** (1.29 lower to 0.26 lower) | | - | 7396 (18 RCTs) | | ⨁⨁⨁⨁ HIGH | |  | |  |
| Mean change in urinary albumin/creatinine ratio (ACR)(mg/g)  follow up: range 4 weeks to 52 weeks | The mean change in ACR(mg/g) ranged from 4.1 to 14.9 mg/g | | MD 9.91 mg/g lower (16.26 lower to 3.55 lower) | | - | | | 3052 (4RCTs) | | ⨁⨁⨁⨁ HIGH | | dose-response gradient across different doses |  |
| **Panel B: SGLT2 inhibitors compared to placebo for type 1 diabetes: safety outcomes** | | | | | | | | | | | | | |
| Incidence of diabetic ketoacidosis (DKA) follow up: range 1 week to 52 weeks | 7 per 1.000 | | **21 per 1.000** (14 to 30) | | **RR 2.83** (1.97 to 4.06) | | | 7396 (18 RCTs) | | ⨁⨁⨁⨁ HIGH | | largeeffect | |
| Incidence of severe hypoglycemia follow up: range 1 week to 52 weeks | 30 per 1.000 | | **24 per 1.000** (18 to 32) | | **RR 0.81** (0.61 to 1.07) | | | 7396 (18 RCTs) | | ⨁⨁⨁⨁ HIGH | |  | |
| Incidence of urinary tract infections(UTIs) follow up: range 1 week to 52 weeks | 56 per 1.000 | | **55 per 1.000** (45 to 67) | | **RR 0.99** (0.81 to 1.21) | | | 7396 (18 RCTs) | | ⨁⨁⨁⨁ HIGH | |  | |
| Incidence of genital tract infections(GTIs) follow up: range 1 week to 52 weeks | 26 per 1.000 | | **84 per 1.000** (65 to 107) | | **RR 3.18** (2.49 to 4.06) | | | 7396 (18 RCTs) | | ⨁⨁⨁⨁ HIGH | | Largeeffect | |
| Incidence of major adverse cardiovascular events (MACE)  follow up: range 1 week to 52 weeks | 4 per 1.000 | | **5 per 1.000** (2 to 9) | | **RR 1.06** (0.54 to 2.08) | | | 7396 (18 RCTs) | | ⨁⨁⨁◯ MODERATE^a^ | | Few events, OIS not reached | |
| Incidence ofeye disorders  follow up: range 1 week to 52 weeks | 4 per 1.000 | | **1 per 1.000** (0 to 3) | | **RR 0.27** (0.11 to 0.67) | | | 7396 (18 RCTs) | | ⨁⨁⨁⨁ HIGH | |  | |
| Incidence ofvolume depletion events  follow up: range 1 week to 52 weeks | 8 per 1.000 | | **12 per 1.000** (8 to 19) | | **RR 1.53** (1.03 to 2.28) | | | 7396 (18 RCTs) | | ⨁⨁⨁⨁ HIGH | |  | |
| ***The risk in the intervention group** (and its 95% confidence interval) is based on the assumed risk in the comparison group and the **relative effect** of the intervention (and its 95% CI).   **CI:**Confidenceinterval; **MD:**Meandifference; **RR:**Risk ratio | | | | | | | | | | | | | |
| **GRADE Working Group grades of evidence** **High certainty:** We are very confident that the true effect lies close to that of the estimate of the effect **Moderate certainty:** We are moderately confident in the effect estimate: The true effect is likely to be close to the estimate of the effect, but there is a possibility that it is substantially different **Low certainty:** Our confidence in the effect estimate is limited: The true effect may be substantially different from the estimate of the effect **Very low certainty:** We have very little confidence in the effect estimate: The true effect is likely to be substantially different from the estimate of effect | | | | | | | | | | | | | |

#### Explanations

a. downgraded for imprecision. For calculation of the Optimal Information Size ((OIS), we chose an α of 0.05, a β of 0.2 and a relative risk reduction of 20%
