# Supplementary material for: Assessing the risk of ketoacidosis due to sodium-glucose cotransporter (SGLT)-2 inhibitors in patients with type 1 diabetes: A meta-analysis and meta-regression
Source: PLoS Med. 2020 Dec 29;17(12):e1003461. doi: 10.1371/journal.pmed.1003461 (PMC7771708; doi:10.1371/journal.pmed.1003461)
Supplement: S1 Figs — Fig A in S1 Figs. Risk of bias summary: risk of bias item for each included RCT according to Cochrane Risk-of-Bias Tool. Fig B in S1 Figs. Risk of bias graph: Each risk of bias item is presented as percentages across all included RCTs. Fig C in S1 Figs. Funnel plots for main effect outcomes. Fig D in S1 Figs. Forest plot of comparison: SGLT2 inhibitors versus control, outcome: fasting plasma glucose (FPG), continuous glucose monitoring (CGM) parameters time-in-range (70–180 mg/dL) and mean amplitude of glucose excursions (MAGE) and urinary glucose excretion (UGE). Fig E in S1 Figs. Forest plot of comparison: SGLT2 inhibitors, outcome: daily total, basal, and bolus insulin dose (%) changes from baseline. Fig F in S1 Figs. Forest plot of comparison: SGLT2 inhibitors versus control, outcomes: estimated glucose disposal rate (eGDR) changes (%) (panel 1) and relative insulin sensitivity (RIS) changes (%) (panel 2). Fig G in S1 Figs. Forest plot of comparison: SGLT2 inhibitors versus control, outcomes: body mass index (BMI) and systolic BP (sysBP). Fig H in S1 Figs. Forest plot of comparison: SGLT2 inhibitor versus control versus placebo, outcomes: eGFR and urinary albumin/creatinine ratio (ACR). Fig I in S1 Figs. Forest plot of comparison: SGLT2 inhibitors, outcome: hypoglycemia, severe hypoglycemia, urinary tract infections (UTIs), genital tract infections (GTIs), volume depletion events, eye disorders, and major adverse cardiovascular events (MACE). Fig L in S1 Figs. leave-one-out meta-analysis for outcomes DKA and HbA1c (%). (DOC) [file pmed.1003461.s003.doc]

**S1 Figs**

**S1 Fig A.** Risk of bias summary: risk of bias item for each included RCT according to

Cochrane Risk-of-Bias Tool

**S1 Fig B. Risk of bias graph**: each risk of bias item is presented as percentages across all included RCTs.

**S1 Fig C. Funnel plots for main effect outcomes.**

In each plot, the diagonal lines represent upper and lower 95%CI and the vertical line represents overall effect size.

**S1 Fig C Panel 1** Funnel plot of comparison: DKA outcome: incident DKA

**S1 Fig C Panel 2** Funnel plot of comparison: HbA1c(%) outcome: HbA1c changes (%)

**S1 Fig C Panel 3**. Funnel plot of comparison: % time-in-range (70-180 mg/dL) for outcome: % time-in-range


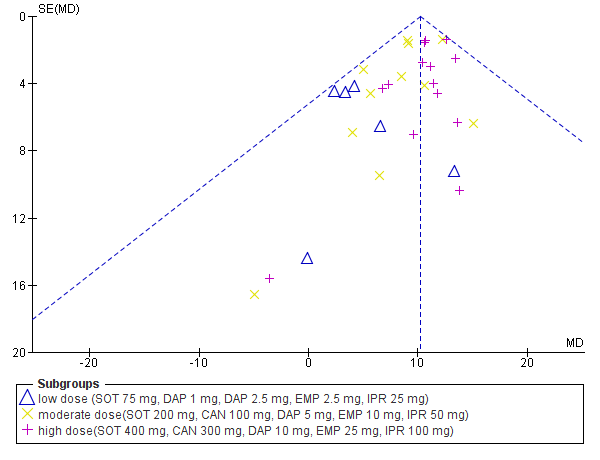


**S1 Fig C Panel 4**. Funnel plot of comparison: estimated Glucose Disposal Rate (eGDR), outcome: eGDR (% change)


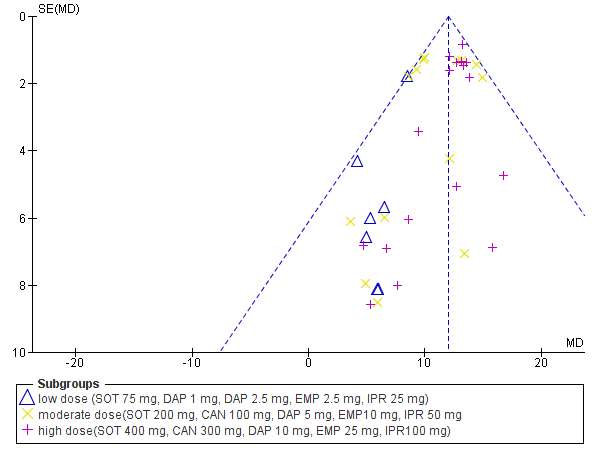


**S1 Fig C Panel 5**. Funnel plot of comparison: BMI changes, outcome: BMI changes (%)


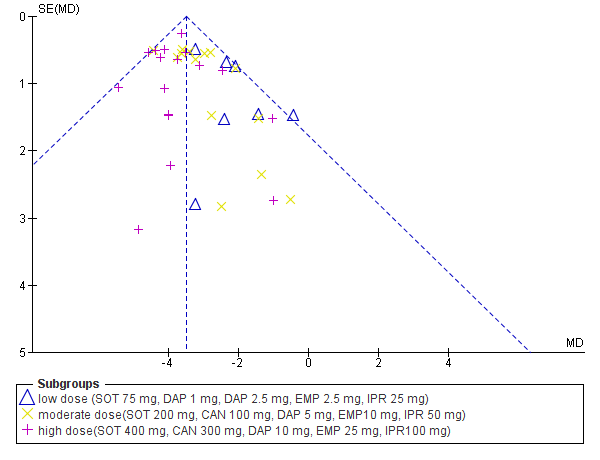


**S1 Fig C Panel 6**. Funnel plot of comparison: sys BP, outcome: sys BP (mmHg)


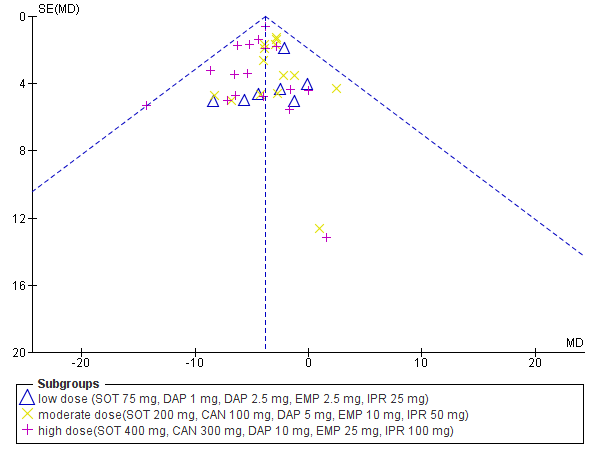


**S1 Fig C Panel 7**. Funnel plot of comparison: eGFR changes, outcome: eGFR changes(ml/min/1.73 m2)

**
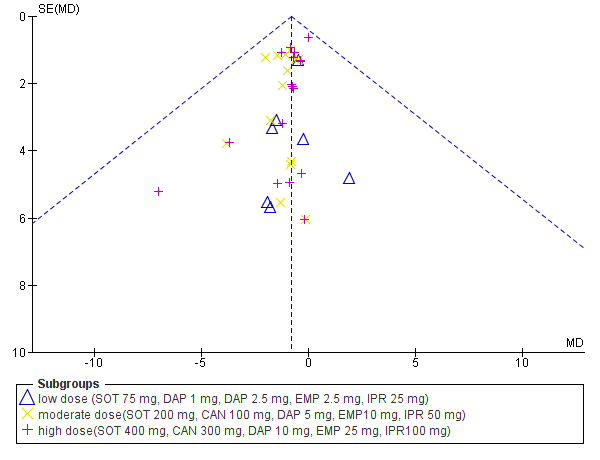
**

**S1 Fig C Panel 8**. Funnel plot of comparison: urinary A/C ratio, outcome: albumin/creatinine ratio(mg/g).


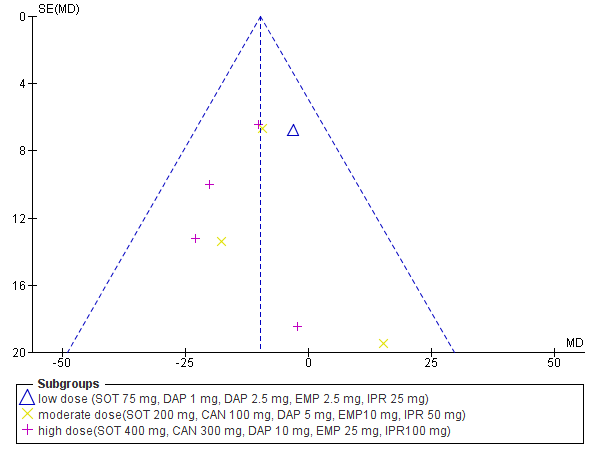


**S1 Fig C Panel 9**. Funnel plot of comparison: severe hypoglycemia, outcome: severe hypoglycemia.


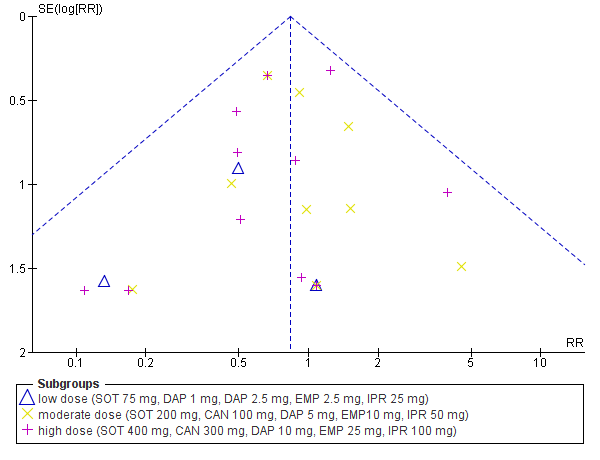


**S1 Fig C Panel 10**. Funnel plot of comparison: urinary tract infections, outcome: urinary tract infections.


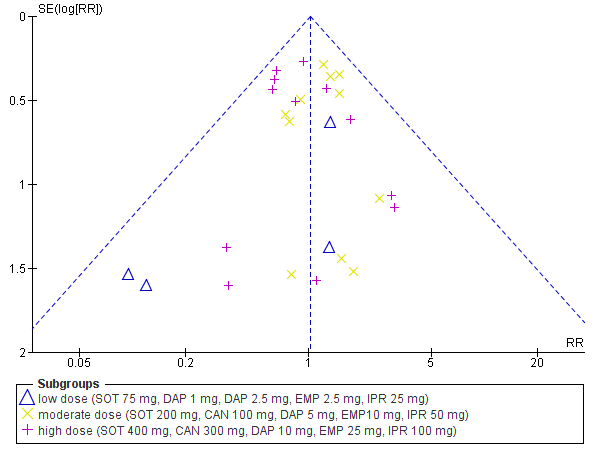


**S1 Fig C Panel 11**. Funnel plot of comparison: genital tract infections, outcome: genital tract infections.


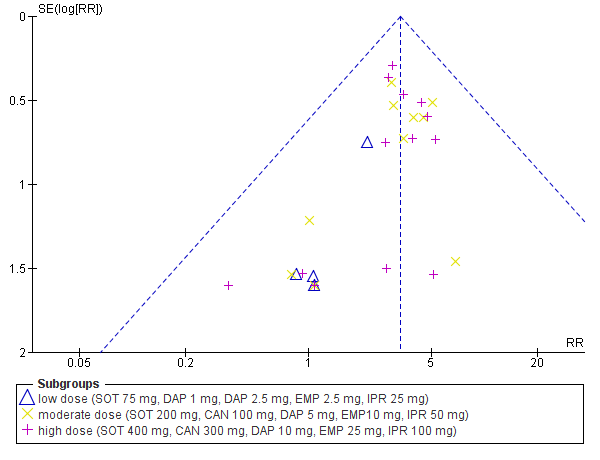


**S1 Fig C Panel 12:** Funnel plot of comparison: MACE, outcome: MACE


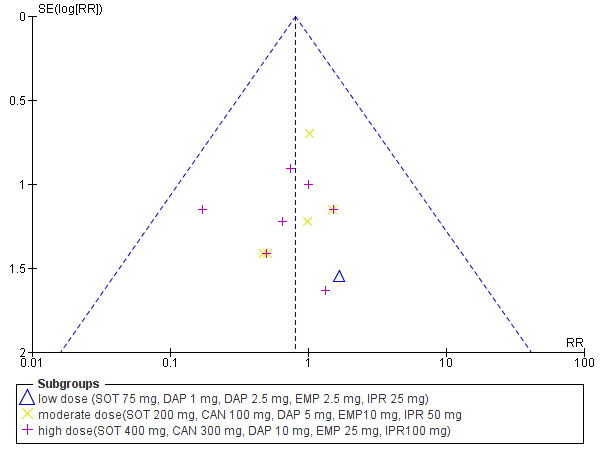


**S1 Fig C Panel 13**. Funnel plot of comparison: eye disorders; outcome: eye disorders


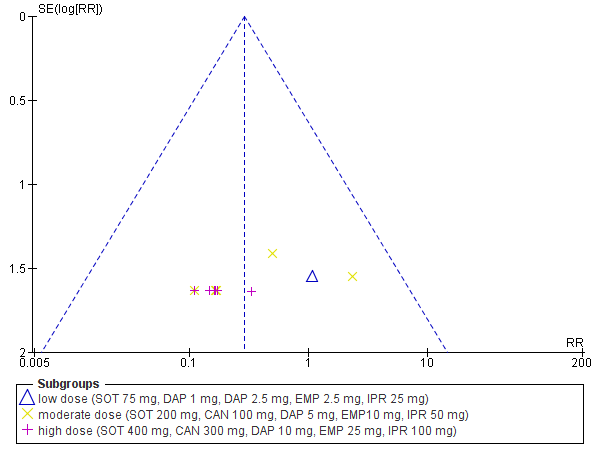


**S1 Fig C Panel 14**. Funnel plot of comparison: volume depletion events, outcome: volume depletion events.


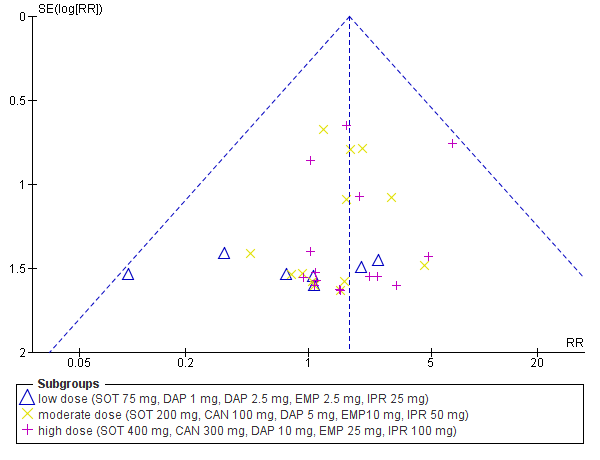


**S1 Fig D.** Forest plot of comparison: SGLT2 inhibitors vs. control, outcome: Fasting Plasma Glucose (FPG), Continuous Glucose Monitoring (CGM) parameters time-in-range (70-180 mg/dL) and Mean Amplitude of Glucose Excursions (MAGE) and urinary glucose excretion(UGE).

**S1 Fig D Panel 1: FPG changes from baseline (mg/dL)**

**S1 Fig D Panel 2: outcome: CGM parameter time-in-range (70-180 mg/dL) (%)**

**S1 Fig D Panel 3: outcome: CGM parameter mean amplitude of glucose excursion (MAGE) (mg/dL)**

**S1 Fig D Panel 4: outcome: urinary glucose excretion (g/24 hr).**

**S1 Fig E.** Forest plot of comparison: SGLT2 inhibitors, outcome: Daily total, basal and bolus insulin dose (%) changes from baseline.

**S1 Fig E Panel 1: outcome: daily total insulin dose(TID) (%) changes**

**
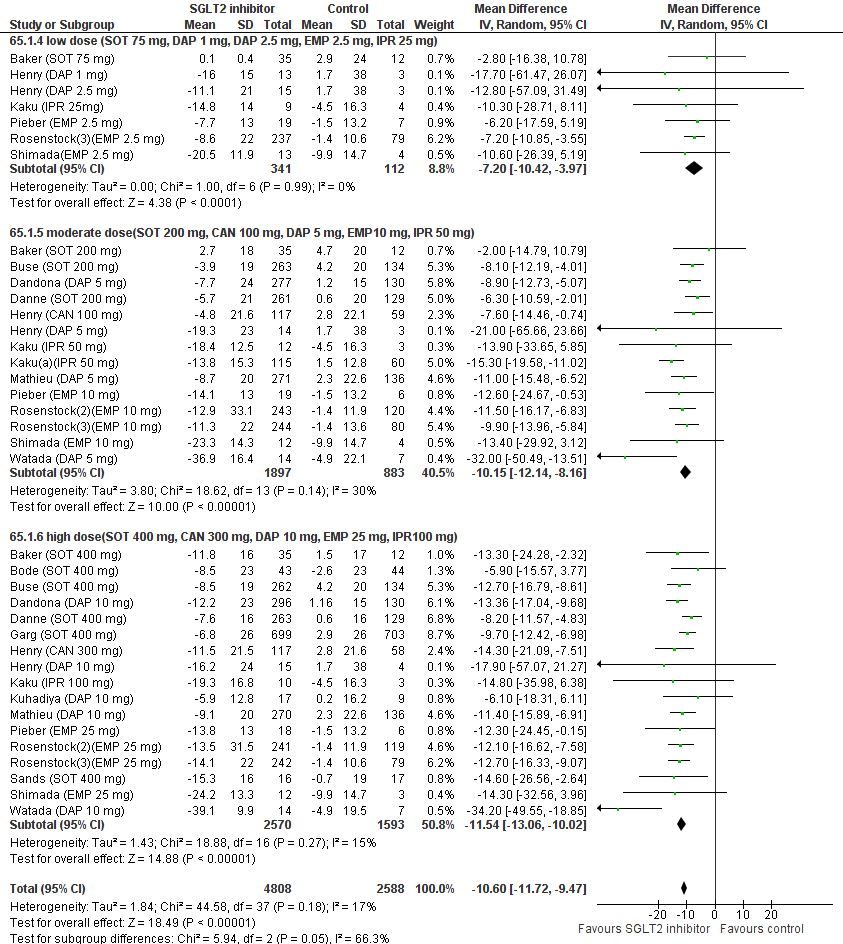
**

**S1 Fig E Panel 2: outcome: daily basal insulin dose (%)**

**
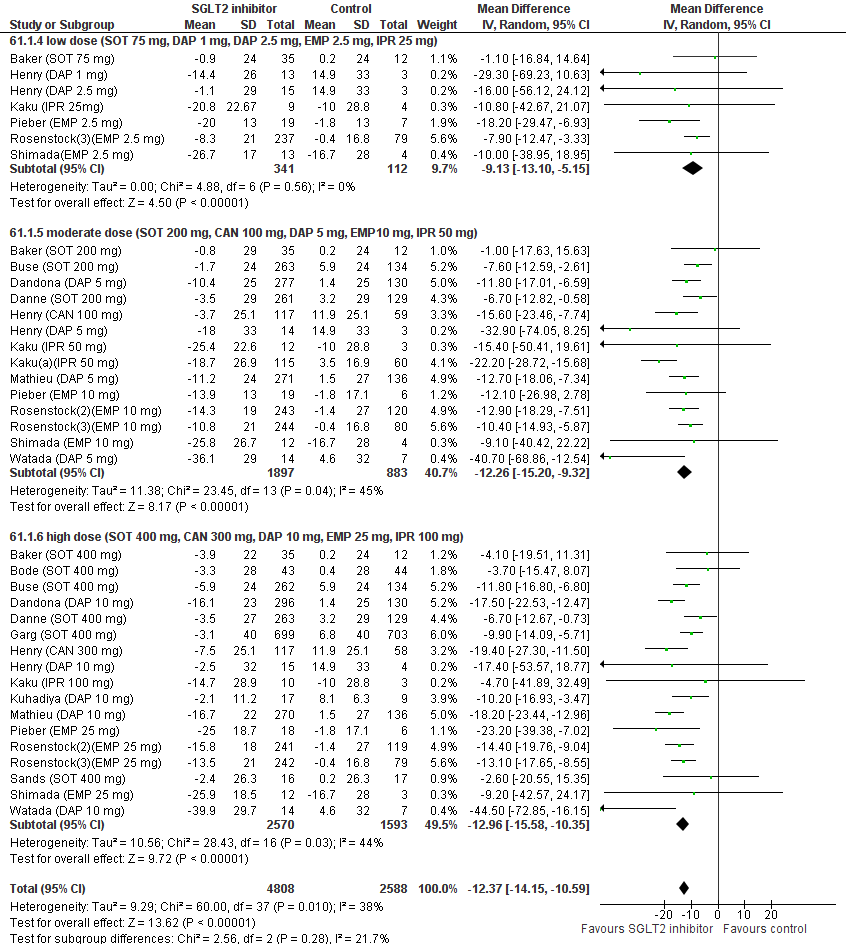
**

**S1 Fig E Panel 3: outcome: daily bolus insulin dose (%)**

**
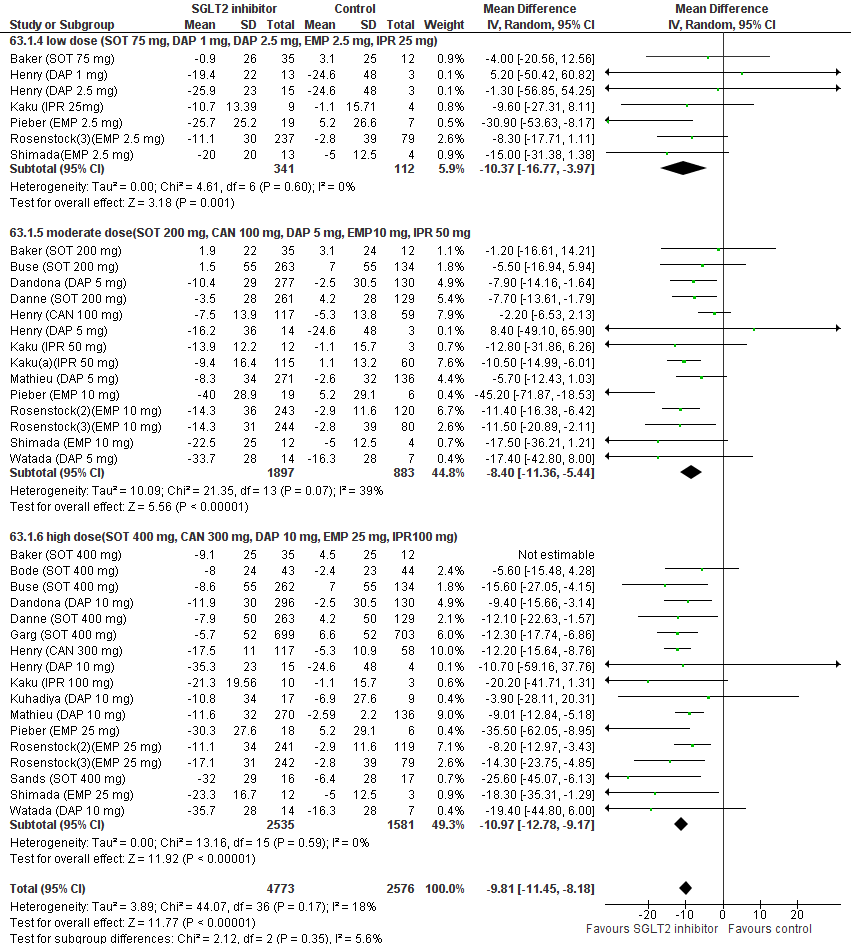
**

**S1 Fig F:** Forest plot of comparison: SGLT2 inhibitors vs. control, outcomes: estimated Glucose Disposal Rate (eGDR) changes (%)(panel 1) and Relative Insulin Sensitivity (RIS) changes(%)(panel 2).

**S1 Fig F Panel 1: eGDR changes (%)**

**
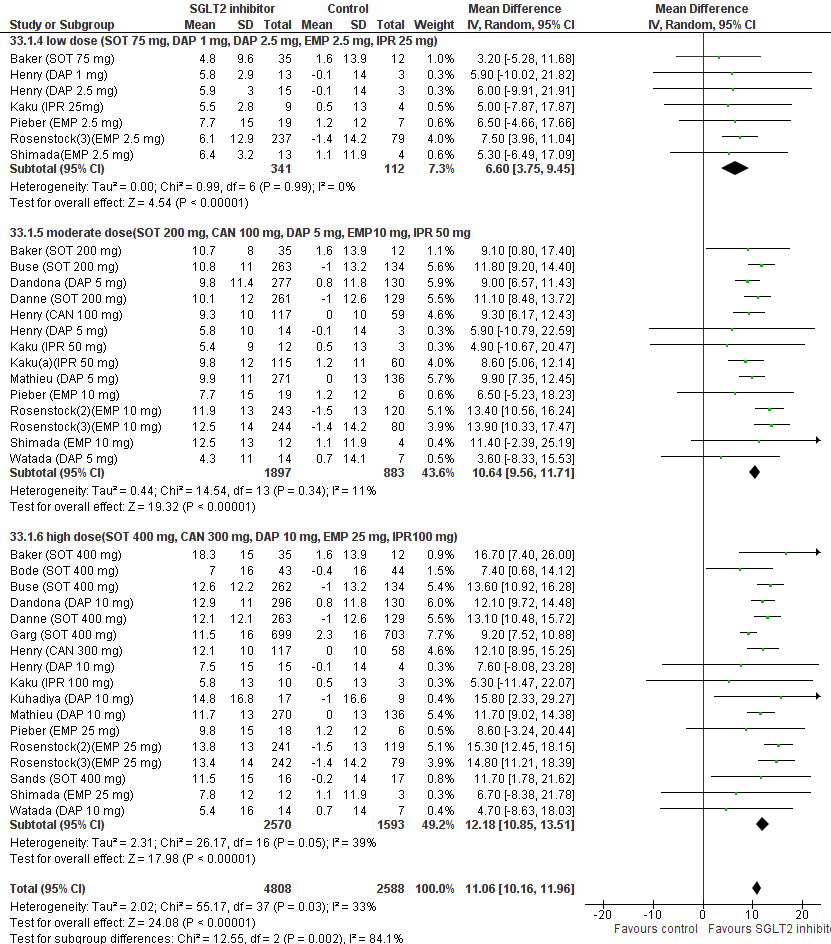
**

**S1 Fig F Panel 2: RIS changes (%)**

**S1 Fig G**. Forest plot of comparison: SGLT2 inhibitors vs. control, outcomes: body mass index (BMI), and systolic BP (sysBP).

**S1 Fig G Panel 1: BMI changes (%)**

**
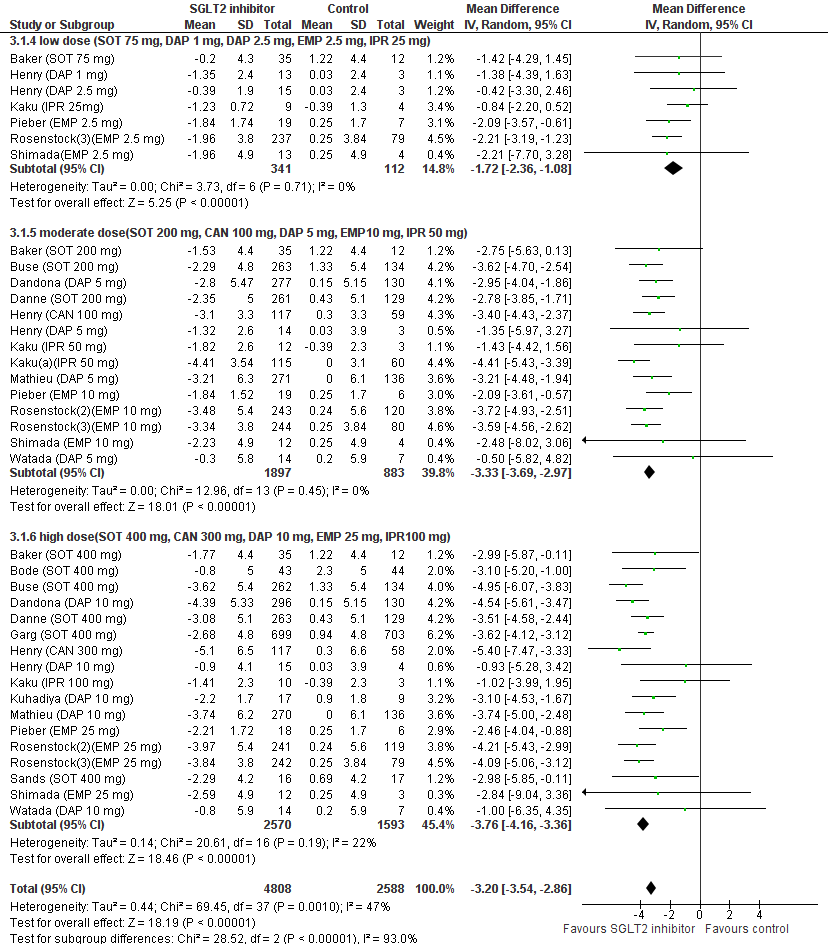
**

**S1 Fig G Panel 2: outcome: sysBP changes (mmHg)**

**S1 Fig H.** Forest plot of comparison: SGLT2 inhibitor vs control vs. placebo, outcomes: eGFR, and urinary Albumin/Creatinine Ratio (ACR).

**S1 Fig H Panel 1: outcome: eGFR changes from baseline (ml/min/1.73m^2^)**

**
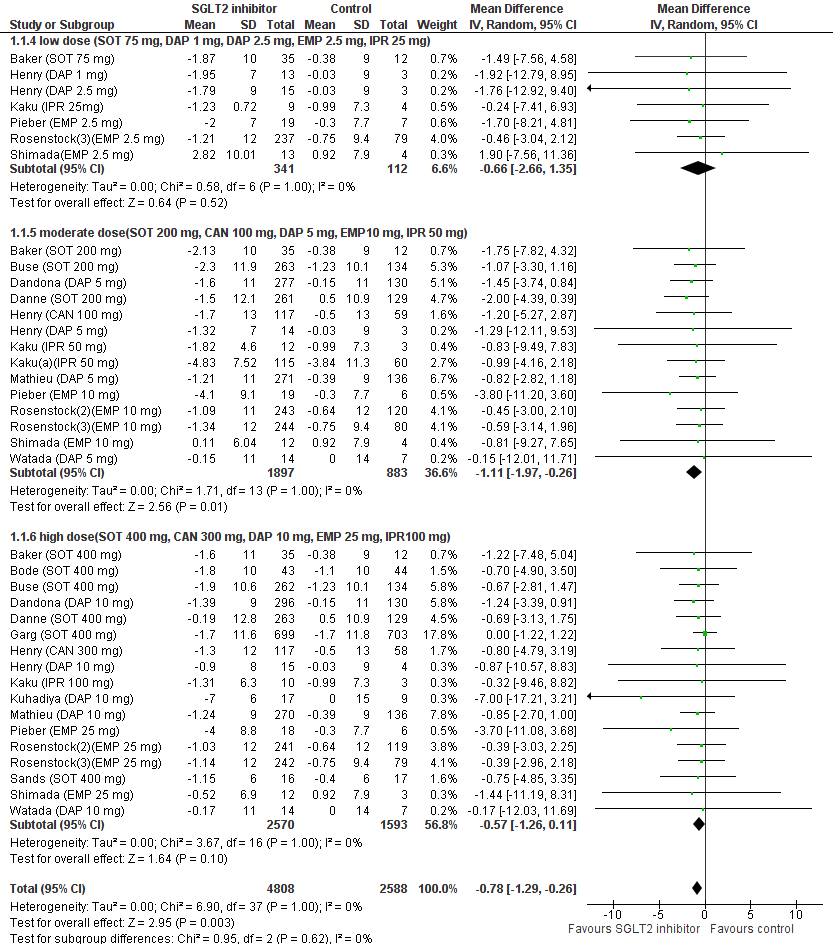
**

**S1 Fig H Panel 2: outcome: ACR changes from baseline (mg/g)**

**S1 Fig I.** Forest plot of comparison: SGLT2 inhibitors, outcome: Hypoglycemia, Severe

Hypoglycaemia, Urinary Tract Infections (UTIs), Genital Tract Infections (GTIs), volume depletion

events, eye disorders and Major Adverse Cardiovascular Events (MACE).

**S1 Fig I Panel 1: Hypoglycemia**

**S1 Fig I Panel 2: Severe Hypoglycemia**

**
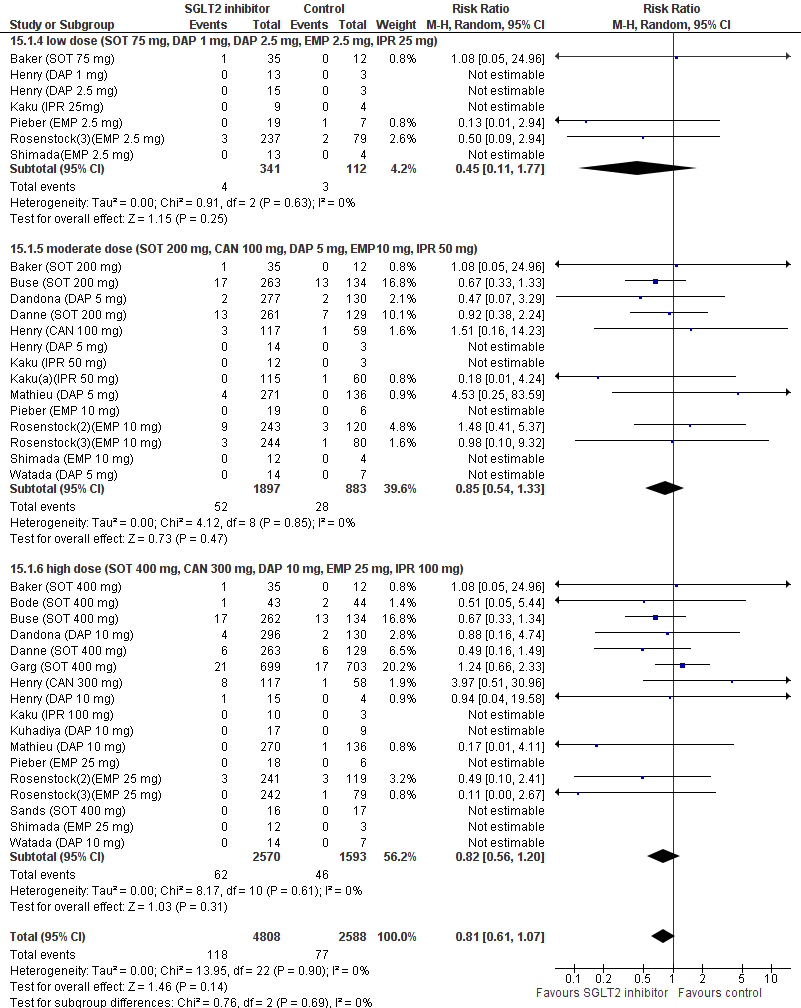
**

**S1 Fig I Panel 3: outcome: UTIs**

**
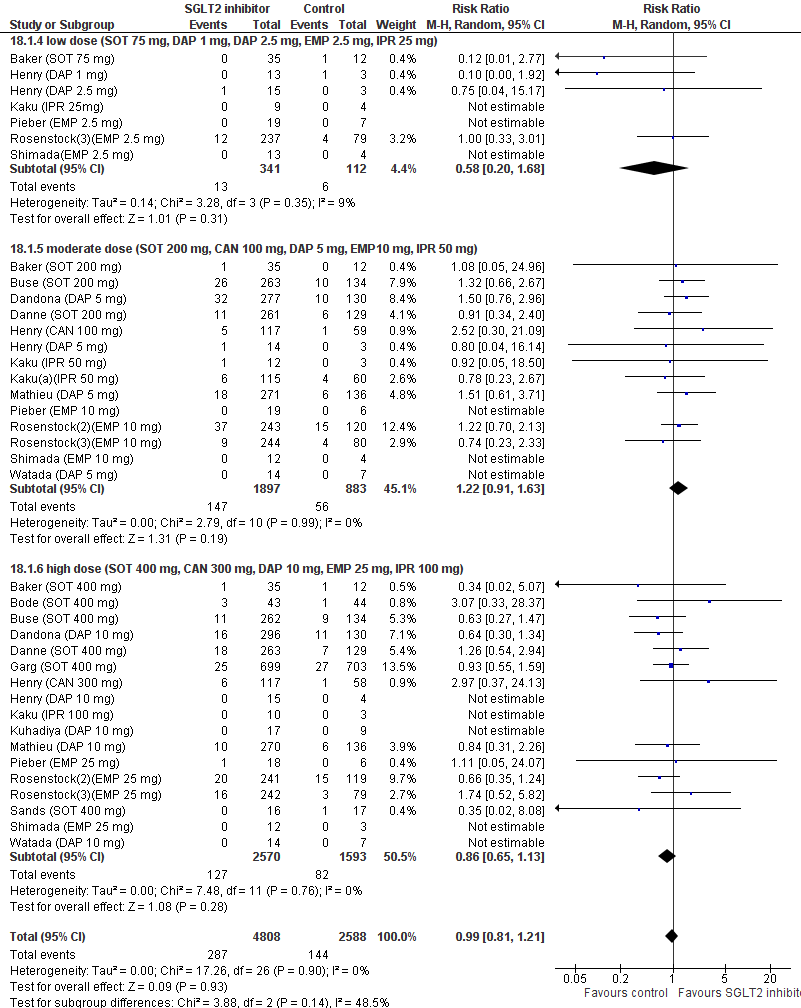
**

**S1 Fig I Panel 4: outcome: GTIs**

**
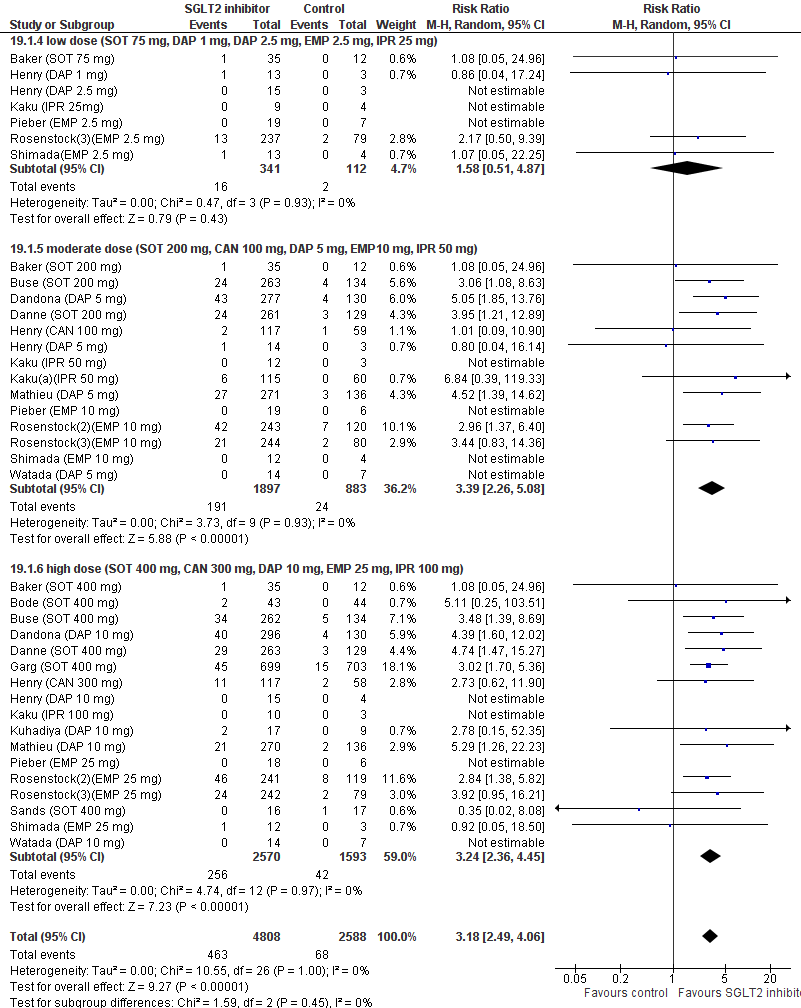
**

**S1 Fig I Panel 5: outcome: volume depletion events**

**S1 Fig I Panel 6: outcome: diabetic eye disorders**

**S1 Fig I Panel 7: outcome: MACE**

**S1 Fig L:** leave-one out meta-analysis for outcomes DKA and HbA1c(%)

**S1 Fig L Panel 1: outcome DKA**


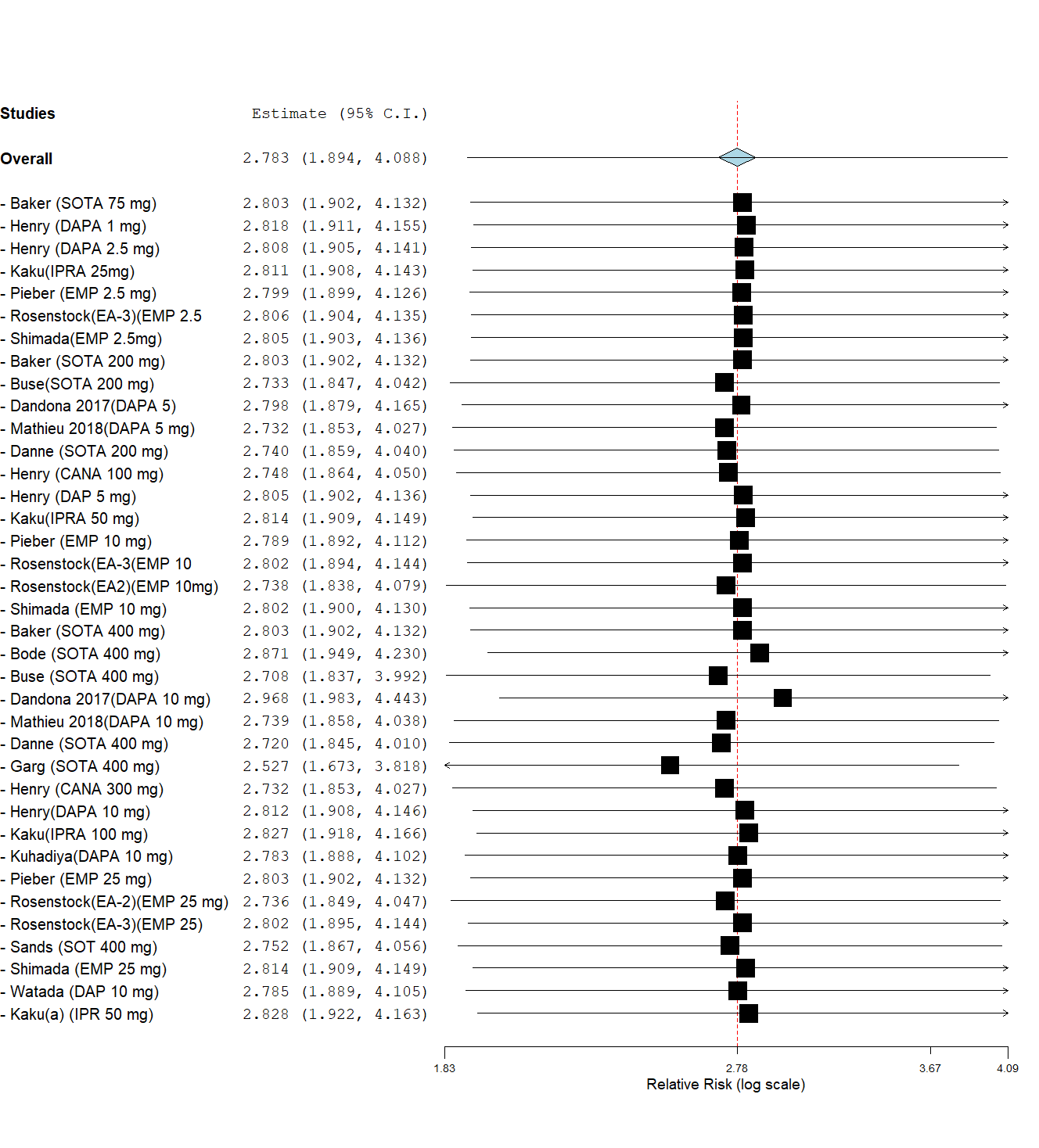


**S1 Fig L Panel 2: outcome: HbA1c (%)**

**
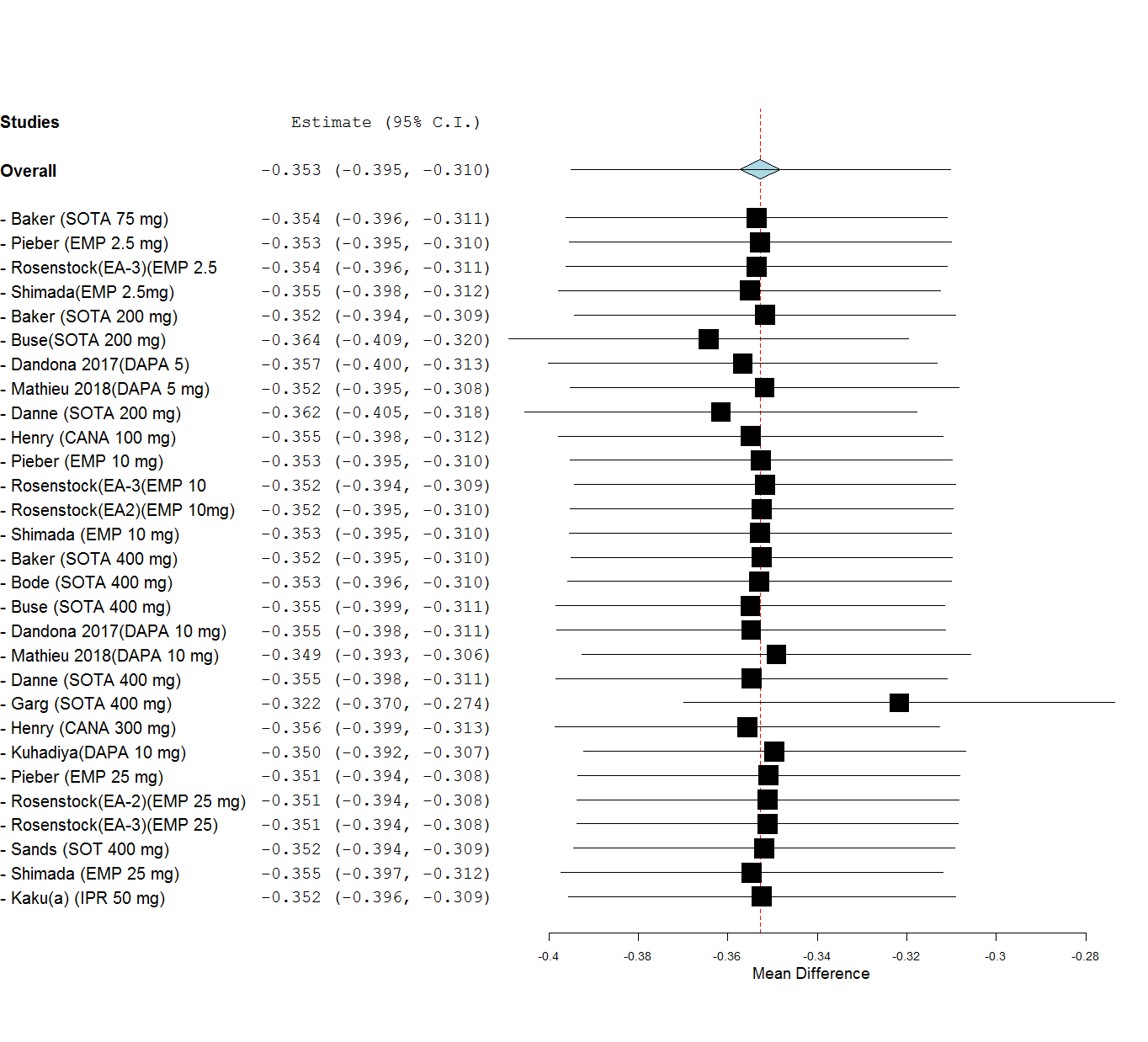
**
